# Axially Chiral Spiro Compounds with Heavier Group 14 Elements as Spiro-Centers

**DOI:** 10.1021/acs.inorgchem.6c00576

**Published:** 2026-05-02

**Authors:** Aynura Mammadova, Clemens Bruhn, Rudolf Pietschnig

**Affiliations:** Institute for Chemistry and CINSaT, 9178 University of Kassel, Heinrich-Plett-Straße 40, Kassel 34132, Germany

## Abstract

Homo- and heteroditopic 4-*tert*-butyl-1,2-benzene-bischalcogenol ligands, namely *t*-BuC_6_H_3_(SH)_2_ and *t*-BuC_6_H_3_(SH)­(SeH) (*t*-Bu = C­(CH_3_)_3_), have been prepared through the functionalization of the *ortho* position of the *t*-BuC_6_H_4_(SH) precursor with thiol (−SH) or selenol (−SeH) groups. The lithiated derivatives of these compounds have been isolated and structurally characterized. With these versatile precursors at hand, we explored the preparation of axially chiral compounds ((*t*-BuC_6_H_3_S_2_)_2_E; (*t*-BuC_6_H_3_(S)­(Se))_2_E; E = Sn; Ge; Pb) with a group 14 element in the center. All complexes have been characterized by multinuclear NMR spectroscopy, EA, HR-MS spectrometry, and SCXRD in the case of the germanium compound. In the case of lead, we observed reduction of Pb­(IV) to Pb­(II) with oxidation of the ligand. The resulting Pb­(II) products were isolated and characterized. Their tendency for aggregation is pronounced, and reaction with donor molecules such as pyridine or 1,3,4,5-tetramethylimidazol-2-ylidene yields adducts, albeit without deaggregation of the polymeric plumbylene in the solid state. For the stable germanes (*t*-BuC_6_H_3_(S)­(Ch))_2_Ge with (Ch = S, Se), additional attempts to achieve chiral resolution were explored using high-performance liquid chromatography (HPLC).

## Graphical abstract

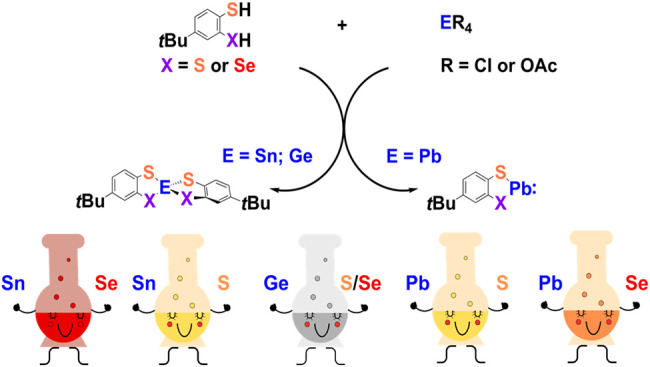

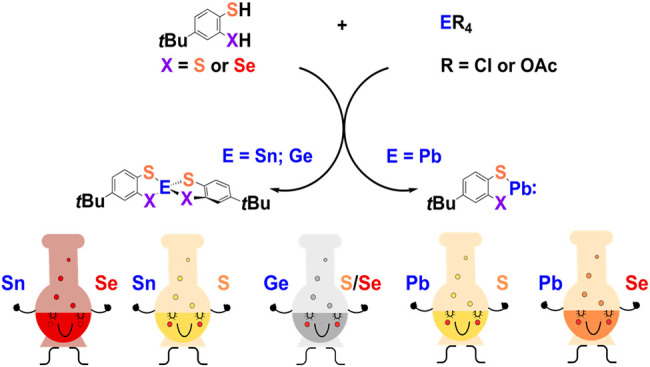

## Introduction

Axial chirality originates from the nonplanar arrangement of four substituents around a chirality axis, as defined by the International Union of Pure and Applied Chemistry (IUPAC).^1,2^ The preparation of volatile chiral compounds, especially those containing heavier elements, poses several challenges especially when the successive exchange of these elements needs to be done at a specific position within the molecule. Allenes are well-known as a prominent class of axially chiral molecules in organic chemistry.^3–6^ Replacing the carbon atoms in these structures with heavier elements from the periodic table, results in significant changes to their bonding properties, geometric configurations, and, most remarkably, their reactivity and stability.^7^ Despite these challenges, compounds of this nature have been synthesized and examined.^8,9^ However, isolating these substances often requires the presence of sterically hindered substituents, which considerably lower their volatility and pose challenges for gas-phase investigations.^10–13^ In this study, we concentrated on the synthesis of chiral spiro-compounds embedding a group 14 element as a spiro-center between *tert*-butyl substituted benzene*-ortho*-bischalcogenolate ligands. Toluene-3,4-dithiolate, and its unsubstituted analog, benzene-1,2-dichalcogenolate, have been employed in prior studies.^14–26^ They were employed owing to their electronic behavior, their redox chemistry, and applications in the design of molecular devices or catalysts.^17,27,28^ The *tert*-butyl substituted benzene-*ortho*-dithiolate tin­(IV) complexes, incorporating either a quaternary ammonium or phosphonium cation have been published in a patent alongside other metal complexes as part of a composition intended for use as light-absorbing material.^29^ Moreover, for the tetravalent group 14 toluene-3,4 dithiolates remarkable optical rotations have been measured in the UV spectral region.^21^ Furthermore, spirobistoluenediselenagermoles and spirobismethoxybenzodiselenagermoles were isolated and characterized as racemic mixtures by Tavares et al.^30^ Expanding on these findings, we have extended the examination to structurally related group 14 elements, such as Sn­(IV), Ge­(IV), and Pb­(IV) with *tert*-butyl substituted benzene-bischalcogenols (*t*-BuC_6_H_3_(SH)_2_ and *t*-BuC_6_H_3_(SH)­(SeH)) (Figure 1). These compounds are intended as model compounds for experiments aiming at the detection of parity violating (PV) effects in molecular physics. For the latter, scaling with the atomic number has been predicted, highlighting the relevance of chiral compounds involving heavy elements.^31^ As most spectroscopic approaches targeting PV effects require enantiopure or at least enantioenriched samples, the pronounced steric demand of the *tert*-butyl group can be anticipated to improve chiral discrimination and possibly enantiomer separation via chiral HPLC. Diastereomeric splitting in high precision NMR measurements is one approach for experimentally probing PV effects with racemic samples and the favorable NMR properties of the ^77^Se nucleus further increase the interest in the above-mentioned targeted model compounds.^32^

**Figure 1:**
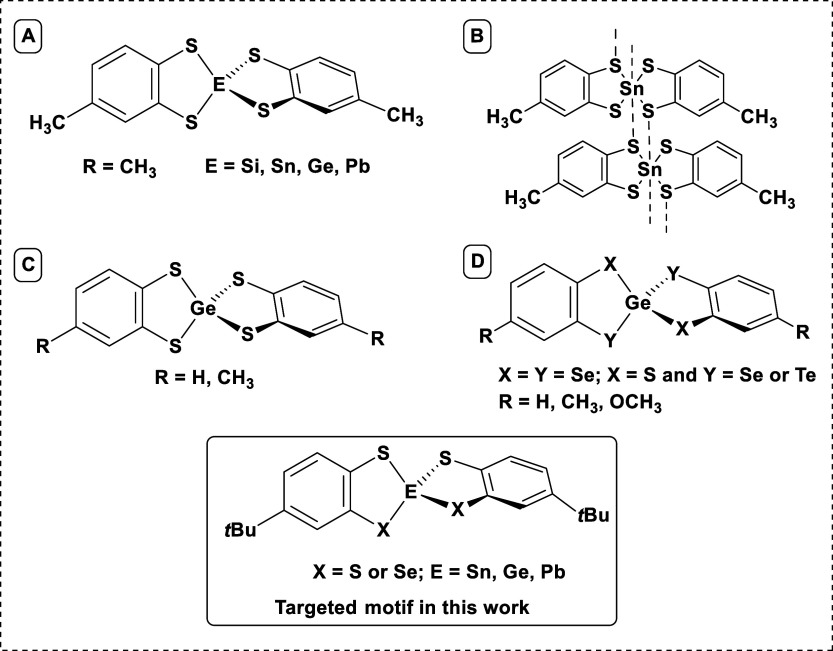
Examples of reported E­(IV) (A,^21^ B,^25^ C,^18^ D^14,30^ ) compounds with different 1,2- benzene-bischalcogenolate or 3,4-toluene-bischalcogenolate ligands and the targeted motif for this work.

## Results and Discussion

As an initial approach to exploring axially chiral spiro compounds featuring group 14 elements, we functionalized the *ortho* position of the thiol group in compound **1** with a selenol (−SeH) or an additional thiol (−SH) group, employing procedures developed in previously established research (Scheme 1).^33,34^ Compound **2** has been previously reported in the literature, and its spectral characteristics are consistent with the findings from earlier studies.^33^ By contrast, compound **3** had not been reported but can be obtained in a similar fashion albeit in low yield (25%) with a purity of 97%.

**Scheme 1:**
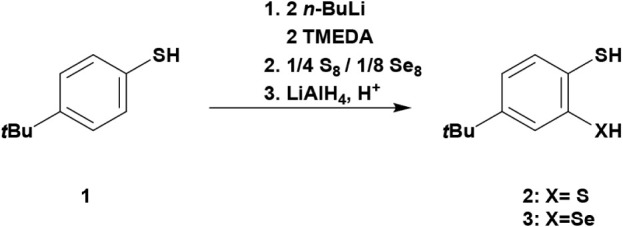
Synthesis of Starting Materials **2** and **3**

The ^1^H NMR spectra of compound **3** show a resonance corresponding to the proton of the −SH group at 3.75 ppm, while the resonance of the −SeH proton is observed at 1.86 ppm. The latter signal shows well resolved selenium satellites for which the value of the coupling constant (^1^ *J*_SeH_ = 53.6 Hz) is consistent with the value reported for its analogue C_6_H_4_(SH)­(SeH) without *tert*-butyl group (^1^ *J*_SeH_ = 55 Hz).^34^ Furthermore, the ^77^Se­{^1^H} NMR resonance of **3** at 141.8 ppm is close to the one reported for compound C_6_H_4_(SH)­(SeH) (*δ* = 134.8 ppm).^34^ The composition of compound **3** was further confirmed by ESI-HRMS measurement in negative mode and through elemental analysis.

The full deprotonation of compounds **2** and **3** afforded the corresponding compounds **2a** and **3a** as colorless solids (Scheme 2). These compounds form in pentane solution when treated with two equivalents of *n*-BuLi. The ^1^H NMR spectra confirmed the formation of compounds **2a** and **3a**. Additionally, ^7^Li NMR spectroscopy revealed resonances for **2a** at 3.3 ppm and for **3a** at 1.5 ppm.

**Scheme 2:**
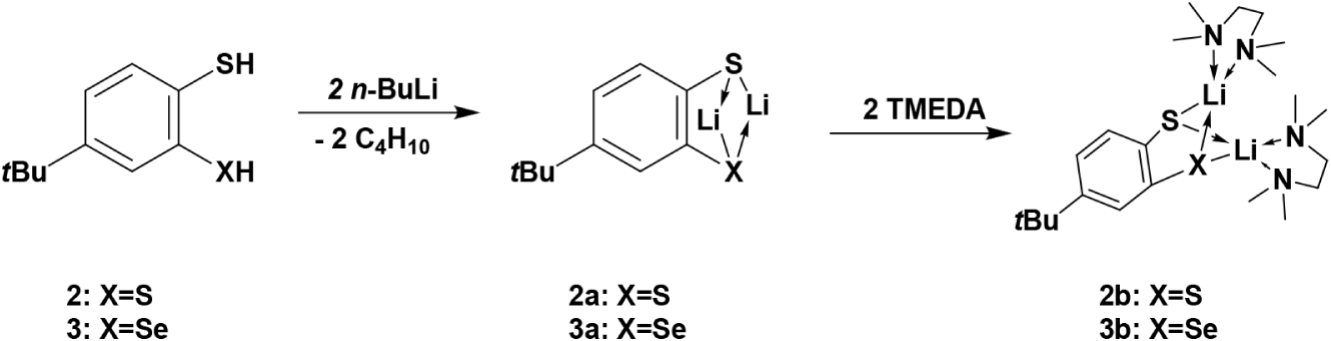
Synthetic Access to the Lithiated Derivatives of Compounds **2** and **3**

To gain more insight into the lithium coordination, the crystallization of products **2a** and **3a** was achieved in the presence of two equivalents of TMEDA while maintaining the diethyl ether solution at −33 °C. The molecular structures in the solid state, as well as the selected bond lengths and angles of compounds **2b** and **3b** are depicted in Figure 2. The S–Li bond distances in compound **2b** (S2–Li2 2.378(4) Å, S2–Li1 2.402(4) Å, S1–Li1 2.387(4) Å, S1–Li2 2.389(3) Å), are comparable to those of the S–Li bond in compound **3b** (S1–Li1 2.351(6) Å), but considerably shorter than previously reported values ([C_6_H_5_S­((CH_3_)_3_Si)­CHLi·2THF]_2_: Li–S1:2.586(3) Å^35^ ((CH_3_)_3_Si)_2_C­(C_6_H_5_)­SLi · 3THF: 2.476(5) Å^36^). The Se1–Li1 (2.550(5) Å) bond length in compound **3b** is consistent with values found in the literature ([{Li­(bipy)­(SePh)}_2_]: Se1–Li1:2.552(9) Å, Se1–Li2:2.588(10) Å).^37^ The bond angles around the sulfur atoms in compounds **2b** and **3b** are similar. The C1–Se1–Li1 (79.47(15)°) and C1–Se1–Li2 (76.02(16)°) bond angles in compound **3b** are smaller than the values documented in the literature (([{Li­(bipy)­(SePh)}_2_]: C(13)–Se(1)–Li, C(13)–Se(1)–Li’: 99.5(3)°).^37^ Conversely, the Li1–Se1–Li2 bond angle (78.77(19)°) is slightly larger than the literature value for the corresponding angle (([{Li­(bipy)­(SePh)}_2_]: Li–Se­(1)-Li’: 75.8(4)°).^37^

**Figure 2:**
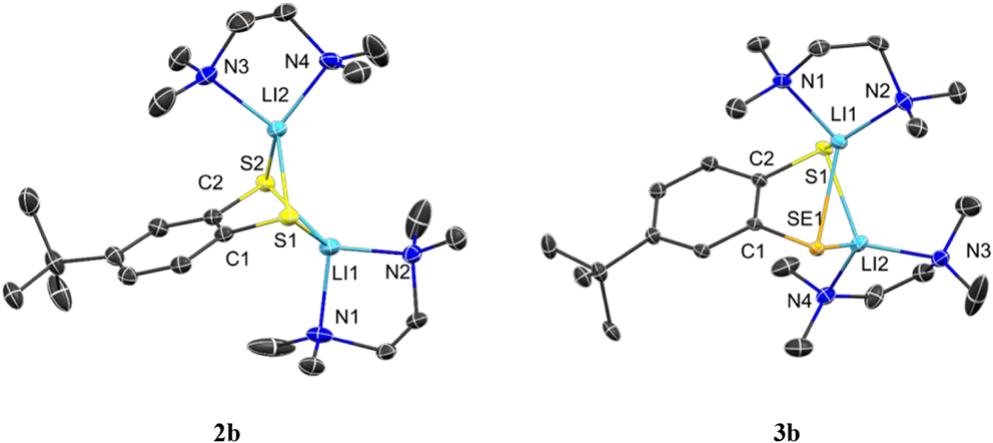
Molecular structures of **2b** and **3b** in the solid state. Ellipsoids are shown at 30% probability. All hydrogen atoms are omitted for clarity. Selected bond lengths [Å] and angles [°] in **2b**: C2–S2 1.775(2), C2–C1 1.423(3), C1–S1 1.773(2), S2–Li2 2.378(4), S2–Li1 2.402(4), S1–Li1 2.387(4), S1–Li2 2.389(3); C1–C2–S2 122.87(15), C2–S2–Li1 82.58(11), C2–S2–Li2 80.17(11), Li1–S2–Li2 81.64(12), C2–C1–S1 123.14(15), C1–S1–Li2 81.42(11), C1–S1–Li1 81.30(11), Li1–S1–Li2 81.73(12). **3b**: C1–C2 1.407(4), C1–Se1 1.889(3), Se1–Li1 2.550(5), Se1–Li2 2.499(6), C2–Li1 2.764(6), S1–Li1 2.351(6), S1–Li2 2.364(6); C1–C2–S1 124.2(2), C2–S1–Li1 83.51(17), C2–S1–Li2 82.44(18), Li1–S1–Li2 85.6(2), C2–C1–Se1 123.3(2), C1–Se1–Li1 79.47(15), C1–Se1–Li2 76.02(16), Li1–Se1–Li2 78.77(19).

With the protonated (**2** and **3**) and deprotonated (**2b** and **3b**) starting materials at hand, we set out to prepare axially chiral spiro compounds incorporating a group of 14 element, such as Sn, Ge, and Pb in the spiro-center. The syntheses were carried out using the corresponding element tetrachlorides in case of Ge and Sn (Scheme 3), while Pb (IV) acetate was used as the Pb­(IV) precursor (Scheme 3). In the first approach, we obtained the desired Ge and Sn compounds through a salt metathesis reaction. Identical results were observed by refluxing compounds **2** and **3** with ECl_4_ (E = Ge, Sn) in toluene.

**Scheme 3:**
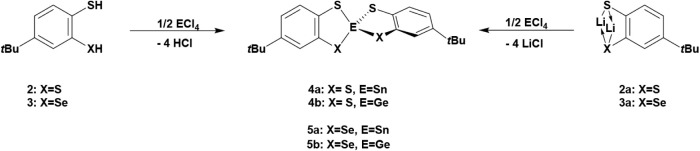
Synthesis of Axially Chiral Compounds with Sn or Ge as Spiro Center

Tin compound **4a** is characterized by its bright red color and reveals insolubility in nonpolar solvents at ambient temperature. However, heating compound **4a** in toluene at reflux leads to complete dissolution and affords a clear yellow solution. Upon cooling, the solution changes to red, and the compound precipitates. Moreover, **4a** is soluble in tetrahydrofuran (THF) forming a yellow solution. The ^119^Sn­{^1^H} NMR resonance of **4a** in THF-*d*_8_ solution appears as a singlet at −77.3 ppm. APCI-DIP-HRMS confirms the identity of **4a**, displaying a peak corresponding to the protonated molecule [M + H]^+^. The purity of compound **4a** was further verified by elemental analysis. Formally swapping two sulfur atoms in **4a** to two selenium atoms refers to compound **5a** which is a yellow solid that shows increased solubility compared with its sulfur counterpart **4a**. Compounds **4a** and **5a** exhibit remarkable air stability in solid state, showing no signs of decomposition over several days. Compound **5a** is soluble in common organic solvents, forming a yellow solution and yielding a yellow solid upon drying. By contrast, evaporation from DCM affords a violet solid (compound **5a** was tested both, in dry DCM under inert conditions, and nondried DCM under ambient conditions, without notable difference). Subsequent drying of the violet product under reduced pressure, followed by dissolution in THF-*d*_8_ or DCM-*d*_2_, yields a yellow solution. Interestingly, the spectral data obtained from this yellow solution is consistent with that of the previously acquired yellow solid. The IR spectra of both solids (yellow and violet) were compared (cf. SI file). No new bands were observed, but several vibrational bands are shifted up to 15 cm^–1^ and relative intensities vary, which could possibly be attributed to differences in molecular packing consistent with polymorphism.^38–40^ It should be noted, that similar color changes have been reported in distannene and cyclotristannane systems, where they are associated with equilibrium shifts stabilized by London dispersion interactions.^41^ The ^119^Sn­{^1^H} NMR resonance of **5a** appears as a singlet at −31.8 ppm (in THF-*d*_8_), which is deshielded compared to compound **4a**. When the coordinating solvent, THF-*d*_8_, is replaced with a nonpolar solvent, such as toluene-*d*_8_ or cyclohexane-*d*_12_, the ^119^Sn­{^1^H} NMR resonance of **5a** shifts from upfield to downfield (toluene-*d*_8_: 92.8 ppm, cyclohexane-*d*_12_: 98.0 ppm) indicating a substantial change in the tin coordination environment. The ^119^Sn­{^1^H} NMR spectrum of compound **5a** is accompanied by low-intensity satellites with a value of the coupling constant ^1^ *J*_SnSe_ = 1553 Hz, which is consistent with reported values in the literature.^42,43^ The complementary ^77^Se NMR spectrum displays a singlet at 210.3 ppm with a constituent satellite coupling. The identity and purity of compound **5a** were further verified by APCI-DIP-HRMS, which displayed a peak corresponding to [M + H]^+^ and by elemental analysis. Despite multiple attempts, single crystals of tin compounds **4a** and **5a** could not be obtained, both compounds were obtained as powders.

The analogous Ge­(IV) compounds **4b** and **5b** are colorless solids that dissolve in nonpolar solvents such as benzene and toluene at ambient temperature. The ^77^Se­{^1^H} NMR resonance for compound **5b** appears at 266.6 ppm, which is quite close to the literature-reported spirobisphenyleneheterodichalcogenagermole (*δ* = 263.8 ppm).^14^ The composition of **4b** and **5b** has been confirmed via APCI-DIP-HRMS, with *m*/*z* corresponding to [M + H]^+^, and the purity of both compounds is corroborated by elemental analysis. Compounds **4b** and **5b** exhibit remarkable air stability in the solid state, showing no signs of decomposition after several days in air. Furthermore, compounds **4b** and **5b** are thermally robust and can be sublimed without detectable decomposition (**4b**: 7.2 × 10^–2^ mbar at 178 °C; **5b**: 7.2 × 10^–2^ mbar at 203 °C). Single crystals suitable for X-ray diffraction analysis were isolated from a toluene solution after refluxing and gradually cooling. The molecular structures with selected bond lengths and angles of compounds **4b** and **5b** are depicted in Figure 3.

**Figure 3:**
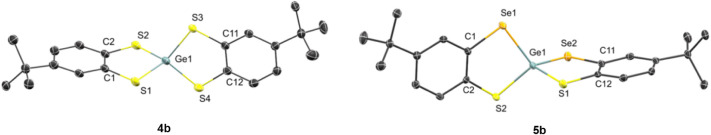
Molecular structure of spiro chiral Ge­(IV) compounds **4b** and **5b**. Ellipsoids are shown at 30% probability. All hydrogen atoms are omitted for clarity. Selected bond lengths [Å] and angles [°] in **4b**: C1–S1 1.771(4), C2–S2 1.770(4), C11–S3 1.772(4), C12–S4 1.763(5), S1–Ge1 2.1984(14), S2–Ge1 2.2044(12), S3–Ge1 2.2032(12), S4–Ge1 2.1955(13); S1–Ge1–S2 98.20(5), S1–Ge1–S4 116.17(5), S2–Ge1–S3 115.41(4), S3–Ge1–S4 98.52(4), S2–Ge1–S4 113.00(5), S1–Ge1–S3 116.54(5), C1–S1–Ge1 97.09(15), C2–S2–Ge1 96.94(15), C11–S3–Ge1 97.35(14), C12–S4–Ge1 97.06(15); in **5b**: C1–Se1 1.929(6), C12–S1 1.763(6), C11–Se2 1.938(6), C2–S2 1.765(6), S1–Ge1 2.2042(17), S2–Ge1 2.2166(16), Se1–Ge1 2.3326(9), Se2–Ge1 2.3115(9); Se1–Ge1–S1 114.59(5), Se2–Ge1–S2 114.88(5), Se1–Ge1–S2 97.47(5), S1–Ge1–Se2 99.07(5), S1–Ge1–S2 113.71(7), Se2–Ge1–Se1 118.10(4), C1–Se1–Ge1 92.40(18), C12–S1–Ge1 98.7(2), C11–Se2–Ge1 93.06(18), C2–S2–Ge1 98.7(2).

The four C–S bond lengths in compound **4b** are all within a similar range. The C2–S2(1.765(6) Å) and C12–S2(1.763(6) Å) bond lengths in compound **5b** are similar to those in compounds **4b**, and the related phenylene derivative (spirobisphenylenedithiagermole: 1.772(3) Å) reported before.^18^ Both C–Se bond distances in compound **5b** are quite close with 1.929(6) Å (C1–Se1) and 1.938(6) Å (C11–Se2). Similarly, the S–Ge bond lengths in molecular structures **4b** and **5b** differ only marginally from one another, fully in line with S–Ge bond lengths reported in the literature.^18^ The Se2–Ge1 bond length is slightly shorter than the Se1–Ge1 in compound **5b**. The S2–Ge1–S1 and S3–Ge1–S4 bond angles in compound **4b** are around 98°, while the S1–Ge1–S4 and S2–Ge1–S3 bond angles are around 116°. These values align well with those findings reported for the compound (C_6_H_4_S_2_)_2_Ge^18^ in the literature. Similarly, the S2–Ge1–Se1 and S1–Ge1–Se2 bond angles for compound **5b** are around 97–99°, while the S2–Ge–S1 and Se1–Ge1–Se2 bond angles are around 115°. To better understand the coordination environment around the Ge atom in both compounds **4b** and **5b**, the τ_4_ parameter was calculated according to the literature.^44^ The calculated τ_4_ value for each compound is 0.90, indicating nearly ideal tetrahedral geometry, with only minor distortion. The minor deviation from the ideal tetrahedral angle could arise from the angular strain imposed by the chelating ligands.

Approaching the analogous Pb­(IV) compounds, Pb­(IV) acetate was selected as lead source which has been successfully employed according to related works.^21,45^ Since the lead source is a strongly oxidizing reagent and ligands **2** and **3** are sensitive to oxidation,^46,47^ the reaction was carried out at a low temperature and in the absence of light, following the procedure described for diorganosubstituted analogs in the literature.^45^ Reacting Pb­(OAc)_4_ with compound **2** yielded a yellow solid (Scheme 4). When the product was dissolved in pyridine-d_5_, yellow needles precipitated from the solution. The molecular structure of this product indicates a change in oxidation state from Pb­(IV) to Pb­(II) (Figure 5). In addition, analysis of the crude reaction product with HRMS reveals a peak consistent with the protonated molecular ion [M + H]^+^ of **8** (1,2,5,6 tetrathiocin) (cf. SI file) as a side-product, which supports the decomposition pathway of the Pb­(IV) products.^48–50^ Owing to their low solubility and sensitivity, the previously reported spirobistoluenedithiaplumboles have not been fully characterized^21,45^ lacking firm evidence regarding the oxidation state of lead in these cases. To independently confirm the identity of the Pb­(II) product **6a**, a control reaction was performed with the Pb­(II) source Pb­(N­(TMS)_2_)_2_ in a 1:1 ratio. The isolated product **6a** displayed NMR spectral features consistent with those observed in the reaction to **6** with the Pb­(IV) precursor. The ^207^Pb NMR spectra feature a resonance at 2784 ppm, which is consistent with the resonance known for [Pb­(SPh)_3_]^−^ in the literature (2808 ppm).^51^ Elemental analysis verifies the composition of product **6a**. The protonated molecular ion [M + H]^+^ and that of its dimer [2M + H]^+^ have been obtained in the gas phase via HRMS using direct injection APCI-DIP. To assess whether such aggregation occurs in solution as well, diffusion-ordered NMR spectroscopy (DOSY) was performed for **6a** using DMSO-*d*_6_ and TMS as a reference. The DOSY NMR spectra were interpreted using the molecular weight estimation program developed by Stalke et al.,^52–55^ confirming that compound **6a** is monomeric in solution (cf. SI file).

**Scheme 4:**
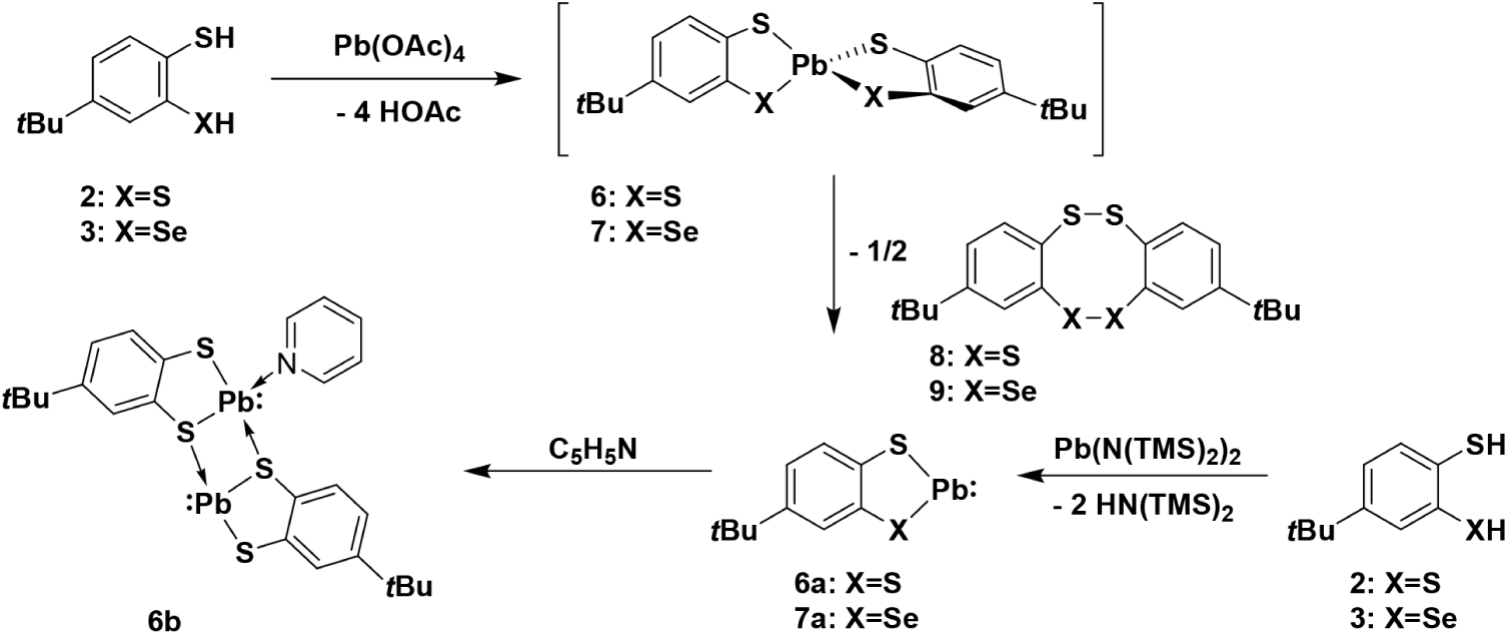
Synthesis of the Pb Compounds with Ligand **2** and **3**

When compound **6a** is crystallized from pyridine its pyridine adduct **6b** is obtained as yellow needles. Elemental analysis of the crystalline material is consistent with the presence of one coordinated pyridine ligand per formula unit. The molecular structure with selected bond lengths and angles of compound **6b** are depicted in Figure 5. Furthermore, the molecular structure reveals that compound **6b** adopts a polymeric arrangement in the solid state, in which the individual molecules engage in mutual intermolecular coordination. Thus, the asymmetric unit of **6b** contains two lead atoms, Pb1 bonded to atoms S1, S3, and N1, and Pb2 atom, bonded to atoms S2 and S4. Additionally, Pb2 is connected to S3 with a distance of 2.7975(16) Å, while Pb1 exhibits an intermolecular interaction with S4 at 3.566(2) Å. The remaining components generated by symmetry operations show that S2’ and S4’ are connected to Pb1 through intermolecular interactions at distances of 3.027(1) Å and 3.234(2) Å. Moreover, S1’ and S4’’ are connected through intermolecular interactions to Pb2 at distances of 3.121(2) Å and 3.090(1) Å. These interactions lead to the formation of a linear polymer that propagates along the *b* axis. The measured intramolecular Pb–S bond lengths within the studied structure shows remarkable agreement with previously literature-reported values Pb_3_(SC_6_H_4_S)_3_(en)_2_.^56^ Furthermore, the Pb1•••Pb2 4.220(4) Å and Pb2•••Pb1’ 4.056 (4) Å distances are shorter than the sum of the van der Waals radii (4.68 Å),^57^ however, significant intermolecular Pb•••Pb interactions are unlikely considering comparable examples discussed in the literature^58^ (Figure 4).

**Figure 4:**
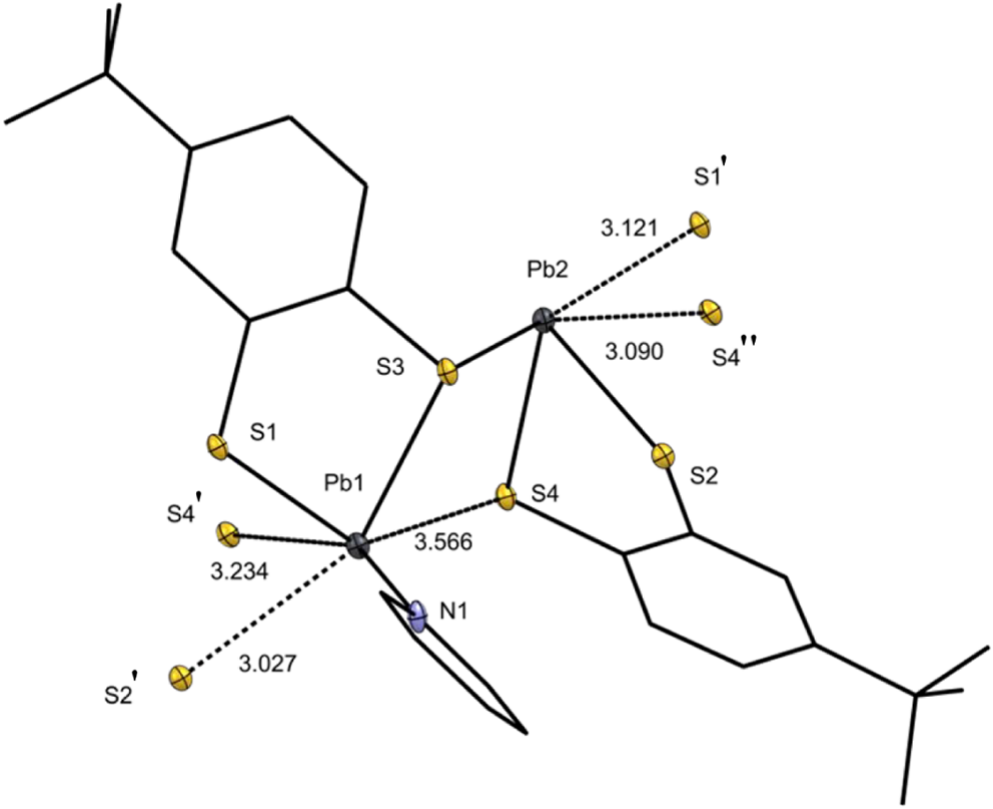
Molecular structure of Pb­(II) compound **6b**. Ellipsoids are shown at 30% probability. All hydrogen atoms and an uncoordinated pyridine solvent molecule are omitted. For clarity, only the heteroatoms are shown with ellipsoids, while the other atoms are represented in wireframe form. Selected bond lengths [Å] and angles [°] in **6b**: C1–S1 1.764(7), C2–S3 1.763(7), C11–S2 1.769(7), C12–S4 1.775(7), S1–Pb1 2.6575(18), S2–Pb1 3.0272(16), S3–Pb1 2.7897(17), S3–Pb2 2.7975(16), S4–Pb2 2.8060(17), S2–Pb2 2.6752(16); S1–Pb1–S3 74.53(6), S1–Pb1–N1 89.17(12), S3–Pb1–N1 74.53(6), S3–Pb2–S2 76.29(5), S2–Pb2–S4 74.36(5), S3–Pb2–S4 97.11(5).

To further extend the scope, compound **3** was reacted with Pb­(OAc)_4_, which yielded a bright orange solid. The latter is soluble in pyridine-d_5_ and ^77^Se NMR spectroscopy indicates consumption of the starting material. Furthermore, the MS-APCI from the crude product confirms formation of the Pb­(II) compound **7a** and **9** ([1,5,2,6] dithiadiselenocin). Considering a potential reduction of Pb­(IV) by the ligand as in the case of the dithiol ligand, a control reaction starting from Pb­(N­(TMS)_2_)_2_ and **3** in a 1:1 ratio was performed. Full characterization indicates that the product was indeed the Pb­(II) compound **7a** regardless of the oxidation state of lead in the starting material. The ^77^Se NMR of **7a** shows a resonance at 404.1 ppm. The ^207^Pb NMR spectra of **7a** show a resonance at 2971 ppm, consistent with the formation of the Pb­(II) product, and close to the resonance reported for [Pb­(SePh)_2_SPh]^−^ in the literature.^51^ The identity of **7a** is further supported by HRMS and its purity by elemental analysis. The protonated molecular ion [M + H]^+^ obtained via APCI-DIP HRMS using direct injection APCI, indicates **7a** as a monomer, besides dimeric [2M + H]^+^. By contrast to **6a,** attempts to crystallize compound **7a** were unsuccessful.

In an attempt to obtain derivatives of **7** and **7a** suitable for structural characterization, we added the NHC, Im4Me, to a solution of isolated **7a**, as well as to the *in situ* prepared **7**. The reaction of isolated product **7a** with Im4Me leads to formation of the targeted adduct **7b** (Scheme 5). Coordination of the Im4Me to Pb center significantly increases solubility of the plumbylene, enabling dissolution in nonpolar solvent such as toluene. The proton NMR spectra of **7b** resemble those of compound **7a** with two additional resonances attributable to Im4Me group. The ^77^Se NMR spectrum of **7a** shows a resonance at 252 ppm, which is upfield shifted compared to **7b**. The ^207^Pb NMR spectrum of compound **7b** displays a resonance at 2070 ppm, which is shielded compared with compound **7a.** This upfield shift is consistent but less pronounced compared with the one observed for a related plumbylene adding the same Im4Me donor ligand.^59^

**Scheme 5:**
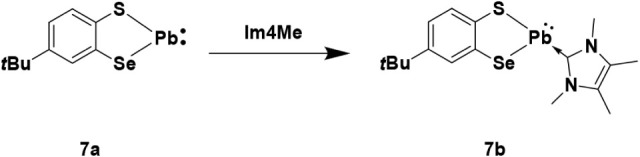
Reaction of 7a with Im4Me

Single crystals of **7b** for X-ray diffraction were obtained by slow diffusion of pentane into a dichloromethane solution, however structural disorder led to reduced precision of the C–C bond length. The structure solution of **7b** clearly establishes the coordination of Im4Me to the lead as depicted in Figure 5.

**Figure 5:**
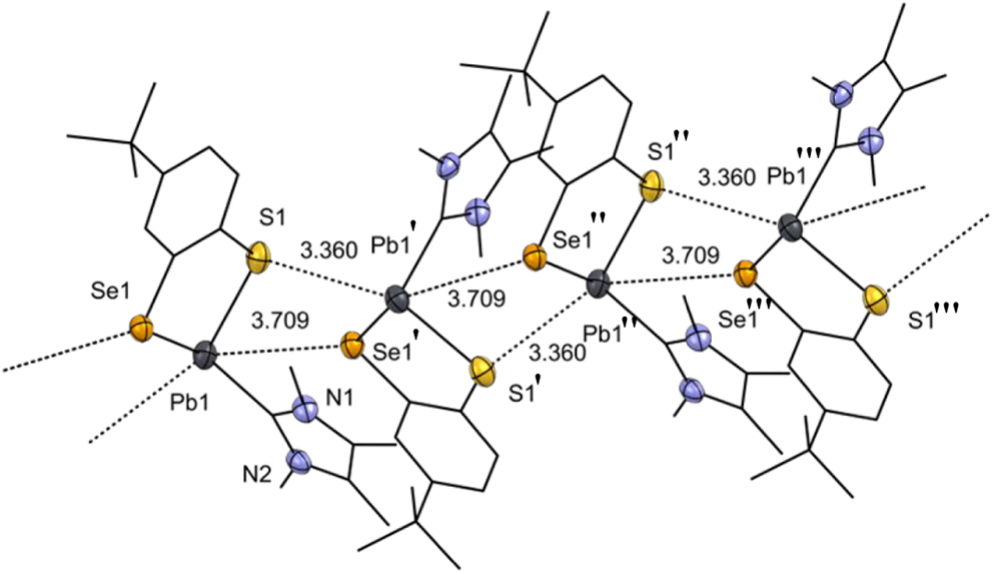
Molecular structure of compound 7b. Ellipsoids are shown at 30% probability. All hydrogen atoms are omitted for clarity. For clarity, only the heteroatoms are shown with ellipsoids, while the other atoms are represented in wireframe form. A discussion of the bond lengths is limited by low C–C bond precision, owing to disorder and low data quality, and therefore omitted.

The molecular structure shows that the *t*Bu­(C_6_H_3_)­SSe group is disordered over two positions with refined occupancies of 0.75 and 0.25. Furthermore, the crystal packing shows that the polymeric structure of **7b** in the solid state is retained, contrasting the monomerization of aggregated plumbylenes with Im4Me in related examples reported before.^59^ The solid state structure of **7b** indicates a linear polymeric framework formed via intermolecular Pb•••S (3.360(2) Å) and Pb•••Se (3.709(7) Å) interactions, which propagates along the crystallographic *b* axis. Furthermore, the Pb•••Pb distance 4.709(1) Å is at the borderline of the sum of the van-der-Waals radii (4.68 Å)^57^ and likely is a result of the crystal packing.

The addition of NHC to the product mixture obtained via the Pb­(IV) precursor led to a mixture of three different species (Scheme 6) which could be crystallized and were suitable for single crystal X-ray diffraction, whereas other characterization methods could not be applied owing to the limited amount and failed separation of the individual components of the mixture. Interestingly, the NHC does not act simply as a donor but leads to more complex reactions again involving redox chemistry also involving the ligand. By contrast, the addition of the NHC to **7a** obtained directly from the Pb­(II) precursor avoids reaction of the NHC with oxidation products of ligand **3** present in the mixture obtained from the Pb­(IV) precursor.

**Scheme 6:**
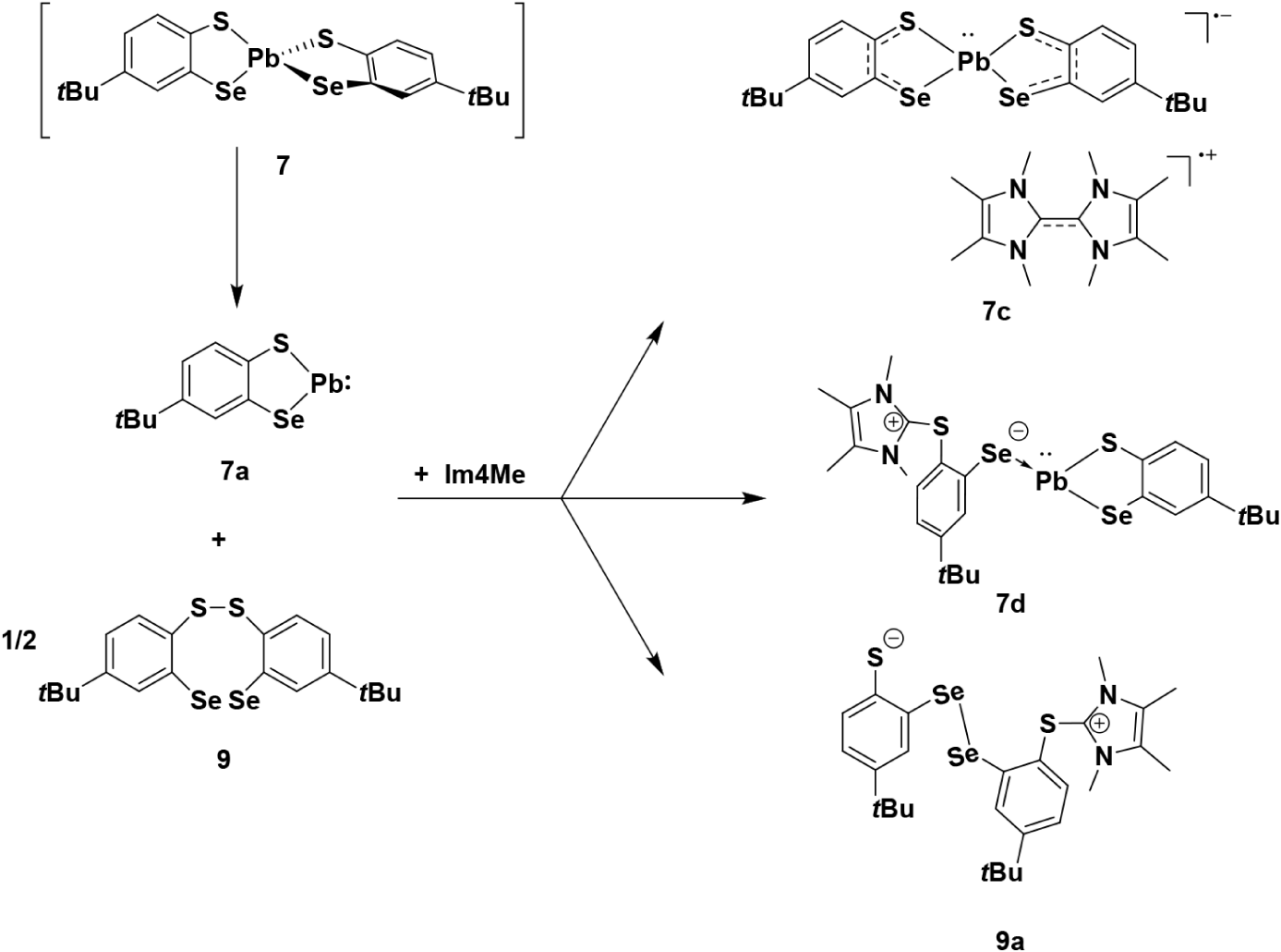
Products of the Reaction of Im4Me with 7. Due to the Formation of an Inseparable Mixture of Three Products (**7c**, **7d**, and **9a**) the Reaction Scheme is Presented in a Simplified form Without Precise Stoichiometric Balance

The molecular structures with selected bond lengths and angles of **7c**, **7d**, and **9a** are given in Figure 6. The geometry around the lead atom in **7c** deviates from the typical tetrahedral coordination as observed in related germanium complexes (**4b** and **5b**) indicating a stereochemically active lone-pair at lead in **7c**. The observed bond angles are consistent with a highly distorted geometry around a Pb­(II) center. Additionally, Im4Me forms a dimer instead of coordinating to the Pb center. The molecular structure shows that one of the NHC units is twisted out of plane. The bond length between C1 and C28 in the Im4Me dimer is 1.44(18) Å, which is similar to the value found in the previously reported NHC-CAAC heterodimer in form of its radical monocation (1.439(3) Å) but shorter than the corresponding dication (1.491(4) Å).^60,61^ In general, these values are significantly elongated compared with a typical C=C double bond but shorter than a C–C single bond.^62^ The average torsion angle between the two Im4Me is 60.6° and the geometry around the nitrogen atoms of both Im4Me is planar [∑(CNC)­av: 359.92°] which fits well with the literature-reported radical cationic NHC-CAAC heterodimer (torsion angle: 56.5° and ∑(CNC)_av_: 358.53°).^60^ This structural information suggests that the Im4Me ligand underwent single electron oxidation to form the radical cation. This behavior could be facilitated due to the noninnocent character of the bischalcogenolato ligands, which is a hallmark of noninnocent behavior in redox chemistry.^17,63,64^

**Figure 6:**
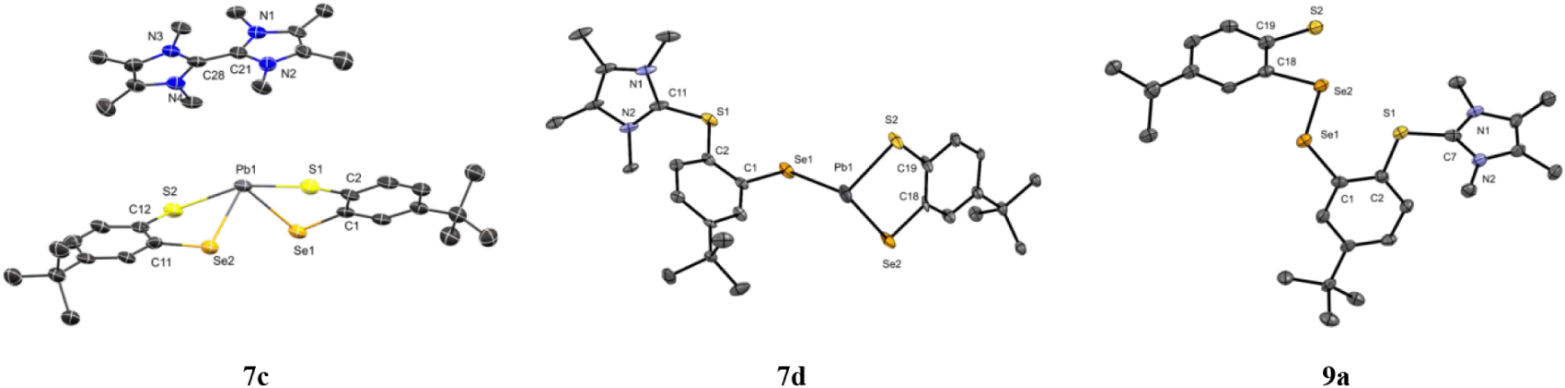
Molecular structure of Pb­(II) compounds **7c, 7d,** and **9a**. Ellipsoids are shown at 30% probability. All hydrogen atoms are omitted for clarity. The compound **7d** crystallizes as a centrosymmetric dimer, but only one-half of the molecule is shown for clarity. Selected bond lengths [Å] and angles [°] in **7c**: C1–Se1 1.893(13), C2–S1 1.743(14), C11–Se2 1.918(11), C12–S2 1.785(13), S1–Pb1 2.824(4), S2–Pb1 2.938(3), Se2–Pb1 2.7375(14), Se1–Pb1 2.7618(15); S1–Pb1–Se1 76.58(7), S2–Pb1–Se2 75.83(7), Se2–Pb1–Se1 96.47(4), S1–Pb1–S2 162.82(9); in **7d**: C1–Se1 1.885(18), C2–S1 1.82(2), C18–Se2 1.913(18), C19–S2 1.770(18), C11–S1 1.77(3), Se1–Pb1 2.911(3), Se2–Pb1 2.691(2), S2–Pb1 2.593(6); S2–Pb1–Se2 82.44(13), Se1–Pb1–S2 93.43(19), Se2–Pb1–Se1 96.18(8); in **9a:** S1–Se2 2.508(2), C1–Se1 1.926(13), C2–S1 1.772(12), S1–C7 1.730(15), C18–Se2 1.903(13), C19–S2 1.738(14); C2–S1–C7 102.5(6), S1–C7–N1 127.3(10), S1–C7–N2 124.8(10).

Compound **7d** exhibits a centrosymmetric dimeric arrangement in the solid state, for clarity, only one symmetry-independent half of the molecule is shown in Figure 6. The Pb•••Se intermolecular contact (3.544(3) Å) is shorter than the sum of the corresponding van der Waals radii (4.52 Å),^57^ indicating intermolecular interactions. The molecular structure of **9a** indicates that Im4Me cleaves the homocoupled S–S bond in **9** leading to the formation of zwitterionic species of **9a**. Moreover, an additional polymeric structural motif was obtained **(7e)** combining structural subunits of the arrangements in compounds **7a** and **7d**. Details of this molecular structure of **7e** with bond lengths and interatomic distances are available in the SI (Figure S54).

Similar to previous examples in the literature,^30^ the spirobistoluenediselenagermoles and spirobismethoxybenzodiselenagermoles, although consisting of chiral molecules, were isolated and characterized as racemic mixtures. We set out to explore the possibility of chiral resolution using analytical chiral HPLC. Based on their favorable solubility, spiro germanium compounds **4b** and **5b** were selected for this purpose, assuming the *tert*-butyl groups at the aryl rings would allow differences in the interaction with the stationary phase sufficient for chiral resolution.

To this end, the chromatographic separation of target compounds was performed using a *Chiral Pak IJ* as chiral stationary phase (CSP), which had already been employed as the stationary phase for spiro chiral compounds in the literature.^65^ *Chiral Pak IJ* consists of a cellulose ester (cellulose tris­(4-methylbenzoate), with all hydroxyl groups fully esterified. The choice of the mobile phases was determined based on solubility and UV–vis absorption properties of the target compounds (**4b**: λ_abs_ = 232 nm**; 5b**: λ_abs_= 237 nm). All HPLC experiments were performed at room temperature with four different wavelength channels (237 nm; 254 nm; 280 nm; 290 nm). Unlike compound **5b**, we could not identify any conditions with peak separation for compound **4b**. By contrast, the HPLC chromatogram of **5b** (cf. SI file) shows partial separation of two overlapping peaks. To resolve the latter, curve deconvolution was performed using Python indicating that the analytical HPLC separation of compound **5b** furnishes two components in a 60:40 ratio. Since the ratio deviates significantly from the expected 1:1 ratio of enantiomers from a racemate, the occurrence of a second peak in the chromatogram may likely originate from partial decomposition of compound **5b** rather than from chiral resolution. In essence, the selected chiral column did not provide adequate separation, alternative stationary phases will be tested in future work to validate whether the nonequimolar ratio relates to an analytical artifact.

## Conclusion

A set of 4-*tert*-butyl-benzene-bischalcogenol ligands, **2** and **3**, with dithiol or combined thiol and selenol groups has been prepared and characterized along with their deprotonated derivatives **2**(**a**,**b**), **3**(**a**,**b**). With these ligands in hand, we set out preparing *spiro*-complexes with a tetravalent group 14 element *spiro*-center in analogy to previously reported examples.^14,21,26,30^ While these latter examples carry only small substituents, ligands **2** and **3**, carry a bulky *tert*-butyl group in *para*-position of one thiol group. This substituent gives rise to axial chirality in case of the dithiolate based *spiro*-compounds, whereas the mixed thiolate selenolate *spiro*-compounds are intrinsically chiral. With these versatile precursors in hand, we investigated the above-mentioned axially chiral compounds, incorporating group 14 element Ge, Sn, and Pb. All Ge and Sn complexes have been characterized using multinuclear NMR spectroscopy, elemental analysis (EA), and high-resolution mass spectrometry (HR-MS) in addition to single crystal X-ray diffraction for germanium compounds **4b** and **5b**. The germanium compounds **4b** and **5b** are thermally quite robust and can be sublimed without signs of decomposition. Nevertheless, our attempts to perform chiral resolution via HPLC on the column *Chiral Pak IJ* was unsuccessful and peak splitting is most likely a consequence of partial decomposition of **5b** under HPLC conditions. Nonetheless, racemic **5b** will be of interest for further investigation with respect to PV effects in future work.^32^

In contrast to previous reports,^21,45^ the reaction of precursors **2** and **3** with Pb­(IV) sources involves redox chemistry resulting in the corresponding plumbylenes **6a** and **7a** and their derivatives. The reduction of Pb­(IV) to Pb­(II) involves oxidation of the ligands to 1,2,5,6 tetrachalcogenocins **8** and **9** as byproducts. The addition of donor molecules (pyridine, NHC) does not lead to deaggregation of the polymeric plumbylenes. Likewise, the NHC can add to **8** or **9** and the resulting chalcogenolates act as a donor to the plumbylenes as well, giving rise to a variety of complex adducts.

## Experimental Section

### General Considerations

All manipulations related to the syntheses were carried out under an argon atmosphere by means of standard Schlenk or glovebox techniques. Solvents (toluene, *n*-hexane, *n*-pentane, THF, Et_2_O) were dried over Na/K alloy before use and were freshly distilled under an inert gas. Anhydrous dichloromethane (DCM) was dried using a solvent purification system (SPS) and stored over 3 Å molecular sieves under inert conditions. NMR solvents (purchased from Deutero) were dried and stored over 3 Å molecular sieves under an argon atmosphere. Reagents and chemicals were purchased from commercial suppliers (Sigma-Aldrich, ABCR) and used as received. The ligand 4-*tert*-butyl-1,2-benzenedithiol was synthesized according to the literature.^33^ The synthesized **4**(**a**, **b**) and **5**(**a**, **b**) were observed to be air-stable and could be handled under ambient conditions.

All solution-phase NMR spectra were recorded on Jeol JNM-ECZL500 or Varian VNMRS-500 MHz spectrometers at 25 °C. Chemical shifts were referenced to residual protic impurities in the solvent (^1^H) or the deuterated solvent (^13^C) and reported relative to external SiMe_4_ (^1^H, ^13^C), Me_2_Se (for ^77^Se), SnMe_4_ (for ^119^Sn), or PbMe_4_ (for ^207^Pb). ESI-HR or APCI-DIP-HR mass measurements were performed on a Finnigan LCQ Deca (ThermoQuest). Elemental analyses were performed with a HEKAtech Euro EA CHNS elemental analyzer. Samples were prepared in a tin cup with the addition of V_2_O_5_ to promote the combustion. Absorption spectra were recorded using a Shimadzu UV-1900 spectrometer. HPLC was run by a Knauer HPLC using a DAICEL’s *Chiral Pak IJ* cellulose tris­(4-methylbenzoate) (250 mm × 4,6 mm Particle: 5 μm). IR measurements were performed with a Bruker Alpha Diamond-ATR spectrometer.

Crystallographic measurements were carried out on a Stoe IPDS2 or a Stoe StadiVari diffractometer with a STOE image plate detector and a Mo–Kα (λ = 0.71073 Å) monochromator or a Stoe StadiVari diffractometer with a Pilatus 200 K image plate detector and Cu–Kα (λ = 1.54186 Å) radiation. Direct methods were used to solve the measurements and refined by “least-square” cycles (SHELXL-2017).^66^ The evaluation of the data sets, as well as the graphical preparation of the structures was performed using Olex2 ^67^ and Mercury.^68^ Details of the structure determinations and refinement are summarized in Tables S2–S6 (cf. SI file). The CCDC depositions 2526565–2526574 contain the supplementary crystallographic data for this paper, which can be obtained free of charge via emailing data_request@ccdc.cam.ac.uk, or by contacting The Cambridge Crystallographic Data Centre at 12 Union Road, Cambridge CB2 1EZ, UK; fax: + 44 1223 336033.

### Synthetic Protocols and Characterization

#### Synthesis of Compound 2

Compound **2** was prepared according to Block et al.^33^

^**1**^ **H NMR (500 MHz, C**_**6**_ **D**_**6**_ **):** δ 7.27 (d, *J* = 1.4 Hz, 1H, aryl-H), 7.05 (d, *J* = 1.4 Hz, 1H, aryl-H), 6.82 (dd, *J* = 8.2, 1H, 2.2 Hz, aryl-H), 3.51 (s, 1H, SH), 3.38 (s, 1H, SH), 1.08 (s, 9H, *t*-Bu). ^13^C­{^1^H} NMR (126 MHz, C_6_D_6_): δ 150.2 (s, aryl-C), 131.7 (s, aryl-C), 131.4 (s, aryl-C), 128.2 (s, aryl-C), 128.0 (s, aryl-C), 124.3­(s, aryl-C), 34.3 (s, C­(CH_3_)_3_), 31.2 (s, C­(CH_3_)_3_).

#### Synthesis of Compound **2a** and **2b**

To a solution of 360 mg (1.81 mmol) of compound **2** in 10 mL pentane, 1.4 mL *n*-BuLi solution (3.63 mmol, 2.5 M in hexane) was added dropwise at room temperature. Upon the initial addition, a colorless precipitate was obtained. After stirring the mixture for an hour, the product was isolated via centrifugation and washed twice with pentane. After removing all volatiles *in vacuo*, a colorless product **2a** was obtained with an isolated yield of 78%. For crystallization, 20 mg (0.09 mmol) of compound **2a** was mixed with 22 mg (0.19 mmol) of TMEDA in a 2 mL solution of diethyl ether. The mixture was then filtered, and the filtrate was kept in the glovebox refrigerator at −33 °C. After 24 h, suitable colorless crystals for XRD measurement were obtained and characterized as **2b**.

**2a**: ^1^H NMR (500 MHz, THF-*d*_8_): δ 7.37 (d, *J* = 2.4 Hz, 1H, aryl-H), 7.17 (d, *J* = 8.0 Hz, 1H, aryl-H), 6.39 (dd, *J* = 8.0, 2.5 Hz, 1H, aryl-H), 1.17 (s, 9H, *t*-Bu). ^13^C­{^1^H} NMR (101 MHz, THF-*d*_8_): δ 150.7 (s, aryl-C), 147.8 (s, aryl-C), 142.9 (s, aryl-C), 133.1 (s, aryl-C), 130.6 (s, aryl-C), 117.6 (s, aryl-C), 34.3 (s, C­(CH_3_)_3_), 32.3 (s, C­(CH_3_)_3_)). ^7^Li NMR (194 MHz, THF-*d*_8_): δ 3.3 (s).

**2b**: ^1^H NMR (400 MHz, C_6_D_6_): δ 8.01 (s, 1H, aryl-H), 7.93 (d, *J* = 7.9 Hz, 1H, aryl-H), 6.87 (d, *J* = 7.9, 1H, aryl-H), 2.09 (s, 24H, 2­(C*H*_3_)_2_NCH_2_CH_2_N­(C*H*_3_)_2_), 1.91 (s, 6H, 2­(CH_3_)_2_NC*H*_2_C*H*_2_N­(CH_3_)_2_), 1.37 (s, 9H, *t*-Bu). ^13^C­{^1^H} NMR (101 MHz, C_6_D_6_): δ 148.4 (s, aryl-C), 145.6 (s, aryl-C), 143.7 (s, aryl-C), 133.5 (s, aryl-C), 131.1 (s, aryl-C), 118.6 (s, aryl-C), 57.0 (s, (CH_3_)_2_N*C*H_2_ *C*H_2_N­(CH_3_)_2_), 45.8 (s, (*C*H_3_)_2_NCH_2_CH_2_N­(*C*H_3_)_2_), 34.0 (s, C­(CH_3_)_3_), 32.0 (s, C­(CH_3_)_3_). ^7^Li NMR (194 MHz, C_6_D_6_): δ −1.2(s).

#### Synthesis of Compound **3**

To a solution of 5 g (30.07 mmol) of **1** along with TMEDA (60.01 mmol) in 50 mL hexane, 24 mL (60.01 mmol) *n*-BuLi solution (2.5 M in hexane) were added dropwise at room temperature. After stirring for 24 h, gray selenium powder (20% excess) (2.8 g, 36.08 mmol) was added portion wise at room temperature. The suspension was stirred for additional 12 h. The hexane was removed *in vacuo* and replaced with an equal volume of THF. The solution was cooled at 0 °C, and LiALH_4_ (1.7 g) was added. After the initial exothermic reaction subsided, the solution was refluxed for 8 h. The solution was cooled to room temperature and poured into 2 M HCl. The solution was extracted with ether (2 × 150 mL). The solvents were removed via a rotary evaporator. The residue was subsequently distilled to afford compound **3**, which appeared as a yellow oil with unpleasant odor (1.84 g, 25% yield): bp: 105 °C at 5.4 × 10^–2^ mb.

^**1**^ **H NMR (500 MHz, CDCl**_**3**_ **)**: δ 7.53 (d, *J* = 2.2 Hz, 1H, aryl-H), 7.31 (d, *J* = 8.2 Hz, 1H, aryl-H), 7.15 (dd, *J* = 8.2, 2.1 Hz, 1H, aryl-H), 3.75 (s, 1H, SH), 1.86 (s, ^1^ *J*_SeH_ = 53.6 Hz, 1H, SeH), 1.28 (s, 9H, *t*-Bu). ^13^C­{^1^H} NMR (126 MHz, CDCl_3_): δ 150.5 (s, aryl-C), 131.2 (s, aryl-C), 131.0 (s, aryl-C), 129.8 (s, aryl-C), 127.5 (s, aryl-C), 125.1 (s, aryl-C), 34.5 (s, C­(CH_3_)_3_), 31.5 (s, C­(CH_3_)_3_). ^77^Se NMR (95 MHz, C_6_D_6_): 141.8 (d, ^1^ *J*_SeH_ = 53.6 Hz,). MS (ESI-HR) [*m*/*z*]: 244.9904 ([M - H]^−^), calculated for [C_10_H_13_SSe]^−^ = 244.9898. Elemental analysis [%]: calculated: C 48.98, H 5.75, S 13.07 found: C 49.39, H 5.78, S 13.22.

#### Synthesis of Compound **3a** and **3b**

Compound **3a** was prepared in a similar way as compound **2a** with an isolated yield of 71%. Compound **3b** was crystallized with TMEDA, following the procedure established for compound **2b**.

**3a**: ^1^H NMR (500 MHz, THF-*d*_8_): δ 7.62 (d, *J* = 2.4 Hz, 1H, aryl-H), 7.19 (d, *J* = 8.0 Hz, 1H, aryl-H), 6.48 (dd, *J* = 7.4, 2.1 Hz, 1H, aryl-H), 1.17 (s, 9H, *t*-Bu). ^13^C­{^1^H} NMR (126 MHz, THF-*d*_8_): δ 150.0 (s, aryl-C), 142.9 (s, aryl-C), 142.8 (s, aryl-C), 133.8 (s, aryl-C), 132.7 (s, aryl-C), 119.0 (s, aryl-C), 34.2 (s, C­(CH_3_)_3_), 32.2 (s, C­(CH_3_)_3_)). ^7^Li NMR (194 MHz, THF-*d*_8_): δ 1.5 (s). ^77^Se­{^1^H} NMR (95 MHz, THF-*d*_8_): 8.1 (s).

#### Synthesis of Compound **4a**

A suspension of 150 mg of compound **2a** (0.71 mmol) in toluene was treated with 92 mg (0.04 mL, 0.35 mmol) of SnCl_4_ in toluene, added dropwise at −110 °C temperature. Upon the addition of the first drop, a red colored precipitate formed. After an hour of stirring, the mixture was heated to reflux and filtered while hot. All volatiles were removed under reduced pressure from the collected filtrate and red product **4a** was obtained in a yield of 60%. Alternatively, compound **2** was refluxed with SnCl_4_ in toluene under argon, yielding the red colored product as well.

**^1^H NMR (500 MHz, THF-*d*_8_)**: δ 7.54 (d, *J* = 2.2 Hz 1H, aryl-H), 7.39 (d, *J* = 8.3 Hz, 1H, aryl-H), 6.94 (dd, *J* = 8.3, 2.2 Hz, 1H, aryl-H), 1.27 (s, 9H, *t*-Bu). ^13^C­{^1^H} NMR (126 MHz, THF-*d*_8_): δ 147.9 (s, aryl-C), 138.5 (s, aryl-C), 135.4 (s, aryl-C), 129.0 (s, aryl-C), 126.1 (s, aryl-C), 122.4 (s, aryl-C), 35.0 (s, C­(CH_3_)_3_), 31.8 (s, C­(CH_3_)_3_). ^119^Sn {^1^H} NMR (186 MHz, THF-*d*_8_): −77.3 (s). MS (APCI-DIP-HR) [*m*/*z*]: 512.9843 ([M + H]^+^), calculated for [C_20_H_25_S_4_Sn]^+^ = 512.9856. Elemental analysis [%]: calculated: C 46.98, H 4.73, S 25.08; found: C 47.29, H 4.76, S 25.35.

#### Synthesis of Compound **4b**

A suspension of 100 mg of compound **2a** (0.48 mmol) in toluene was treated with 51 mg (0.03 mL, 0.24 mmol) of GeCl_4_ in toluene, added dropwise at room temperature. After filtration, all volatiles were removed under reduced pressure to afford a colorless solid. Recrystallization from hexane yielded compound **4b** in 86% yield. Alternatively, compound **2** was refluxed with GeCl_4_ in toluene under argon, yielding the colorless product **4b** once again. Single crystals of **4b** suitable for X-ray diffraction analysis were isolated from a hexane or toluene solution after refluxing and gradually cooling.

**^1^H NMR (500 MHz, C_6_D_6_)**: δ 7.40 (d, *J* = 1.9 Hz, 1H, aryl-H), 7.13 (dd, *J* = 8.4 Hz, 0.9 Hz, 1H, aryl-H), 6.78 (m, 1H, aryl-H), 1.06 (s, 9H, *t*-Bu). ^13^C­{^1^H} NMR (126 MHz, C_6_D_6_): δ 149.4 (s, aryl-C), 135.2 (s, aryl-C), 131.9 (s, aryl-C), 126.8 (s, aryl-C), 123.9 (s, aryl-C), 123.6 (s, aryl-C), 34.1 (s, C­(CH_3_)_3_), 31.1 (s, C­(CH_3_)_3_). MS (APCI-DIP-HR) [*m*/*z*]: 467.0032 ([M + H]^+^), calculated for [C_20_H_25_S_4_Ge]^+^ = 467.0045. Elemental analysis [%]: calculated: C 51.63, H 5.20, S 27.56; found: C 51.96, H 5.53, S 27.71.

#### Synthesis of Compound **5a**

A suspension of 150 mg of compound **3a** (0.58 mmol) in toluene was treated with 76 mg (0.03 mL, 0.29 mmol) of SnCl_4_ in toluene, added dropwise at low (−110 °C) temperature. Upon the addition of the first drop, the solution turned orange with the formation of a precipitate. After stirring overnight, the solution turned yellow. The mixture was centrifuged and the yellow supernatant was separated and dried under reduced pressure. The yellow product was washed with pentane and dried *in vacuo* to afford the product **5a** in 90% yield. Alternatively, compound **5a** was refluxed with SnCl_4_ in toluene under argon, yielding the yellow product as well.

**^1^H NMR (500 MHz, THF-*d*_8_)**: δ 7.65 (dd, *J* = 2.1 Hz, 1.1 Hz, 1H, aryl-H), 7.51 (dd, *J* = 8.3 Hz, 1.1 Hz, 1H, aryl-H), 7.06 (m, 1H, aryl-H), 1.28 (s, 9H, *t*-Bu). ^13^C­{^1^H}-NMR (126 MHz, THF-*d*_8_): δ 148.8 (s, aryl-C), 137.7 (s, aryl-C), 135.8 (s, aryl-C), 129.3 (s, aryl-C), 128.3 (s, aryl-C), 124.1 (s, aryl-C), 35.1 (s, C­(CH_3_)_3_), 31.7 (s, C­(CH_3_)_3_). ^119^Sn {^1^H} NMR (186 MHz, THF-*d*_8_): −31.8 (s, ^1^ *J*_SnSe_ = 1553.8 Hz). ^119^Sn {^1^H} NMR (186 MHz, Tol-*d*_8_): 92.8 (s). ^119^Sn {^1^H} NMR (186 MHz, C_6_D_12_): 98.0 (s,). ^77^Se {^1^H} NMR (95 MHz, THF-*d*_8_): 210.3 (s, ^1^ *J*_SnSe_ = 1553.4 Hz). MS (APCI-DIP-HR) [*m*/*z*]: 608.8734 ([M + H]^+^), calculated for [C_20_H_25_S_2_Se_2_Sn]^+^ = 608.8745. Elemental analysis [%]: calculated: C 39.70, H 4.00, S 10.60; found: C 39.86, H 4.00, S 10.83.

#### Synthesis of Compound **5b**

A suspension of 130 mg of compound **3a** (0.51 mmol) in toluene was treated with 54 mg (0.03 mL, 0.25 mmol) of GeCl_4_ in toluene, added dropwise at room temperature. After filtration, all volatiles were removed under reduced pressure to afford a colorless solid. Recrystallization using hexane yielded product **5b** in 79% yield. Alternatively, compound **3** was refluxed with GeCl_4_ in toluene under argon, yielding the colorless product **5b** once again. Single crystals of **5b** suitable for X-ray diffraction analysis were isolated from a hexane or toluene solution after refluxing and gradually cooling.

**^1^H NMR (500 MHz, C_6_D_6_)**: δ 7.41 (d, *J* = 2.3 Hz, 1H, aryl-H), 7.23 (d, *J* = 8.4 Hz, 1H, aryl-H), 6.78 (dd, *J* = 8.4 Hz, 2 Hz, 1H, aryl-H), 1.04 (s, 9H, *t*-Bu). ^13^C­{^1^H} NMR (126 MHz, C_6_D_6_): δ 149.2 (s, aryl-C), 135.5 (s, aryl-C), 134.9 (s, aryl-C), 126.8 (s, aryl-C), 126.0 (s, aryl-C), 124.2 (s, aryl-C), 34.4 (s, C­(CH_3_)_3_), 31.1 (s, C­(CH_3_)_3_). ^77^Se {^1^H} NMR (95 MHz, C_6_D_6_): 266.6 (s). MS (APCI-DIP-HR) [*m*/*z*]: 562.8918 ([M + H]^+^), calculated for [C_20_H_25_S_2_Se_2_Ge]^+^ = 562.8934. Elemental analysis [%]: calculated: C 42.97, H 4.33, S 11.47; found: C 43.26, H 4.52, S 11.46.

#### Synthesis of Compound **6a**

A 100 mg of compound **2** (0.50 mmol) in pentane was treated with 266 mg (0.50 mmol) of Pb­(N­(TMS)_2_)_2_ in pentane, added dropwise at room temperature. Upon the addition of the first drop, a yellow colored precipitate formed. After stirring the mixture for an hour, the product was isolated via centrifugation and washed twice with pentane. After removing all volatiles *in vacuo*, yellow product **6a** was obtained with an isolated yield of 91%. The compound **6a** crystallized from pyridine as its pyridine adduct **6b** in the form of yellow needles.

^**1**^ **H NMR (500 MHz, C**_**5**_ **D**_**5**_ **N):** δ 7.90 (d, *J* = 2.2 Hz, 1H, aryl-H), 7.81 (d, *J* = 8.2 Hz, 1H, aryl-H), 6.88 (dd, *J* = 8.3 Hz, 2.2 Hz, 1H, aryl-H), 1.23 (s, 9H, *t*-Bu). ^13^C­{^1^H}-NMR (126 MHz, C_5_D_5_N): δ 149.2 (s, aryl-C), 145.9 (s, aryl-C), 145.5 (s, aryl-C), 137.7 (s, aryl-C), 134.9 (s, aryl-C), 120.5 (s, aryl-C), 34.3 (s, C­(CH_3_)_3_), 32.1 (s, C­(CH_3_)_3_). ^207^Pb NMR (105 MHz, C_5_D_5_N): 2784.2 (s). MS (APCI-DIP-HR) [*m*/*z*]: 405.0213 [M + H]^+^, calculated for [C_10_H_13_S_2_Pb]^+^ = 405.0220 and [*m*/*z*]: 809.0356 [2M+H]^+^, calculated for [C_20_H_25_S_4_Pb]^+^ = 809.0366. Elemental analysis [%]: calculated: C 29.77, H 3.00, S 15.89; found: C 29.81, H 3.27, S 15.67.

**6b**: Elemental analysis [%]: calculated for [C_25_H_29_NPb_2_S_4_]: C 33.89, H 3.30, S 14.47; found: C 34.09, H 3.30, S 14.58.

#### Synthesis of Compound **7a**

A 100 mg of compound **3** (0.41 mmol) in pentane was treated with 215 mg (0.41 mmol) of Pb­(N­(TMS)_2_)_2_ in pentane, added dropwise at room temperature. Upon the addition of the first drop, an orange-colored precipitate formed. After stirring the mixture for an hour, the product was isolated via centrifugation and washed twice with pentane. After removing all volatiles *in vacuo*, an orange product **7a** was obtained with an isolated yield of 92%.

^**1**^ **H NMR (500 MHz, C**_**5**_ **D**_**5**_ **N):** δ 8.08­(d, *J* = 2.2 Hz, 1H, aryl-H), 7.84 (d, *J* = 8.2 Hz, 1H, aryl-H), 6.91 (dd, *J* = 8.3 Hz, 2.2 Hz, 1H, aryl-H), 1.22 (s, 9H, *t*-Bu). ^13^C­{^1^H}-NMR (126 MHz, C_5_D_5_N): δ 149.7 (s, aryl-C), 145.4 (s, aryl-C), 142.8 (s, aryl-C), 138.7 (s, aryl-C), 137.7­(s, aryl-C), 120.8 (s, aryl-C), 34.1 (s, C­(CH_3_)_3_), 31.9 (s, C­(CH_3_)_3_)). ^77^Se NMR (95 MHz, C_5_D_5_N): 404.1 (s). ^207^Pb NMR (105 MHz, C_5_D_5_N): 2971.7 (s). MS (APCI-DIP-HR) [*m*/*z*]: 452.9660 [M + H]^+^, calculated for [C_10_H_13_SSePb]^+^ = 452.9664 and [*m*/*z*]: 904.9249 [2M+H]^+^, calculated for [C_20_H_25_S_2_Se_2_Pb]^+^ = 904.9255. Elemental analysis [%]: calculated: C 26.67, H 2.69, S 7.12 found: C 26.34, H 2.55, S 7.33.

#### Synthesis of Compound **7b**

A suspension of compound **7a** (60 mg, 0.13 mmol) in toluene was treated dropwise at room temperature with a toluene solution of Im4Me (16 mg, 0.13 mmol). Upon the addition of the first drop, the orange solid **7a** began to dissolve, and the color of the solution turned to yellow. The reaction mixture was stirred for an hour at room temperature. The solvent was then removed *in vacuo* and resulting yellow solid which was washed with pentane and dried again. The final removal of residual solvent *in vacuo* afforded compound **7b** as a yellow solid in 92% isolated yield. Single crystals of **7b** for X-ray diffraction were obtained in a glovebox by slow diffusion of pentane into a dichloromethane solution.

^**1**^ **H NMR (500 MHz, C**_**5**_ **D**_**5**_ **N):** δ 8.01 (d, *J* = 1.5 Hz, 1H, aryl-H), 7.80 (d, *J* = 8.2 Hz, 1H, aryl-H), 6.86 (dt, *J* = 8.2 Hz, 1.6 Hz, 1H, aryl-H), 3.58 (s, 6H, NMe), 1.68 (s, 6H, C = CMe), 1.19 (s, 9H, *t*-Bu). ^13^C­{^1^H}-NMR (126 MHz, C_6_D_6_): δ 189.6 (s, NCN), 149.8 (s, aryl-C), 144.5 (s, aryl-C), 143.1 (s, aryl-C), 137.0 (s, aryl-C), 135.9 (s, aryl-C), 124.9 (s, aryl-C), 120.1 (s, C = C, Im4Me), 34.4 (s, NCH_3_), 33.9 (s, C­(CH_3_)_3_), 31.9 (s, C­(CH_3_)_3_)), 7.9 (s, C = CCH_3_). ^77^Se NMR (95 MHz, C_5_D_5_N): 252.5 (s). ^207^Pb NMR (105 MHz, Tol-*d*_8_): 2070.5 (s). MS (APCI-DIP-HR) [*m*/*z*]: 576.0582 [M]^+^, calculated for [C_17_H_24_N_2_SSePb]^+^ = 576.0586.

## Supplementary Material



## Notes

NMR, MS, UV–vis, HPLC, and crystallographic data (PDF)
