# Supplementary material for: Axially Chiral Spiro Compounds with Heavier Group 14 Elements as Spiro-Centers
Source: Inorg Chem. 2026 May 2;65(19):10592–602. doi: 10.1021/acs.inorgchem.6c00576 (PMC13188056; doi:10.1021/acs.inorgchem.6c00576)
Supplement: Supplementary file 1 [file ic6c00576_si_001.pdf]

Supporting Information

Axially Chiral Spiro Compounds with Heavier Group 14 Elements as Spiro-Centers

Aynura Mammadova, Clemens Bruhn and *Rudolf Pietschnig*[*]

Institute for Chemistry and CINSaT, University of Kassel, Heinrich-Plett-Straße 40, 34132 Kassel, Germany

E-mail: pietschnig@uni-kassel.de

Supporting Information (SI) File

Contents

1. NMR and Mass spectra *S3*

Figure S1. ^{1}H NMR spectrum (in C_6D_6) of compound 2 .... *S3*

Figure S2. ^{13}C{^{1}H} NMR spectrum (in C_6D_6) of compound 2. .... *S4*

Figure S3. ^{1}H NMR spectrum (in THF-d_8) of compound 2a. *S5*

Figure S4. ^{13}C{^{1}H} NMR spectrum (in THF-d_8) of compound 2a. *S6*

Figure S5. ^{7}Li NMR spectrum (in THF-d_8) of compound 2a. *S7*

Figure S6. ^{1}H NMR spectrum (in C_6D_6) of compound 2b with TMEDA. *S8*

Figure S7. ^{13}C{^{1}H} NMR spectrum (in C_6D_6) of compound 2b with TMEDA. *S9*

Figure S8. ^{7}Li NMR spectrum (in C_6D_6) of compound 2b with TMEDA. *S10*

Figure S9. ^{1}H NMR spectrum (in $CDCl_3$) of compound 3. .... *S11*

Figure S10. ^{13}C {^{1}H} NMR spectrum (in $CDCl_3$) of compound 3. .... *S12*

Figure S11. ^{77}Se NMR spectrum (in $CDCl_3$) of compound 3. .... *S13*

Figure S12. ^{77}Se{^{1}H} NMR spectrum (in $CDCl_3$) of compound 3. .... *S14*

Figure S13. ESI-HRMS of compound 3. .... *S15*

Figure S14. ^{1}H NMR spectrum (in THF-d_8) of compound 3a. *S16*

Figure S15. ^{13}C{^{1}H} NMR spectrum (in THF-d_8) of compound 3a. *S17*

Figure S16. ^{7}Li NMR spectrum (in THF-d_8) of compound 3a. *S18*

Figure S17. ^{77}Se{^{1}H} NMR spectrum (in THF-d_8) of compound 3a. *S19*

Figure S18. ^{1}H NMR spectrum (in THF-d_8) of compound 4a. *S20*

Figure S19. ^{13}C{^{1}H} NMR spectrum (in THF-d_8) of compound 4a. *S21*

Figure S20. ^{119}Sn{^{1}H} NMR spectrum (in THF-d_8) of compound 4a. *S22*

Figure S21. APCI-DIP-HRMS of compound 4a. *S23*

Figure S22. ^{1}H NMR spectrum (in THF-d_8) of compound 5a. *S24*

Figure S23. [31]C{^{1}H} NMR spectrum (in THF-d_8) of compound 5a. *S25*

Figure S24. ^{119}Sn{^{1}H} NMR spectrum (in THF-d_8) of compound 5a. *S26*

Figure S25. ^{77}Se NMR spectrum (in THF-d_8) of compound 5a. *S27*

Figure S26. ^{119}Sn{^{1}H} NMR spectrum (in Tol-d_8) of compound 5a. *S28*

Figure S27. ^{119}Sn{^{1}H} NMR spectrum (in C_6D_{12}) of compound 5a. *S29*

Figure S28. APCI-DIP-HRMS of compound 5a. *S30*

Figure S29. ^{1}H NMR spectrum (in C_6D_6) of compound 4b. *S31*

Figure S30. ^{13}C{H} NMR spectrum (in C_6D_6) of compound 4b. *S32*

Figure S31. APCI-DIP-HRMS of compound 4b. *S33*

Figure S32. ^{1}H NMR spectrum (in C_6D_6) of compound 5b. *S34*

Figure S33. ^{13}C{^{1}H} NMR spectrum (in C_6D_6) of compound 5b. *S35*

Figure S34. ^{77}Se{^{1}H} NMR spectrum (in C_6D_6) of compound 5b. *S36*

Figure S35. APCI-DIP-HRMS of compound 5b. *S37*

Figure S36. ^{1}H NMR spectrum (in C_5D_5N) of compound 6a. *S38*
Figure S37. $^{13}C\{^{1}H\}$ NMR spectrum (in C_5D_5N) of compound 6a. *S39*
Figure S38. ^{207}Pb NMR spectrum (in C_5D_5N) of compound 6a. *S40*
Table S1. ^{1}H DOSY-ECC-MW estimation of 6a in DMSO-d_6 at 25 °C. *S41*
Figure S39: ^{1}H DOSY spectrum of 6a in DMSO-d_6. .... *S42*
Figure S40. APCI-DIP-HRMS of compound 6a $[M+H]^+$. *S43*
Figure S41. APCI-DIP-HRMS of compound 6a $[2M+H]^+$. *S44*
Figure S42. ^{1}H NMR spectrum (in C_5D_5N) of compound 7a. *S45*
Figure S43. $^{13}C\{^{1}H\}$ NMR spectrum (in C_5D_5N) of compound 7a. *S46*
Figure S44. ^{77}Se NMR spectrum (in C_5D_5N) of compound 7a. *S47*
Figure S45. ^{207}Pb NMR spectrum (in C_5D_5N) of compound 7a. *S48*
Figure S46. APCI-DIP-HRMS of compound 7a $[M+H]^+$. *S49*
Figure S47. APCI-DIP-HRMS of compound 7a $[2M+H]^+$. *S50*
Figure S48. APCI-DIP-HRMS of compound 8 $[M+H]^+$. *S51*
Figure S49. ^{1}H NMR spectrum (in C_5D_5N) of compound 7b. *S52*
Figure S50. $^{13}C\{^{1}H\}$ NMR spectrum (in C_6D_6) of compound 7b. *S53*
Figure S51. ^{77}Se NMR spectrum (in C_5D_5N) of compound 7b. *S54*
Figure S52. ^{207}Pb NMR spectrum (in Tol-d_8) of compound 7b. *S55*
Figure S53. APCI-DIP-HRMS of compound 7b $[M]^+$. *S56*
2. *The molecular structure of compound 7e* *S57*
Figure S54. The molecular structure of compound 7e. *S57*
3. *UV-vis Data* *S58*
Figure S55. Normalized UV-Vis absorption spectra of 4b and 5b. *S58*
4. *HPLC Data* *S59*
Figure S56. The analytical HPLC results of compound 5b. *S59*
5. *IR Spectra* *S60*
Figure S57. IR spectra of compound 5a. *S60*
6. *Crystal data and structure refinement details.* *S61*
7. *Table S2. Crystal data and structure refinement for compounds 2b and 3b.* *S61*
Table S3. Crystal data and structure refinement for compounds 4b and 5b. *S62*
Table S4. Crystal data and structure refinement for compounds 6b and 7b. *S63*
Table S5. Crystal data and structure refinement for compounds 7c and 7d. *S64*
Table S6. Crystal data and structure refinement for compounds 7e and 9a. *S65*
8. *References* *S66*

1. NMR and Mass spectra

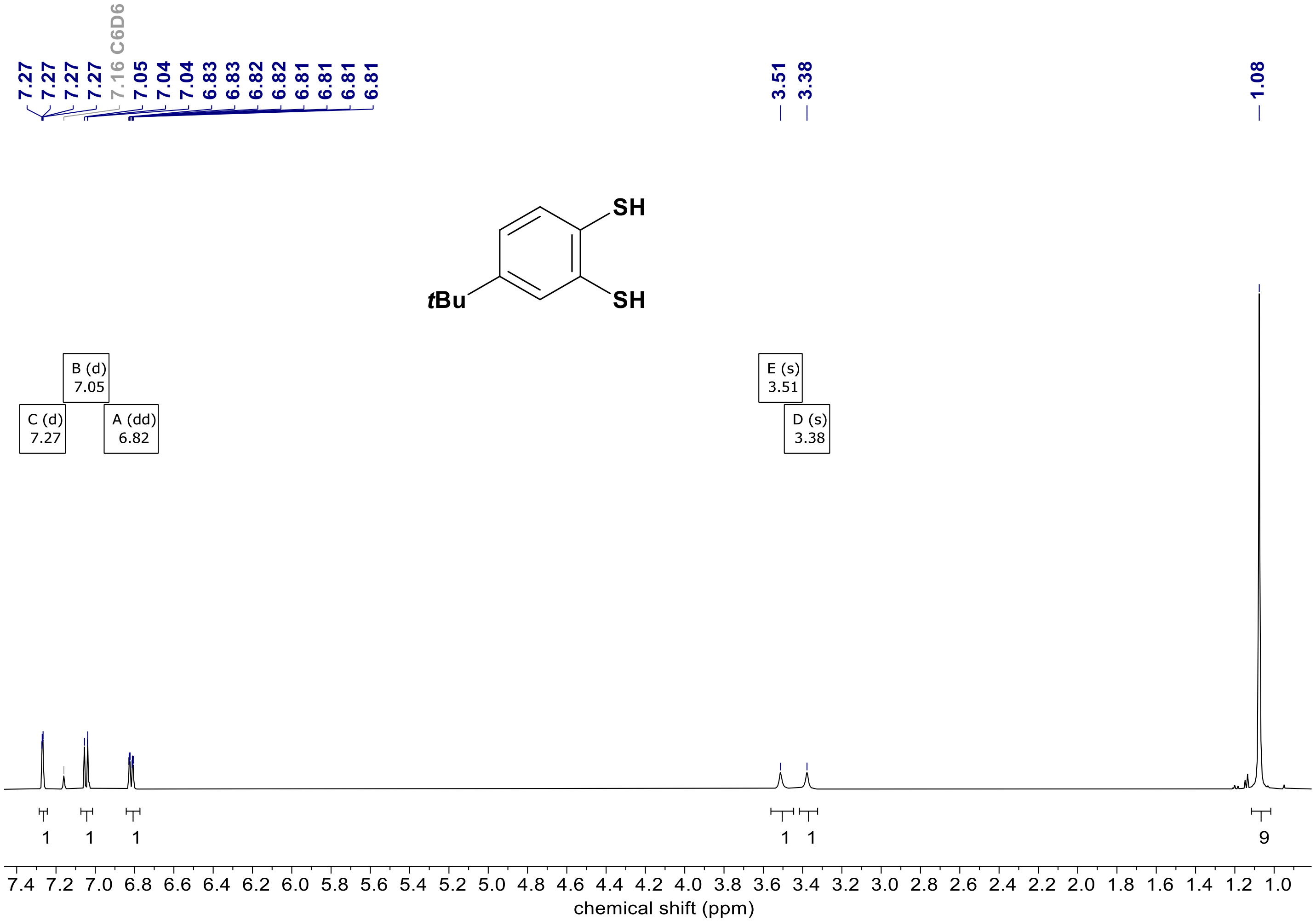

Figure S1. ^{1}**H NMR spectrum (in** C_6D_6**) of compound 2.**

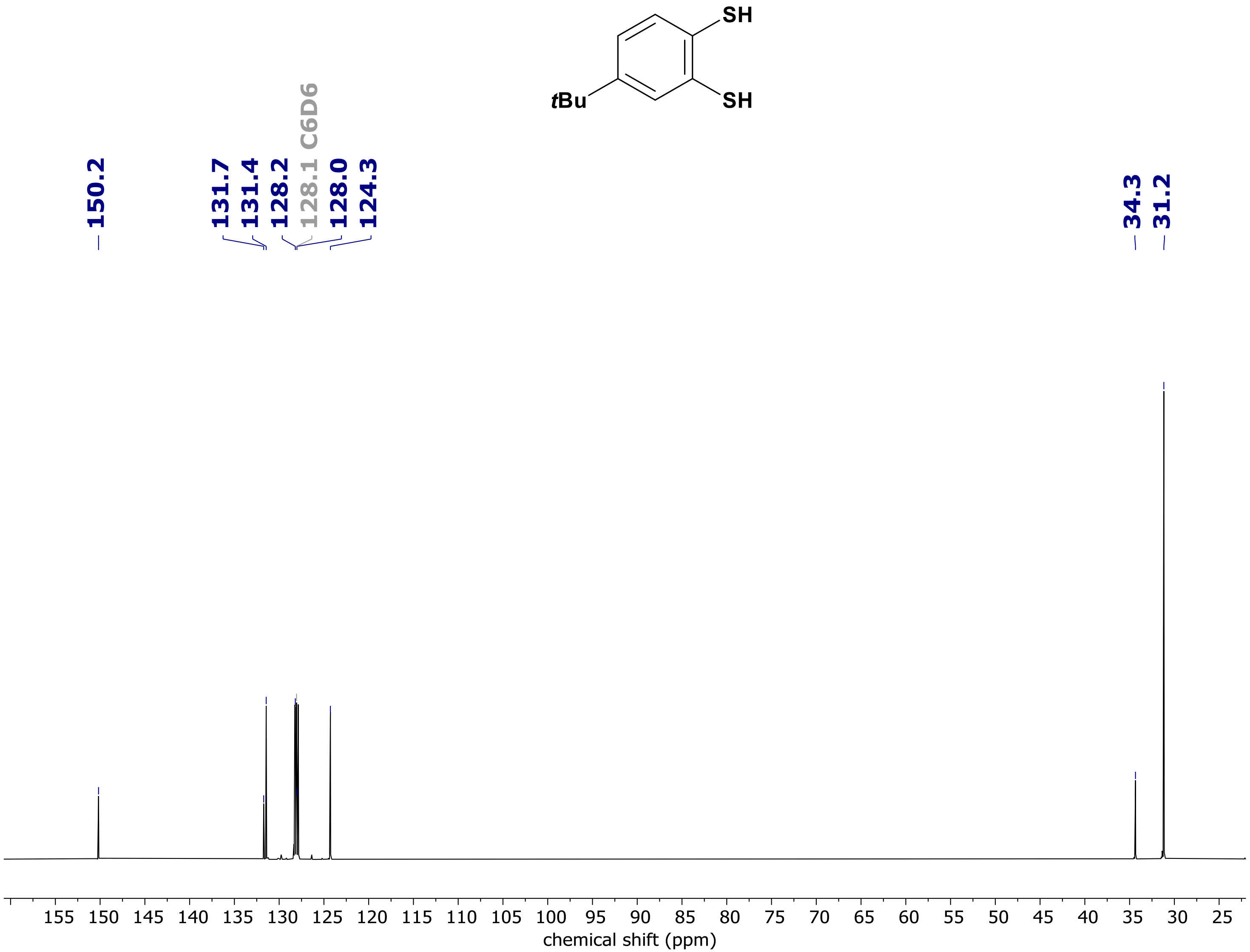

Figure S2. **$^{13}C\{^{1}H\}$ NMR spectrum (in C_6D_6) of compound 2.**

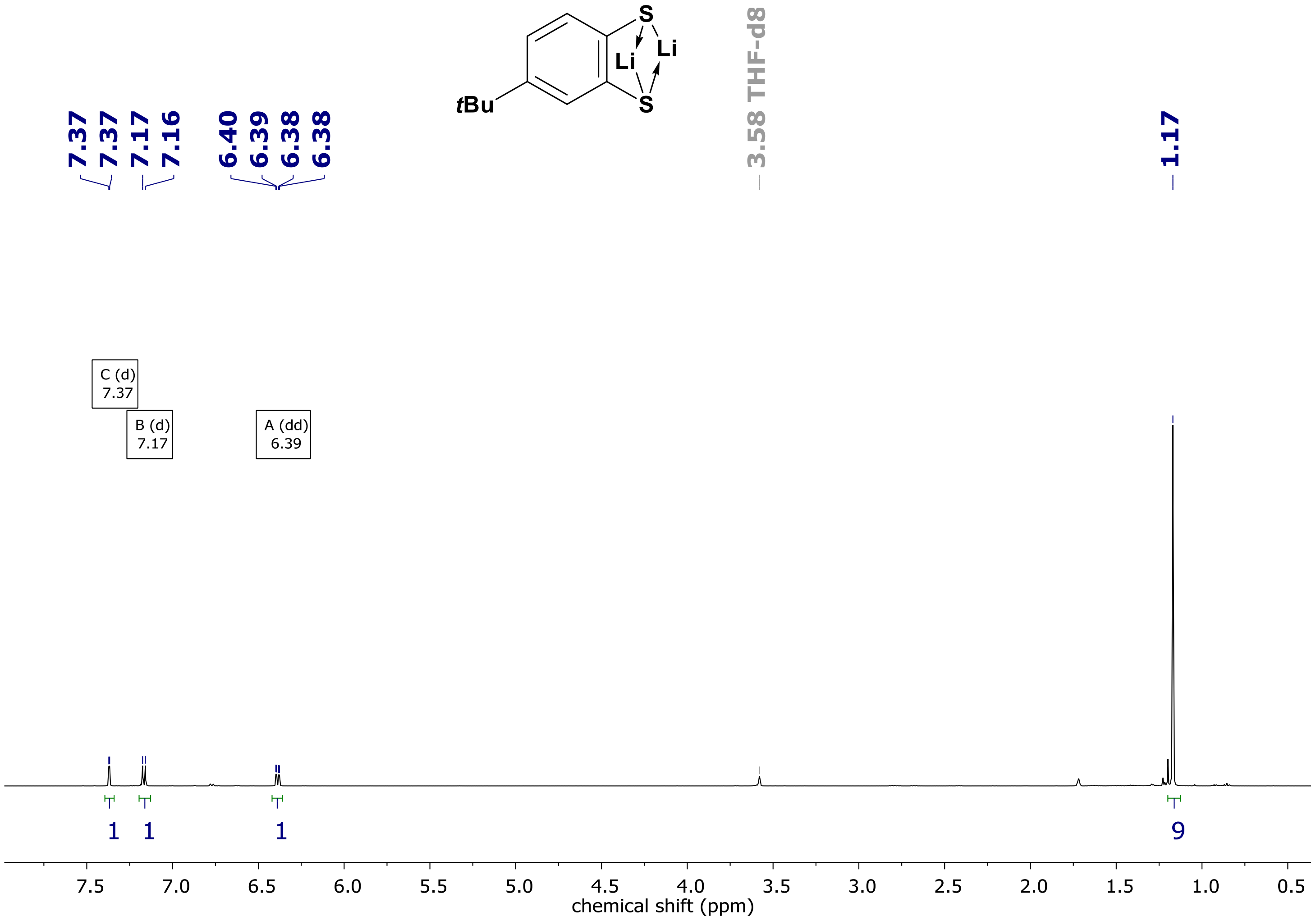

Figure S3. ^{1}H NMR spectrum (in THF-d_8) of compound 2a.

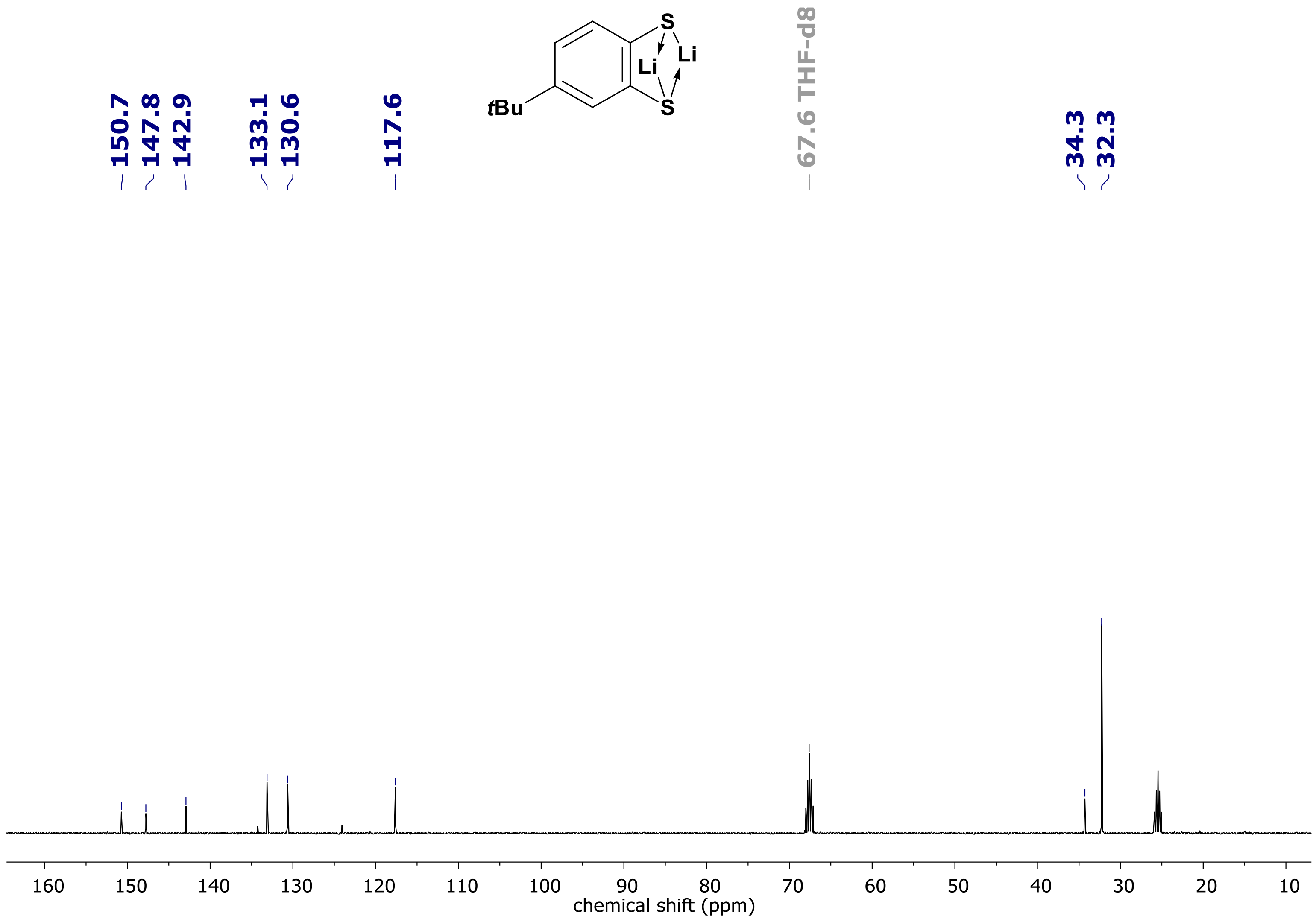

Figure S4. $^{13}C\{^{1}H\}$ NMR spectrum (in THF-d_8) of compound 2a.

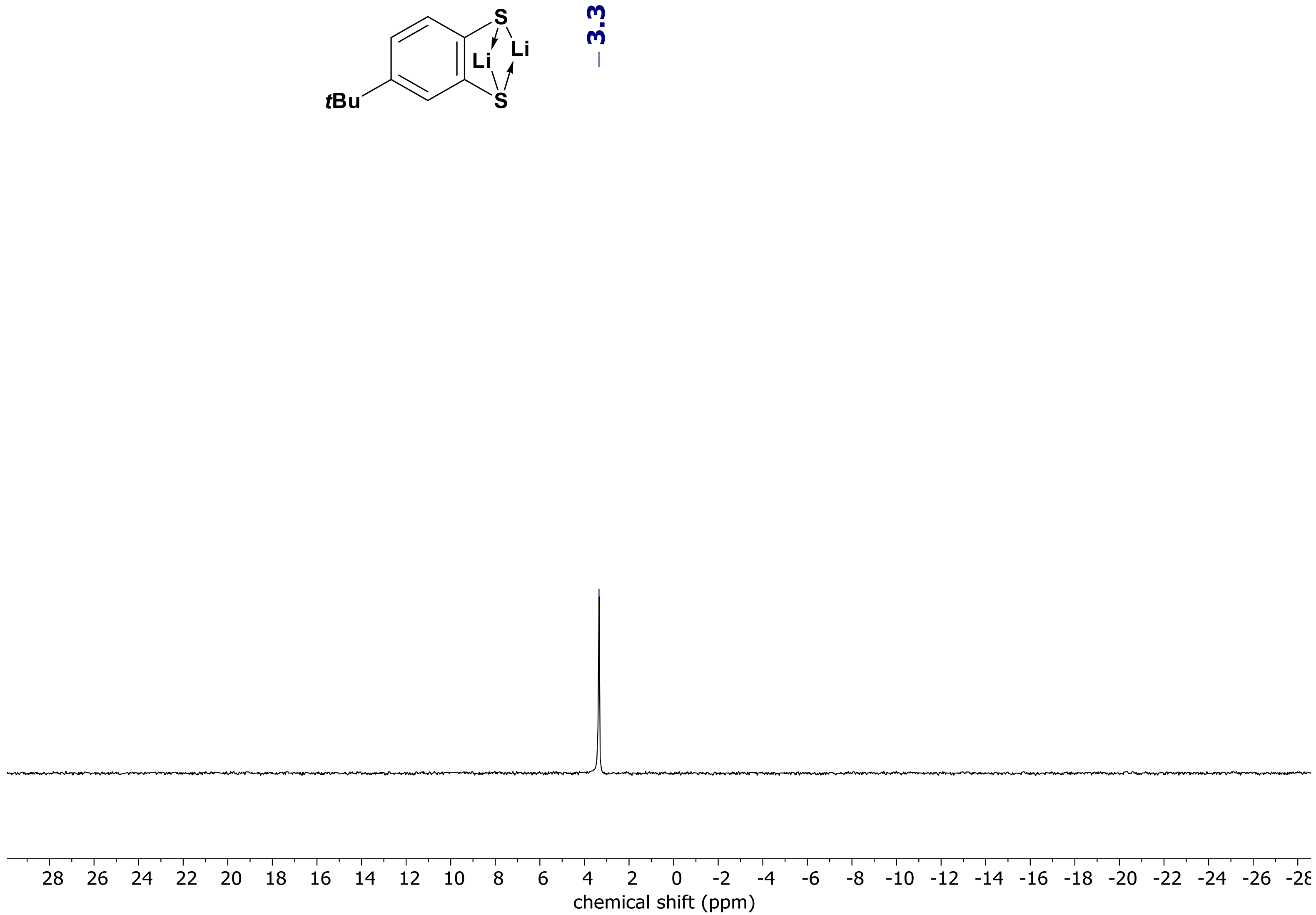

Figure S5. ^{7}Li NMR spectrum (in THF-d_8) of compound 2a.

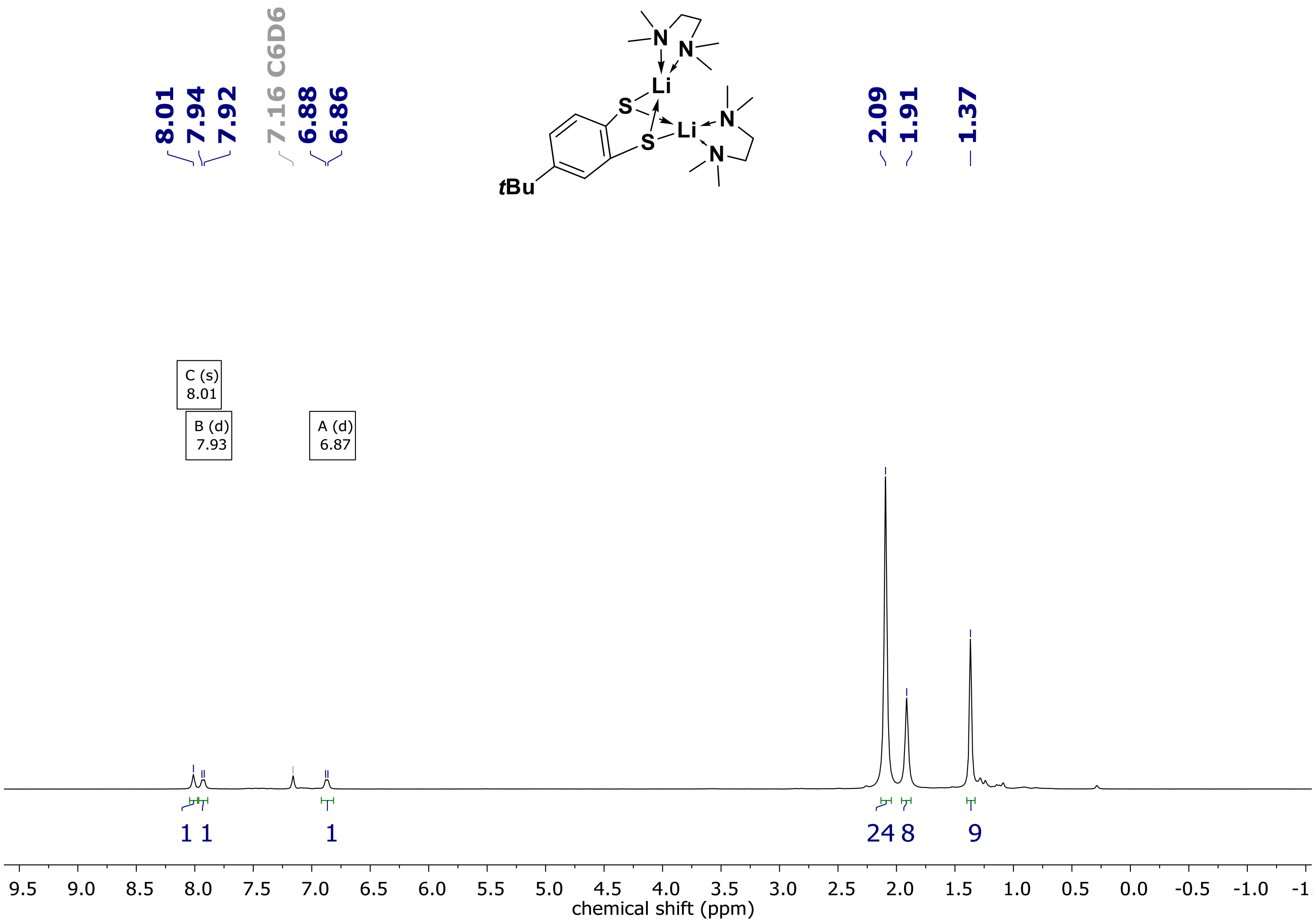

Figure S6. ^{1}H NMR spectrum (in C_6D_6) of compound 2b with TMEDA.

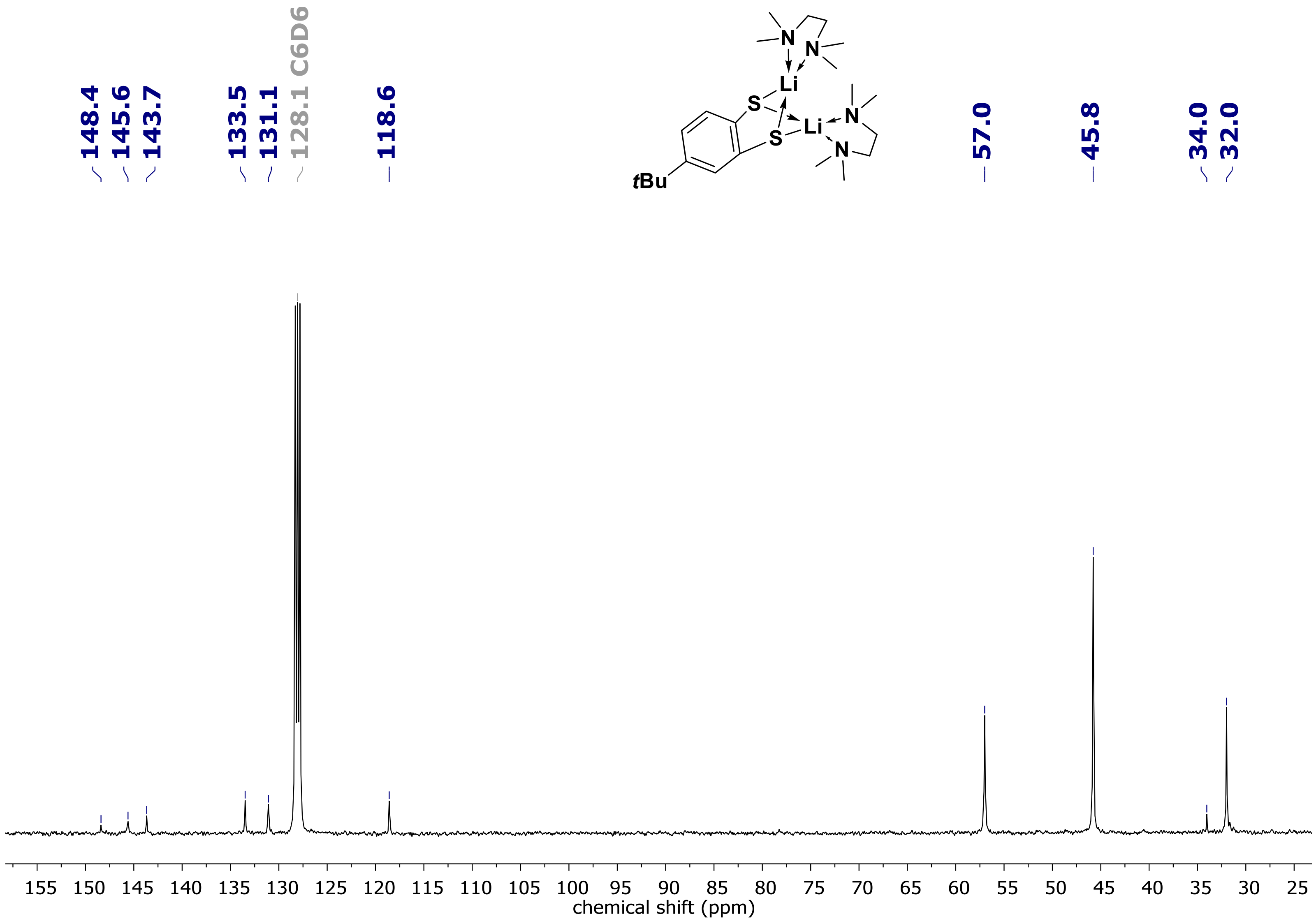

Figure S7. $^{13}C\{^1H\}$ **NMR spectrum (in** C_6D_6**) of compound 2b with TMEDA.**

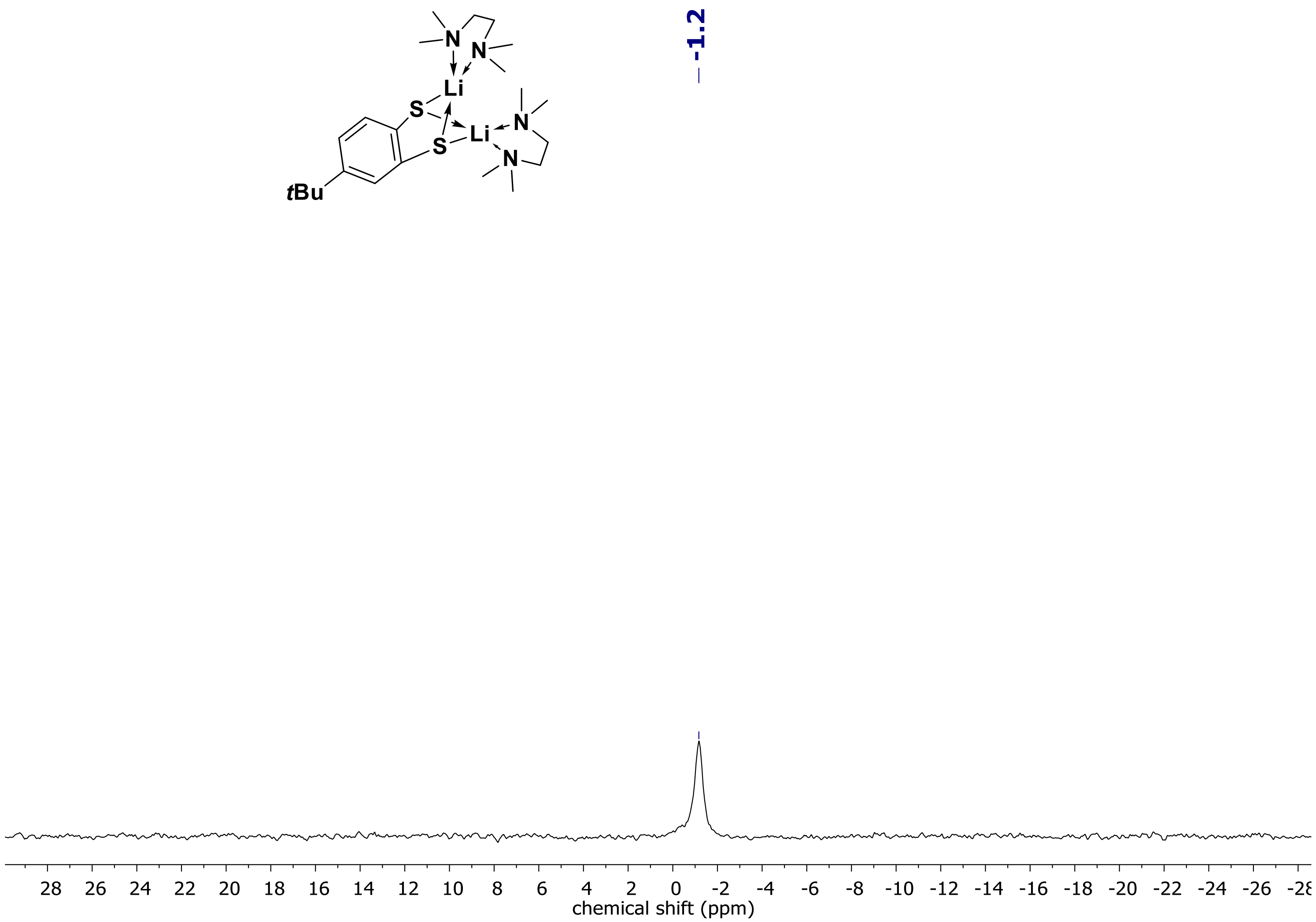

Figure S8. ^{7}Li NMR spectrum (in C_6D_6) of compound 2b with TMEDA.

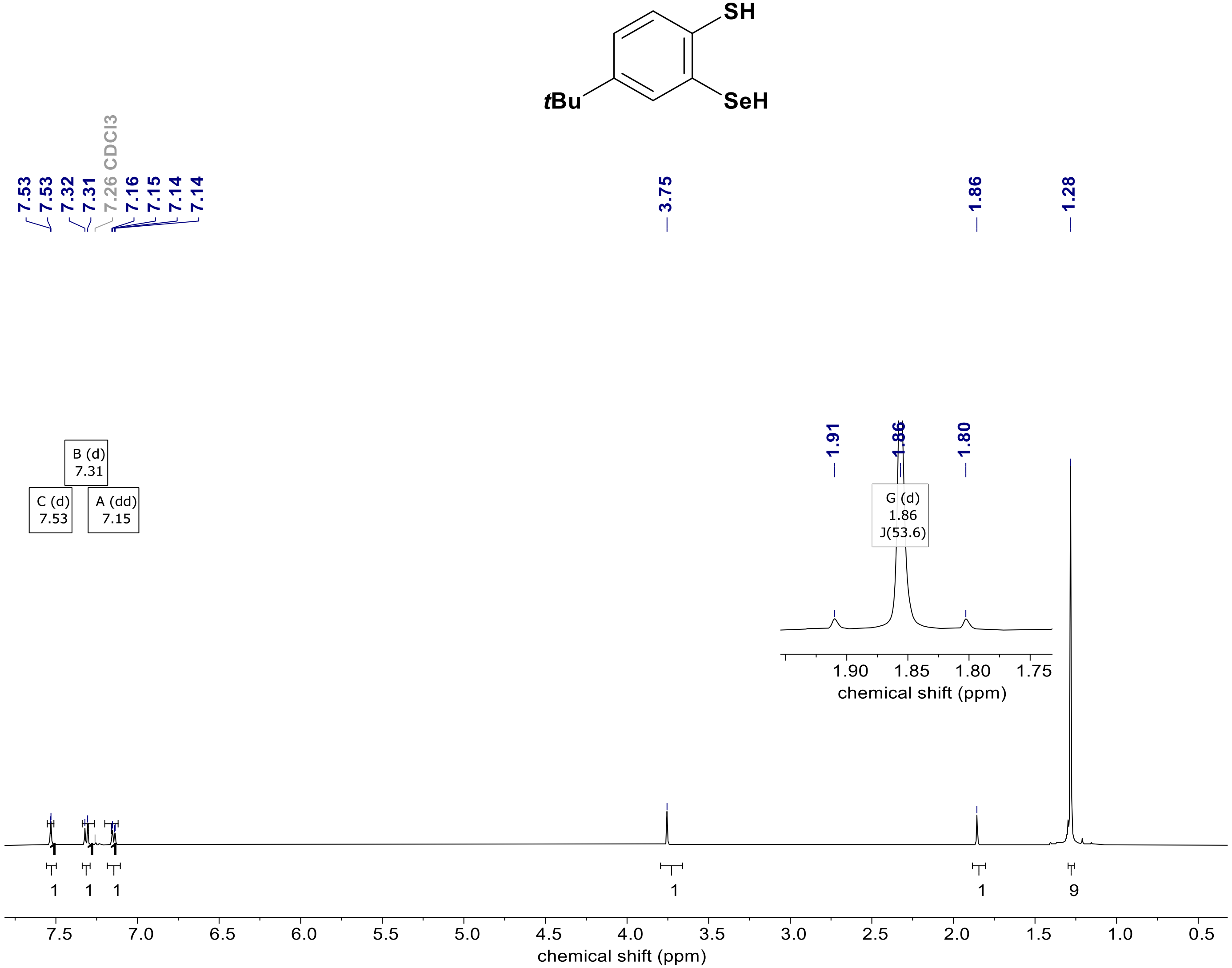

Figure S9. ^{1}H NMR spectrum (in $CDCl_3$) of compound 3.

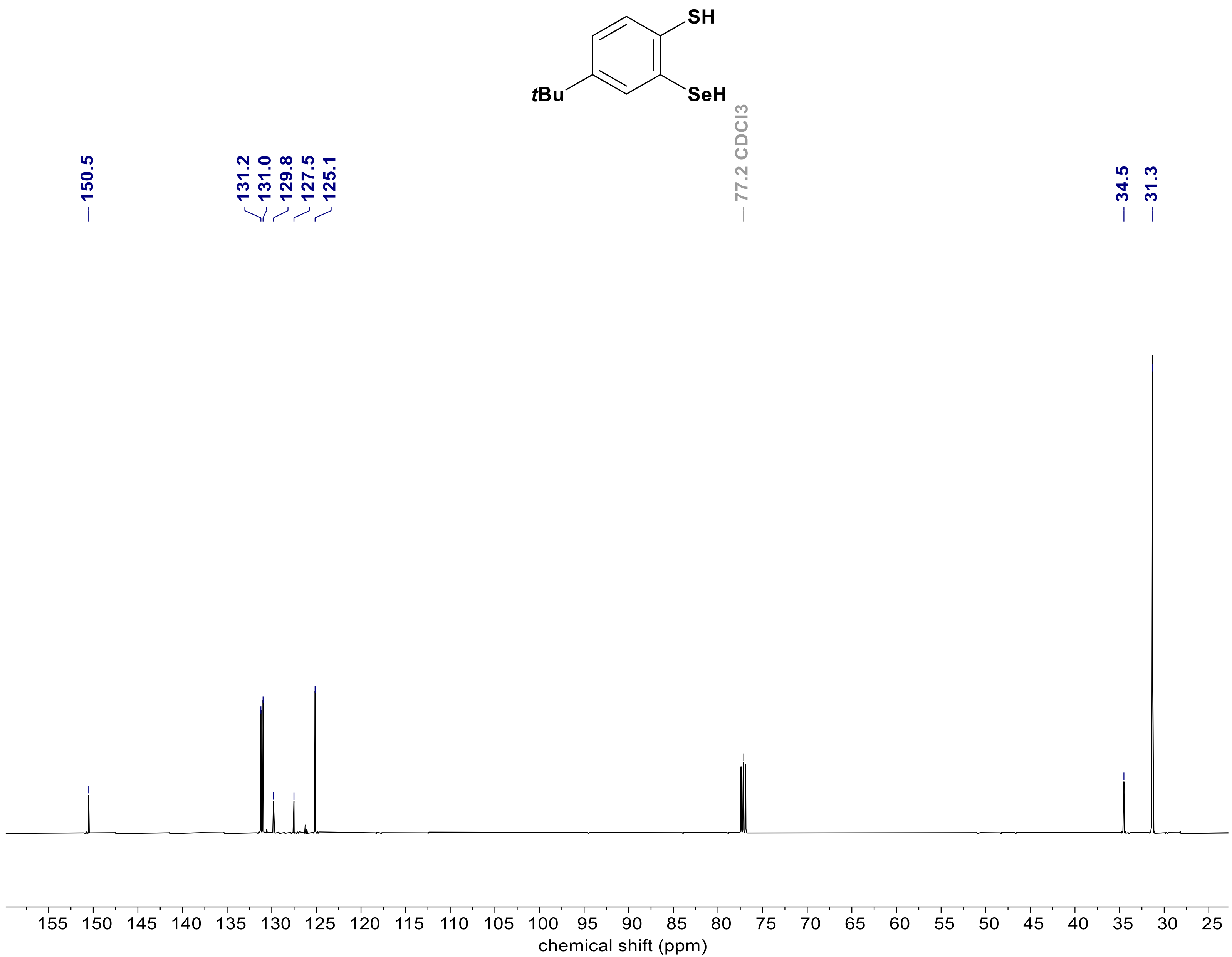

Figure S10. **^{13}C {^{1}H} NMR spectrum (in $CDCl_3$) of compound 3.**

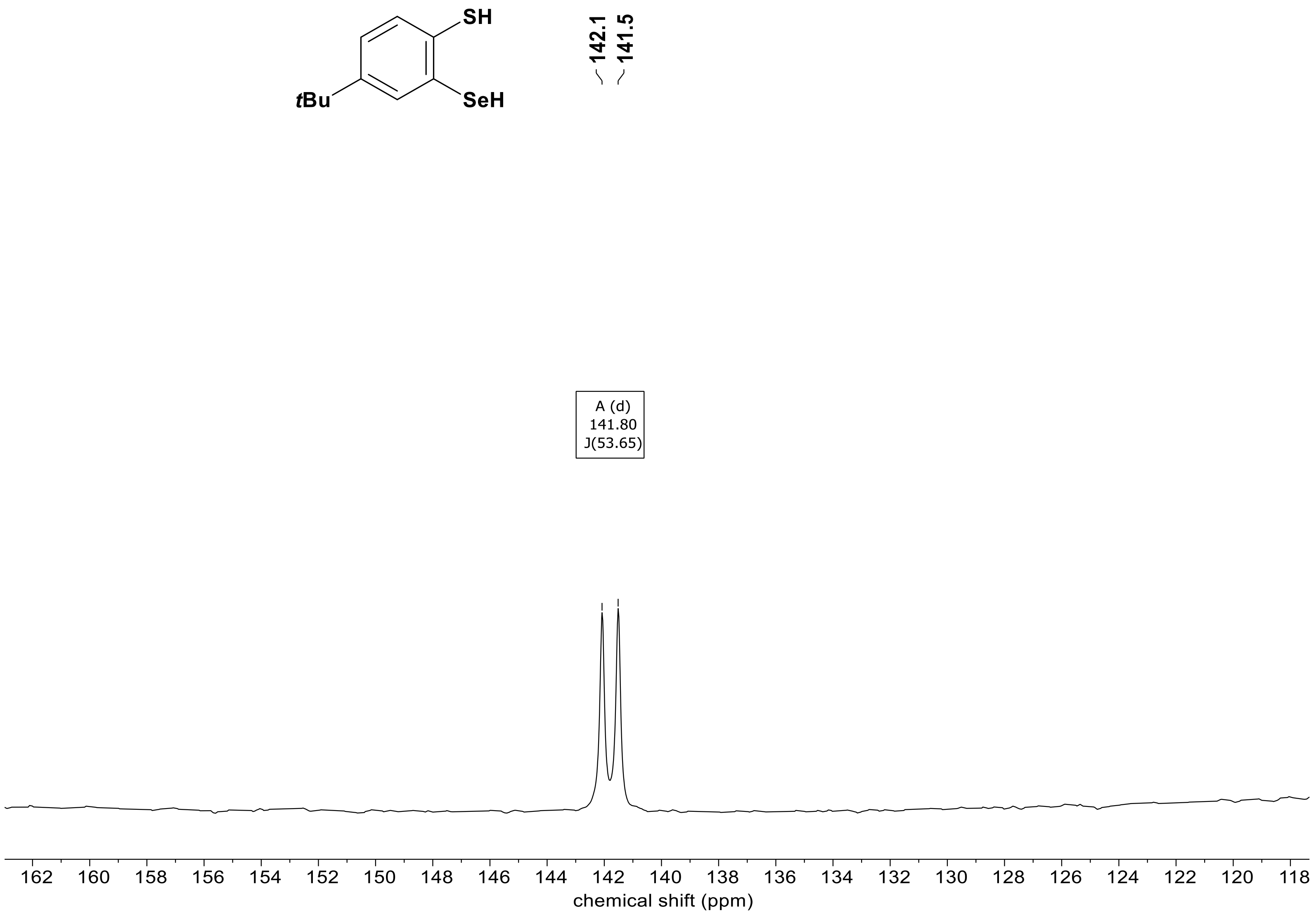

Figure S11. ^{77}**Se NMR spectrum (in CDCl**$_3$**) of compound 3.**

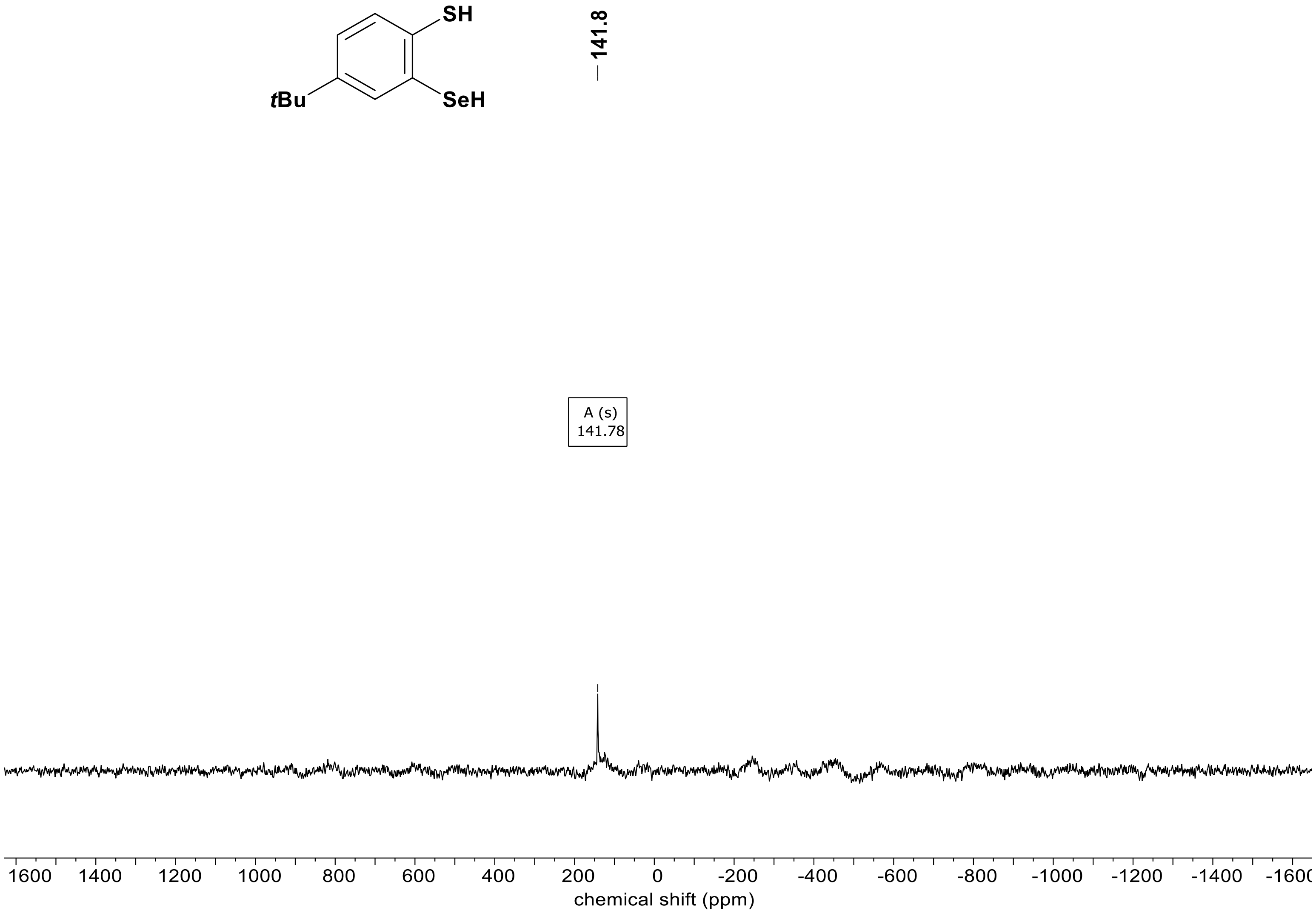

Figure S12. ^{77}Se{^{1}H} NMR spectrum (in $CDCl_3$) of compound 3.

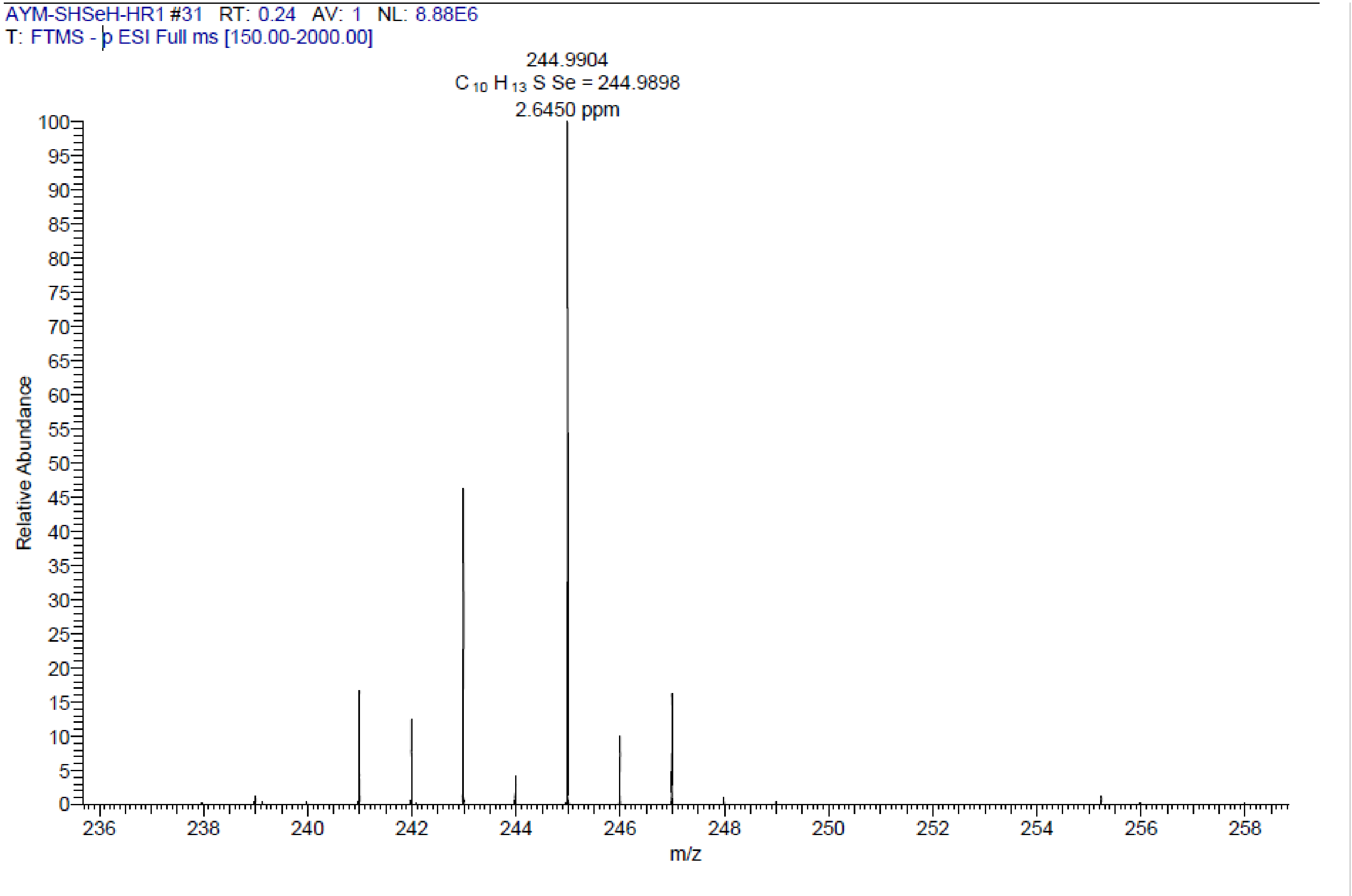

Figure S13. ESI-HRMS of compound 3.

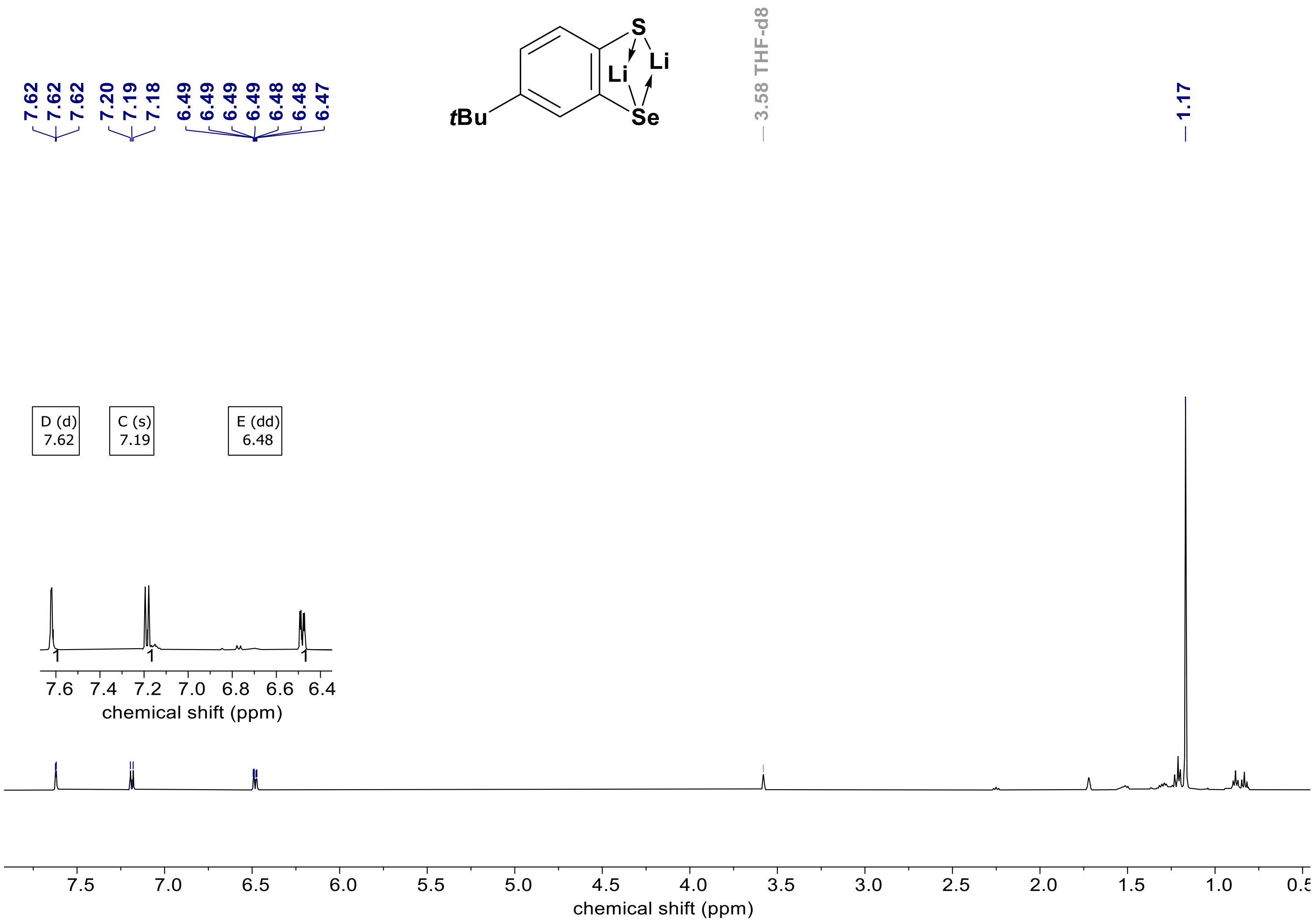

Figure S14. ^{1}H NMR spectrum (in THF-d_8) of compound 3a.

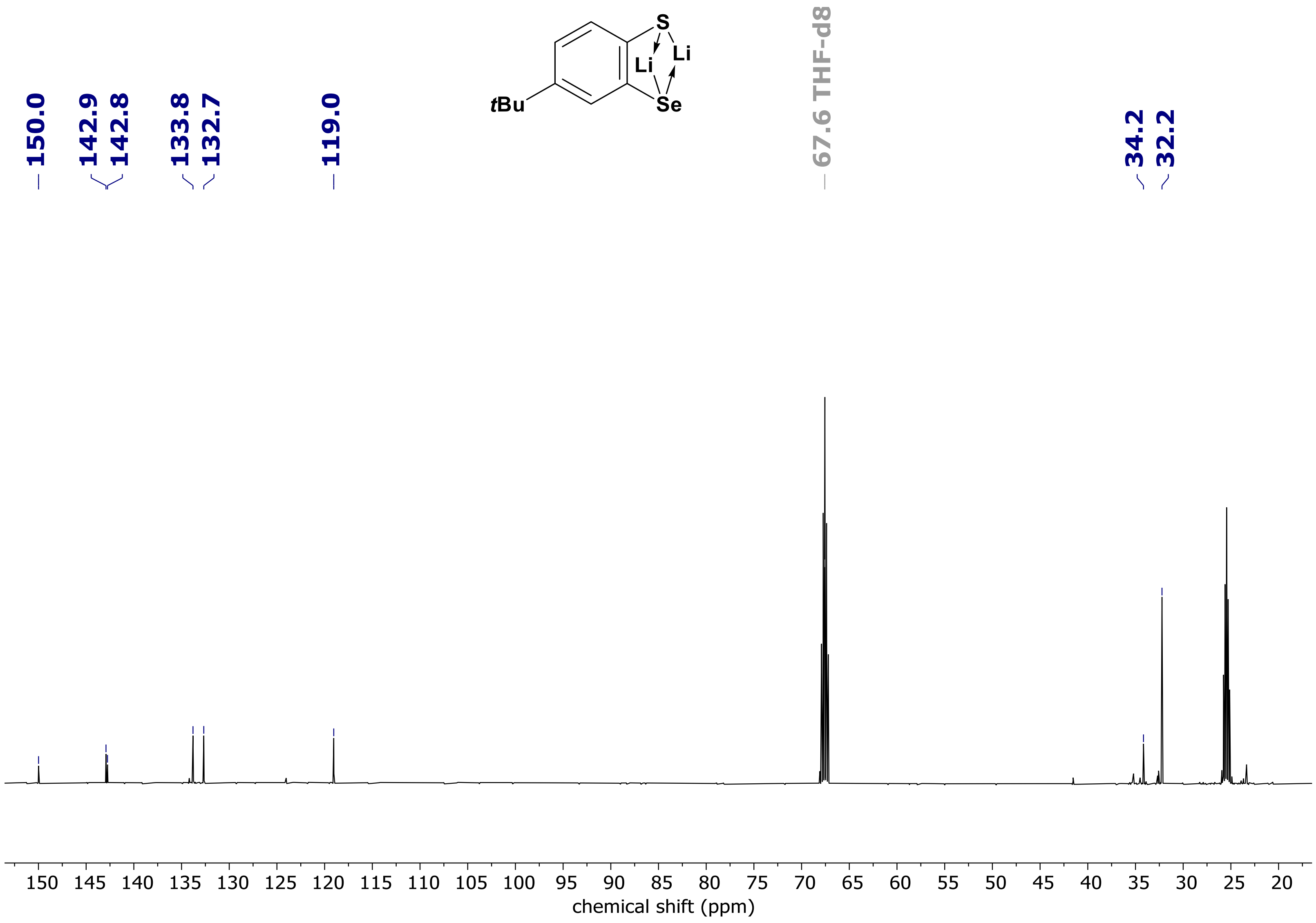

Figure S15. $^{13}C\{^{1}H\}$ **NMR spectrum (in THF-d_8) of compound 3a.**

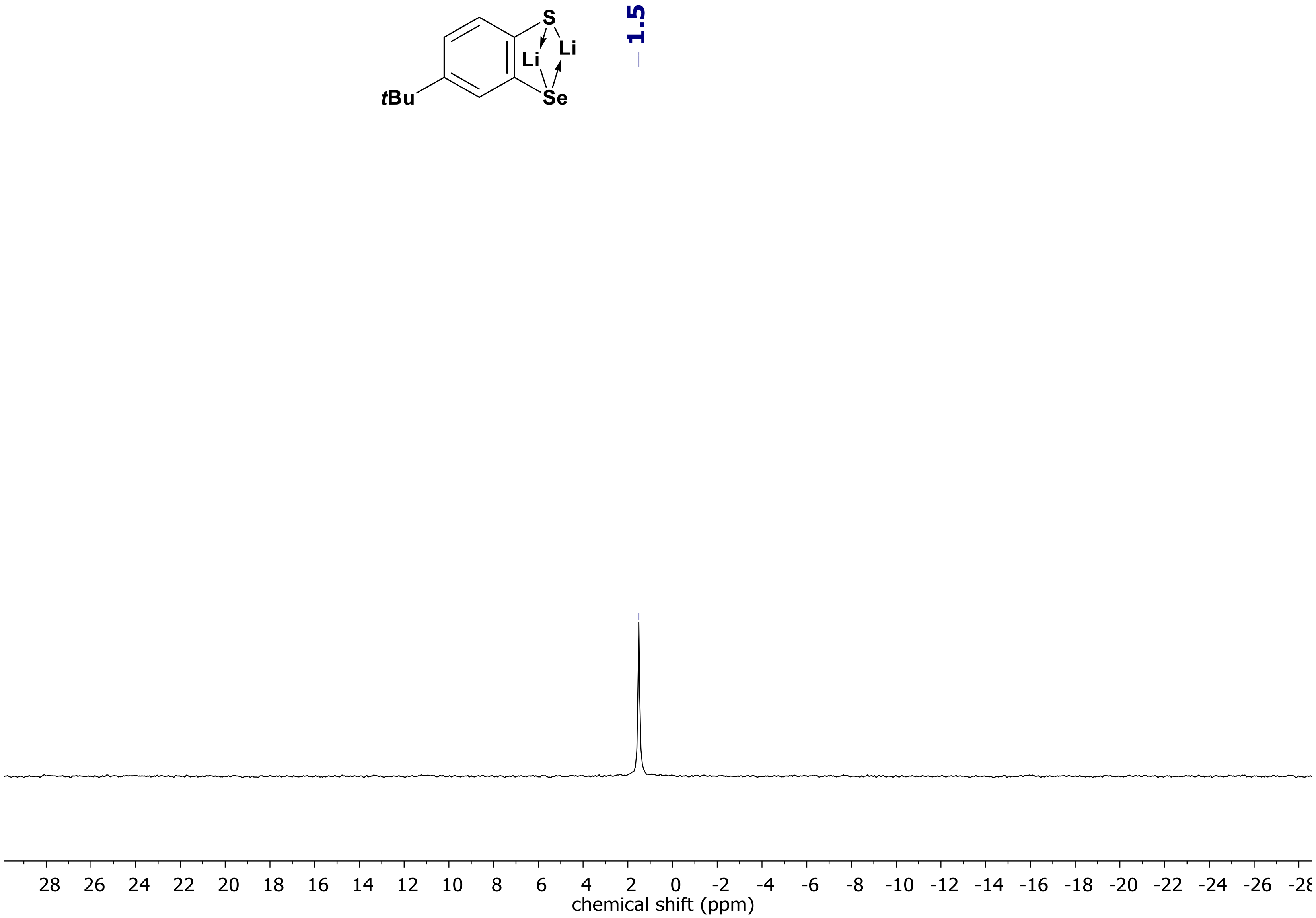

Figure S16. ^{7}Li NMR spectrum (in THF-d_8) of compound 3a.

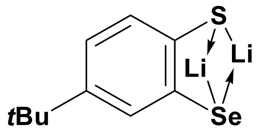

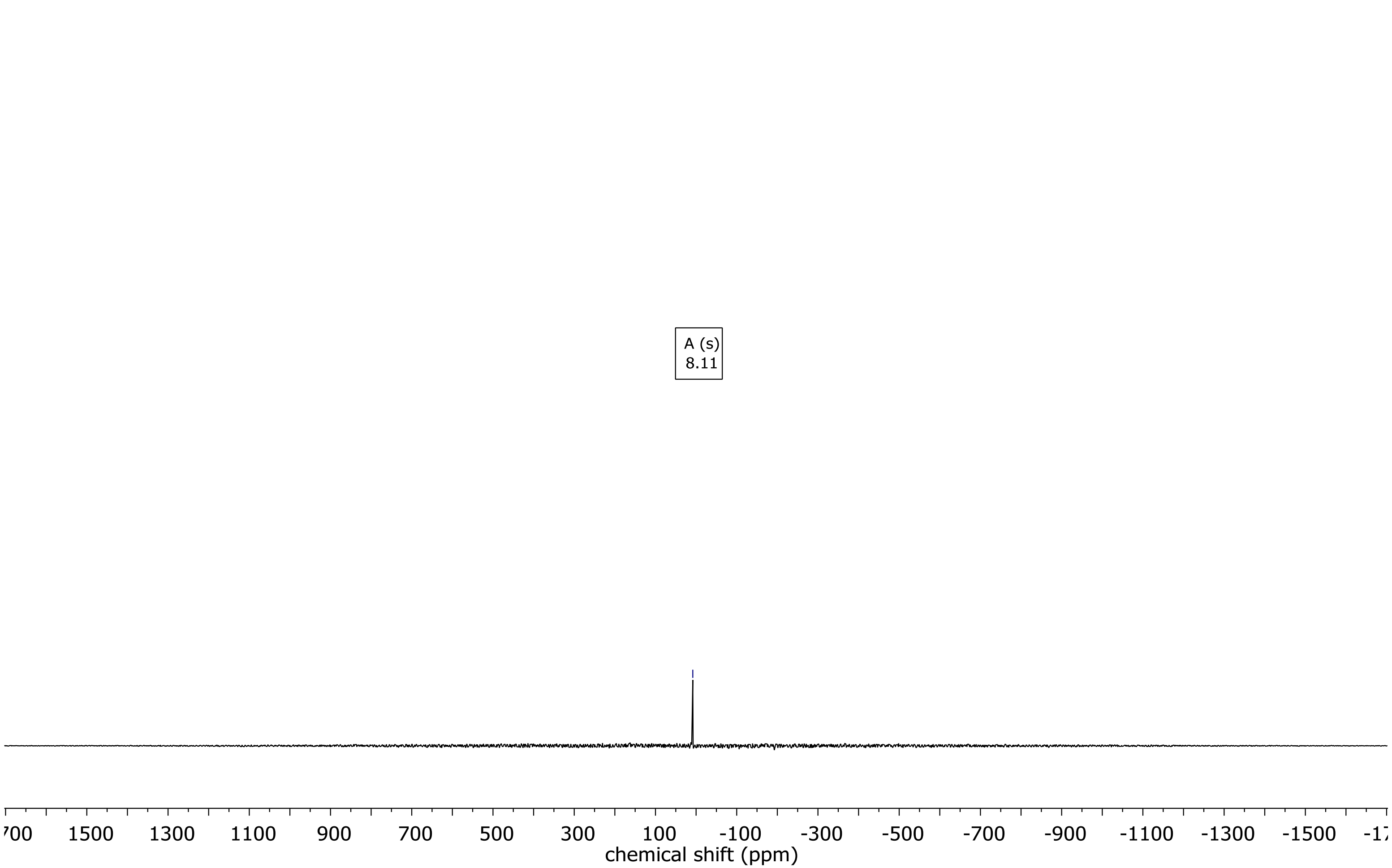

Figure S17. $^{77}Se\{^{1}H\}$ **NMR spectrum (in THF-d$_8$) of compound 3a.**

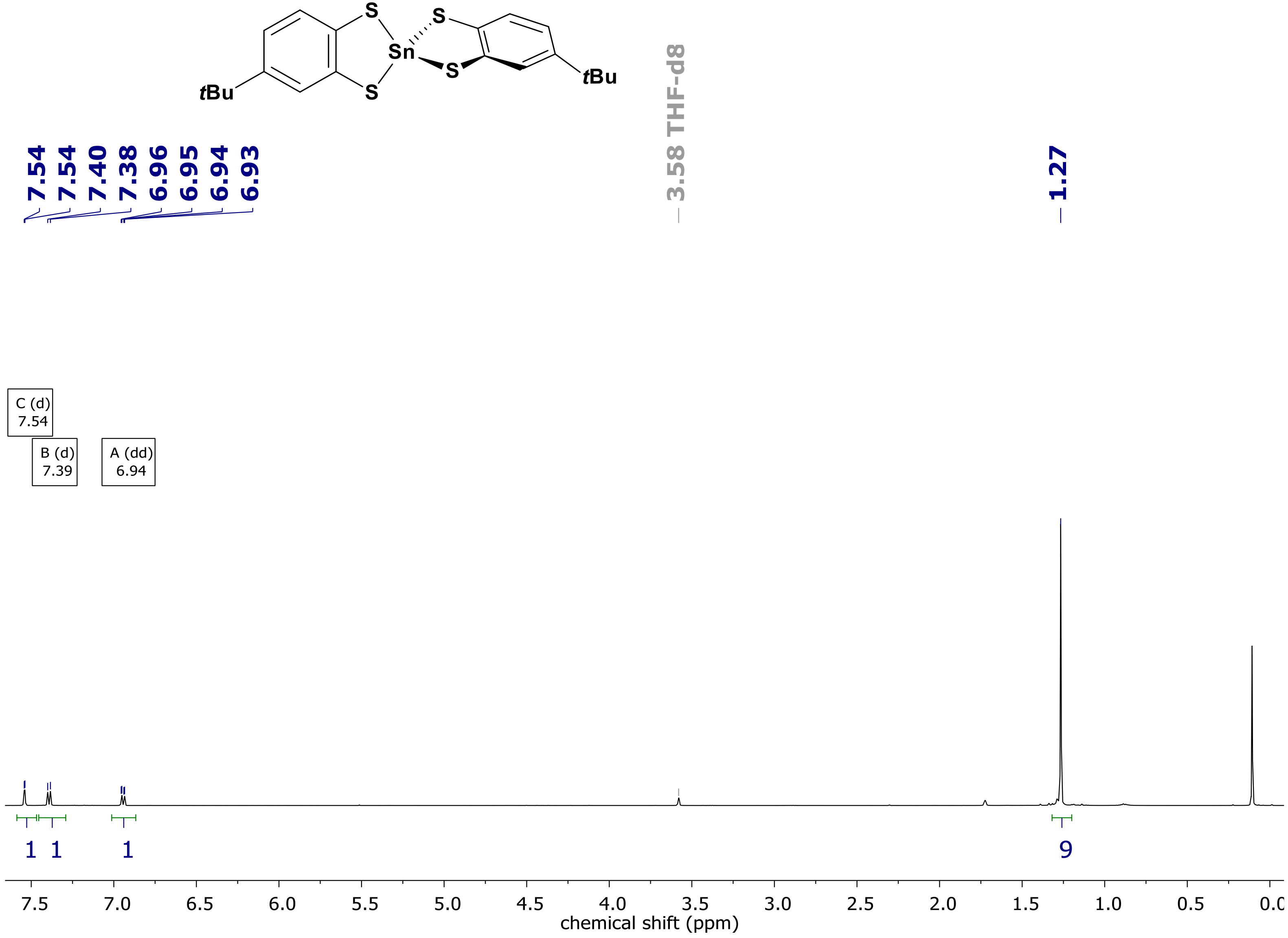

Figure S18. ^{1}H NMR spectrum (in THF-d$_8$) of compound 4a.

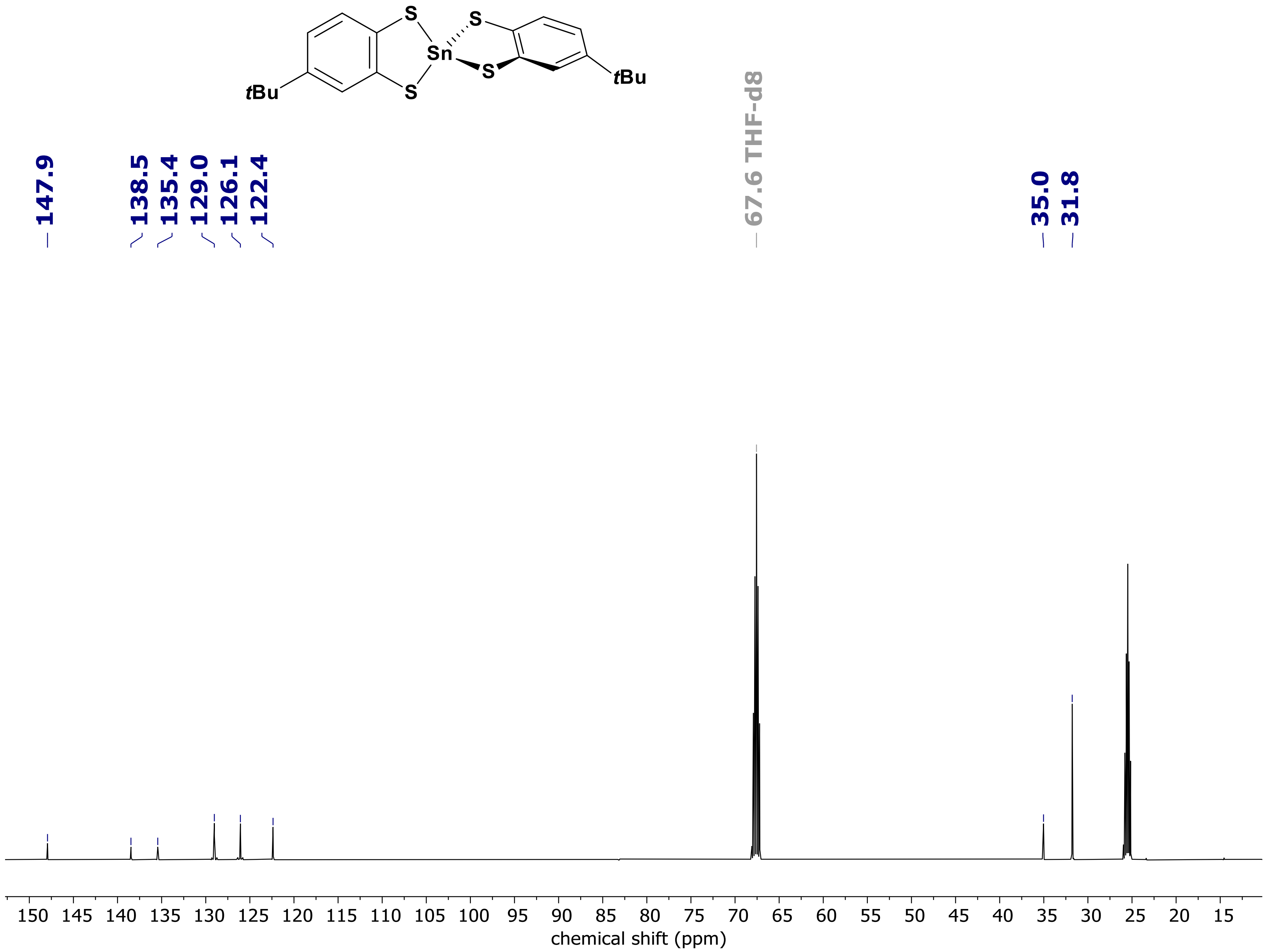

Figure S19. $^{13}C\{^{1}H\}$ **NMR spectrum (in THF-d_8) of compound 4a.**

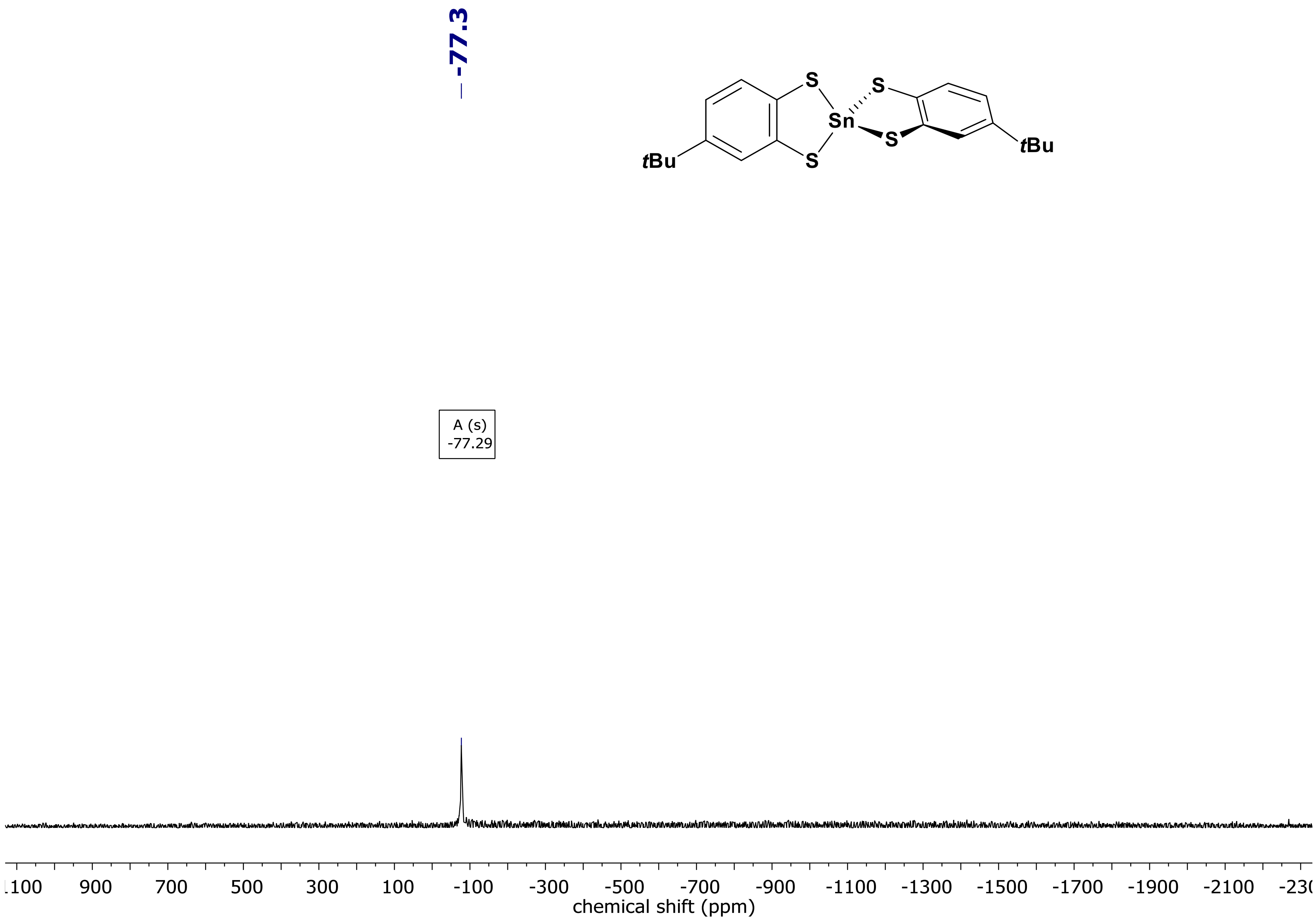

Figure S20. $^{119}Sn\{^1H\}$ **NMR spectrum (in THF-d$_8$) of compound 4a.**

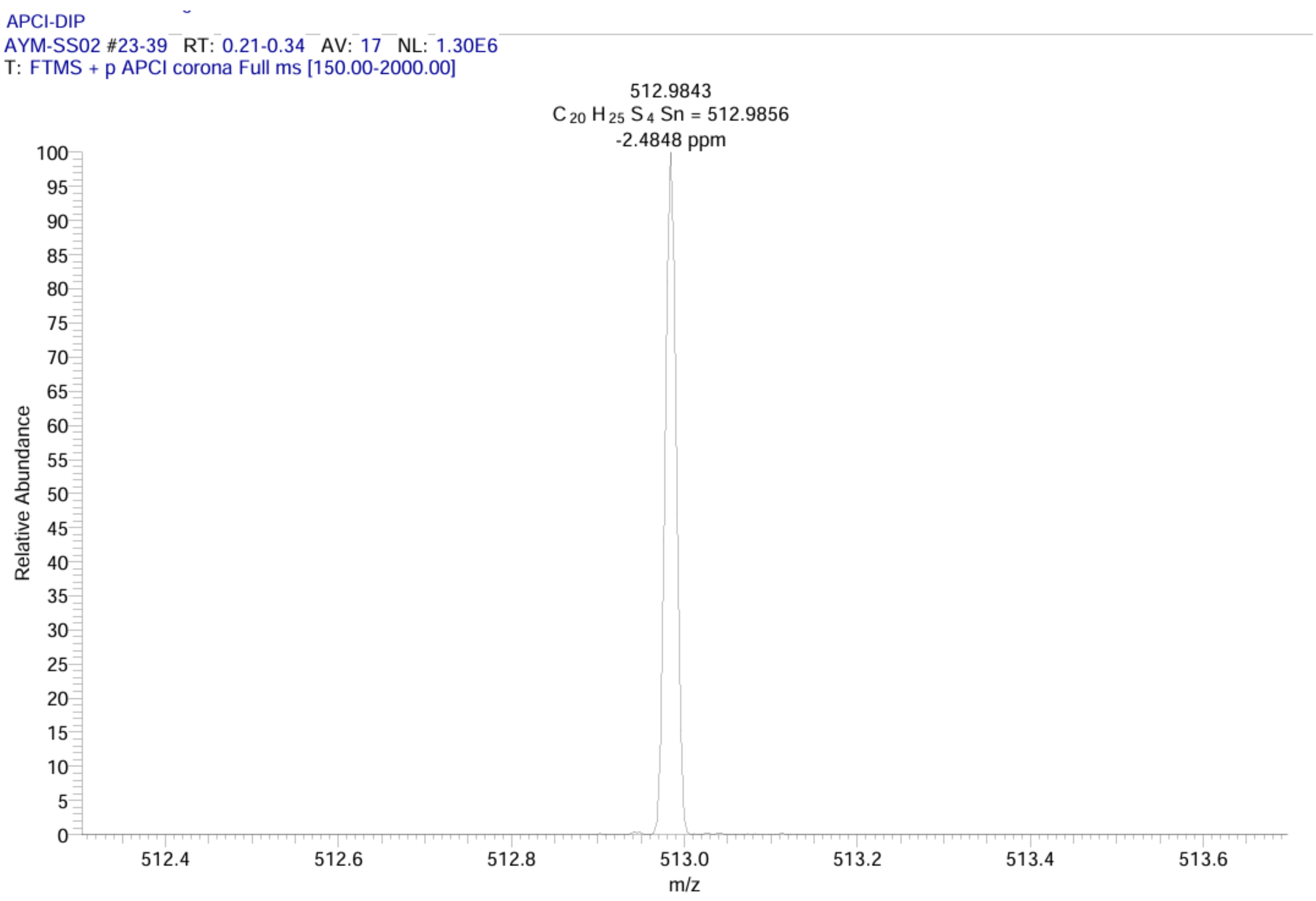

Figure S21. APCI-DIP-HRMS of compound 4a.

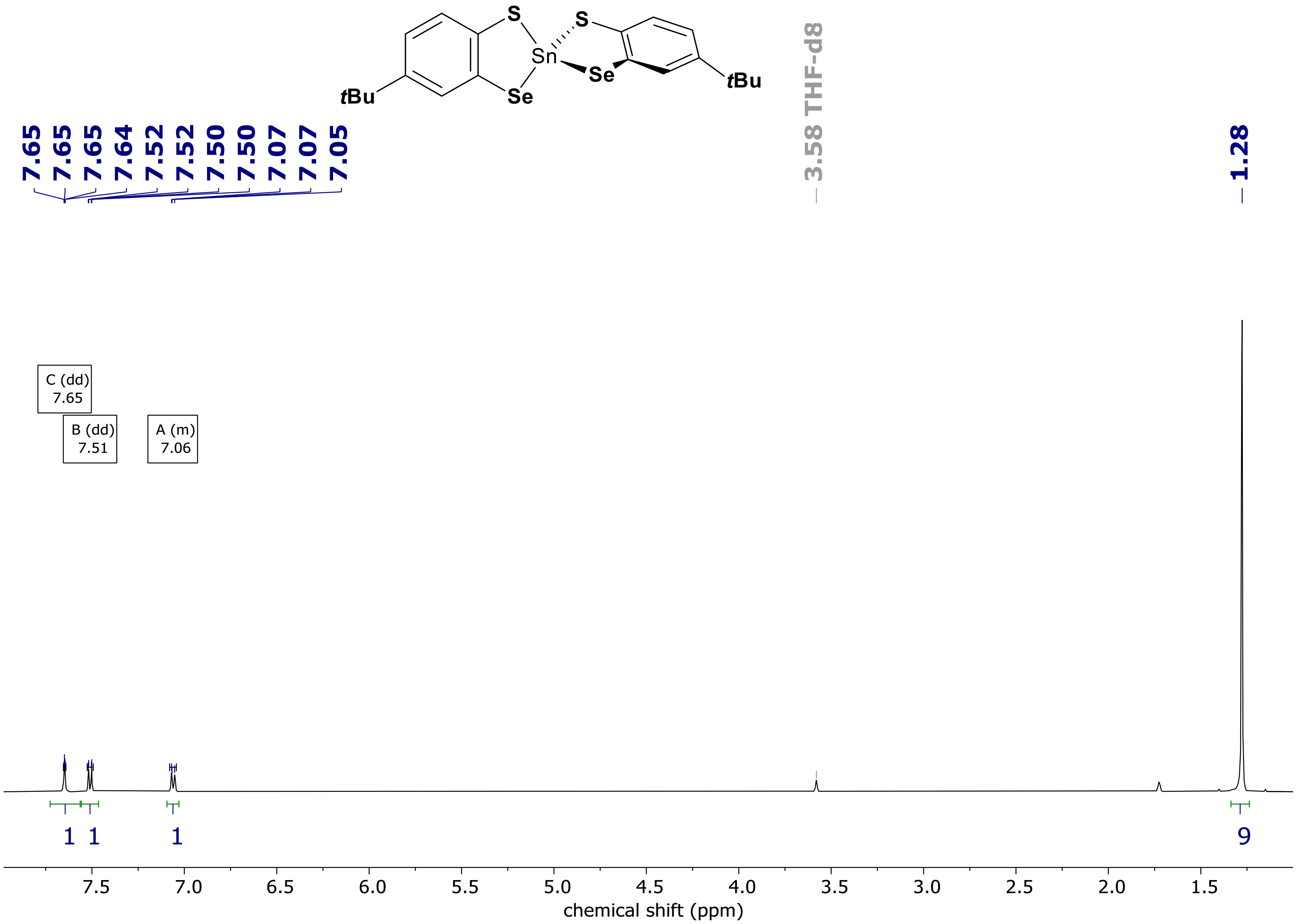

Figure S22. **^{1}H NMR spectrum (in THF-d_8) of compound 5a.**

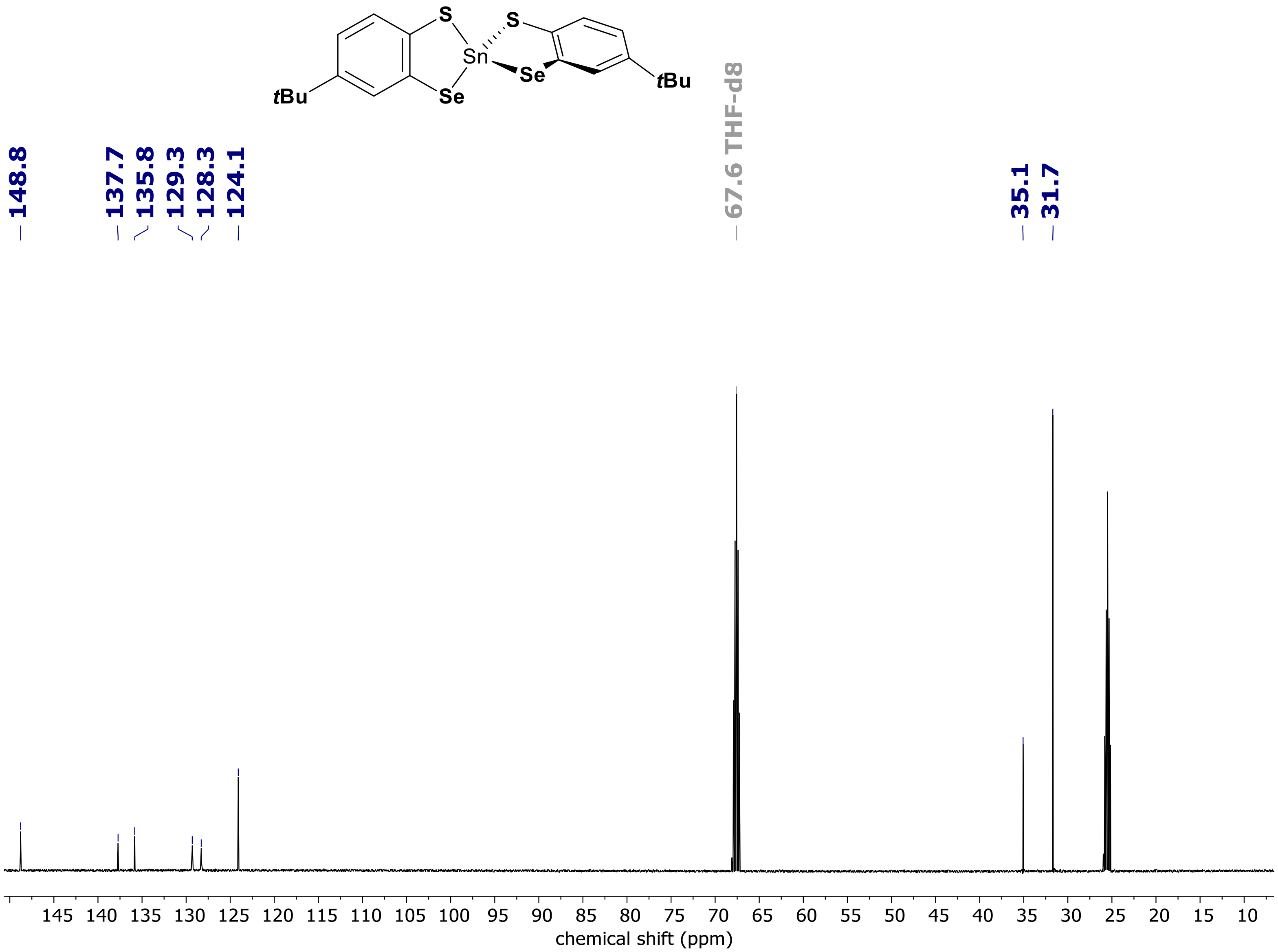

Figure S23. ^{31}C{^{1}H} **NMR spectrum (in THF-d$_8$) of compound 5a.**

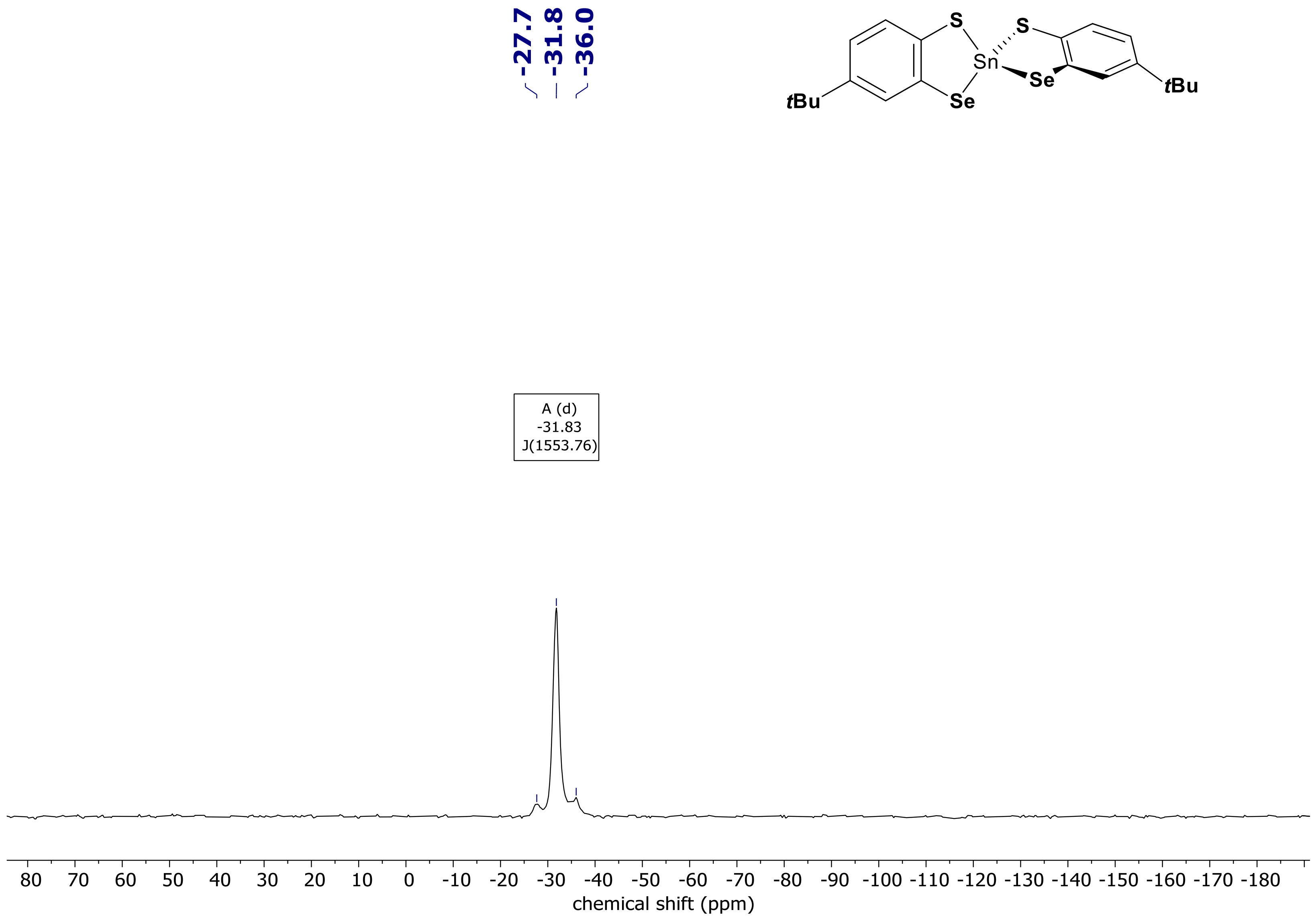

Figure S24. $^{119}Sn\{^1H\}$ **NMR spectrum (in THF-d_8) of compound 5a.**

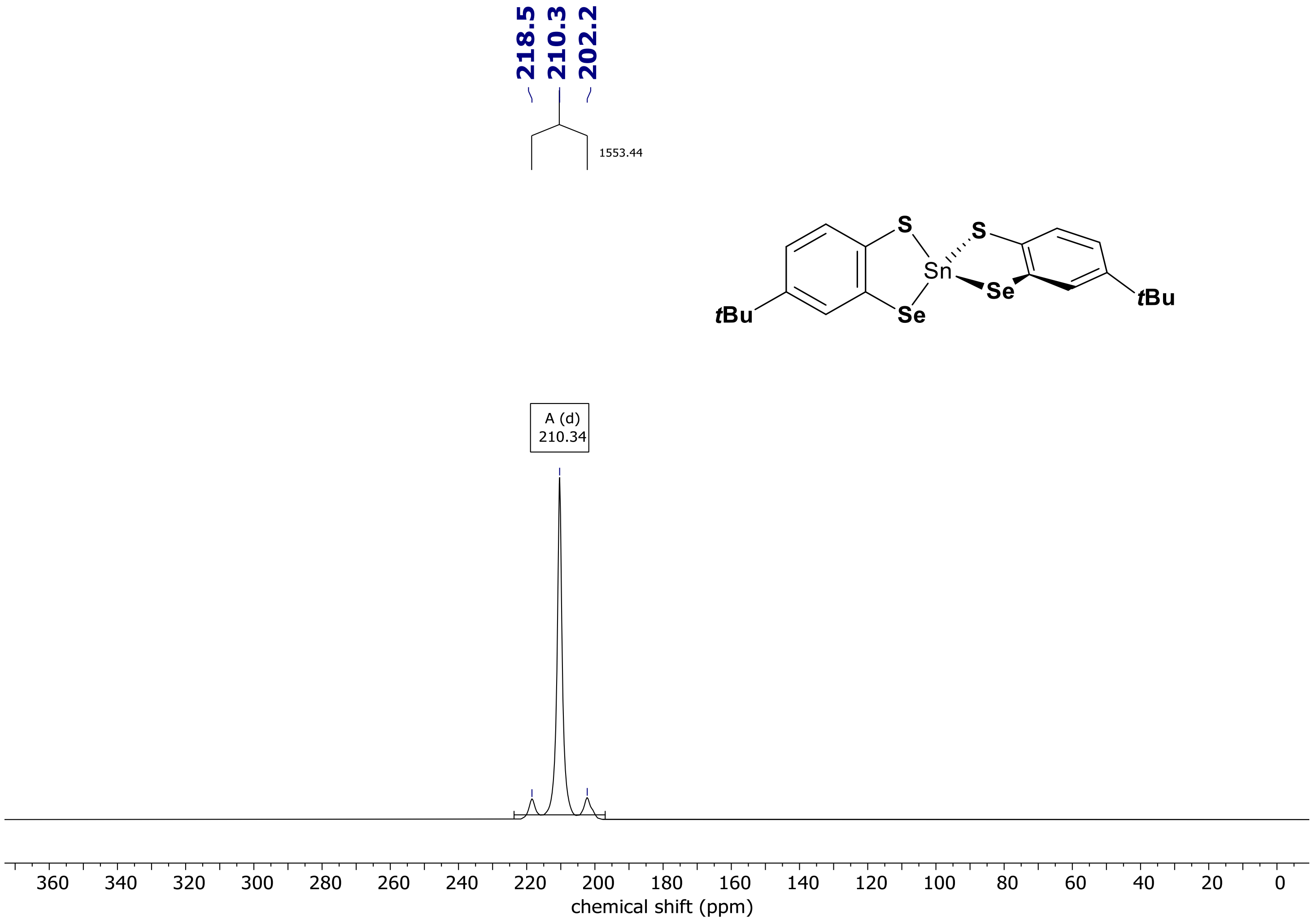

Figure S25. ^{77}Se NMR spectrum (in THF-d_8) of compound 5a.

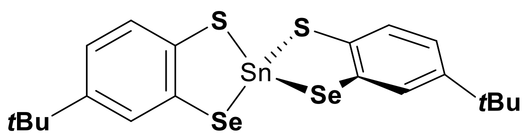

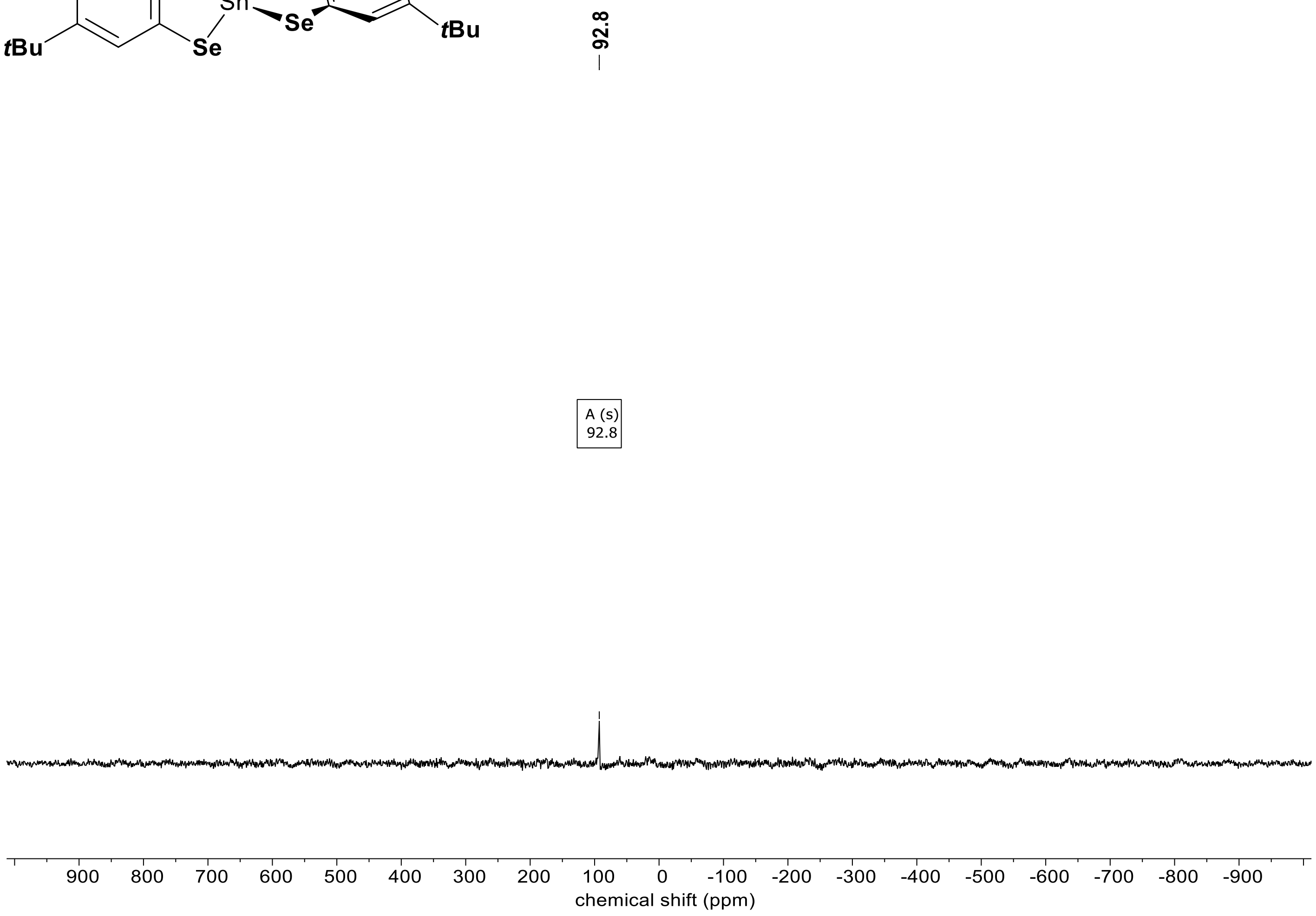

Figure S26. ^{119}**Sn{**^{1}**H} NMR spectrum (in Tol-d**$_8$**) of compound 5a.**

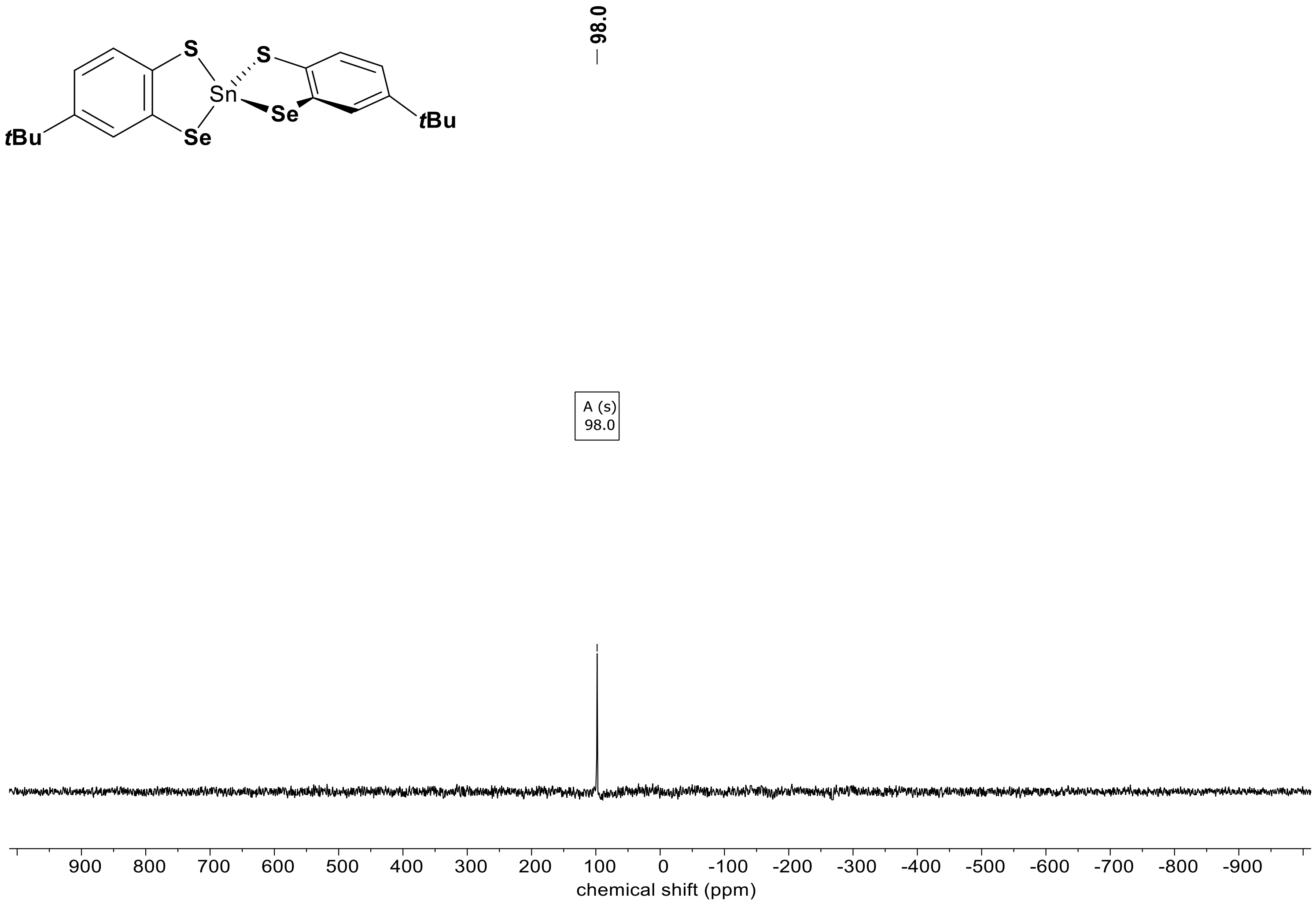

Figure S27. $^{119}Sn\{^{1}H\}$ **NMR spectrum (in C_6D_{12}) of compound 5a.**

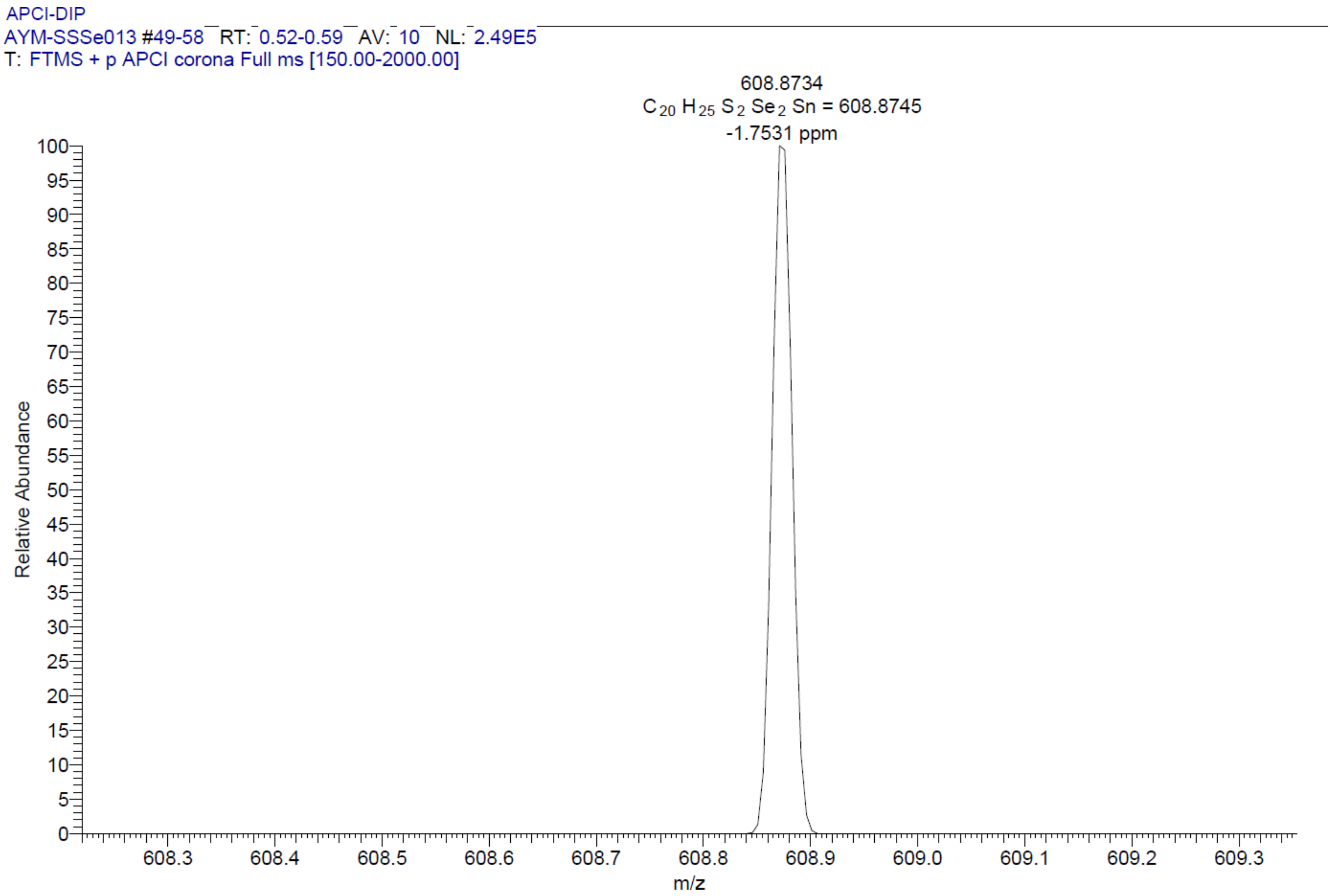

Figure S28. APCI-DIP-HRMS of compound 5a.

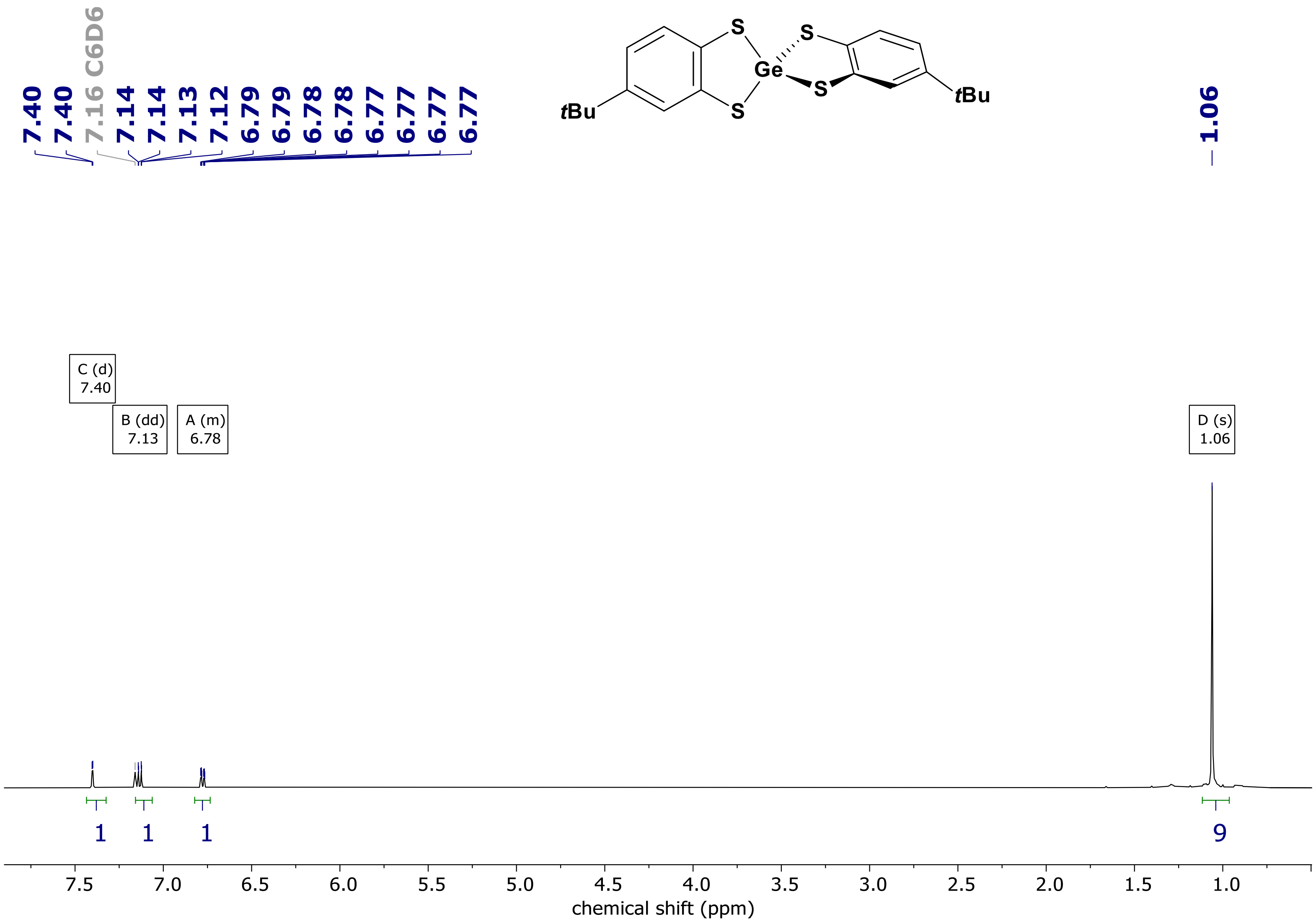

Figure S29. ^{1}**H NMR spectrum (in** C_6D_6**) of compound 4b.**

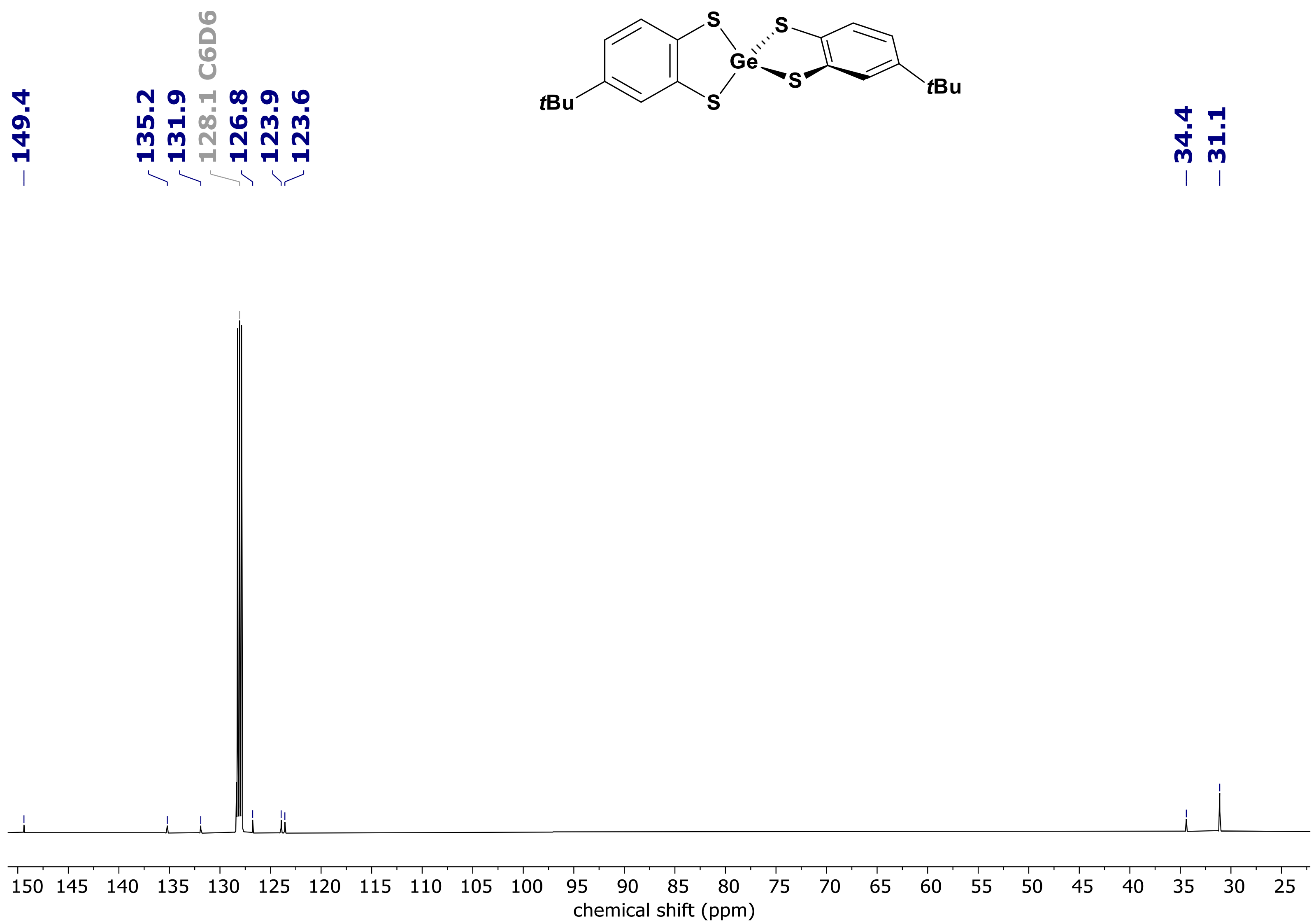

Figure S30. $^{13}C\{H\}$ **NMR spectrum (in** C_6D_6**) of compound 4b.**

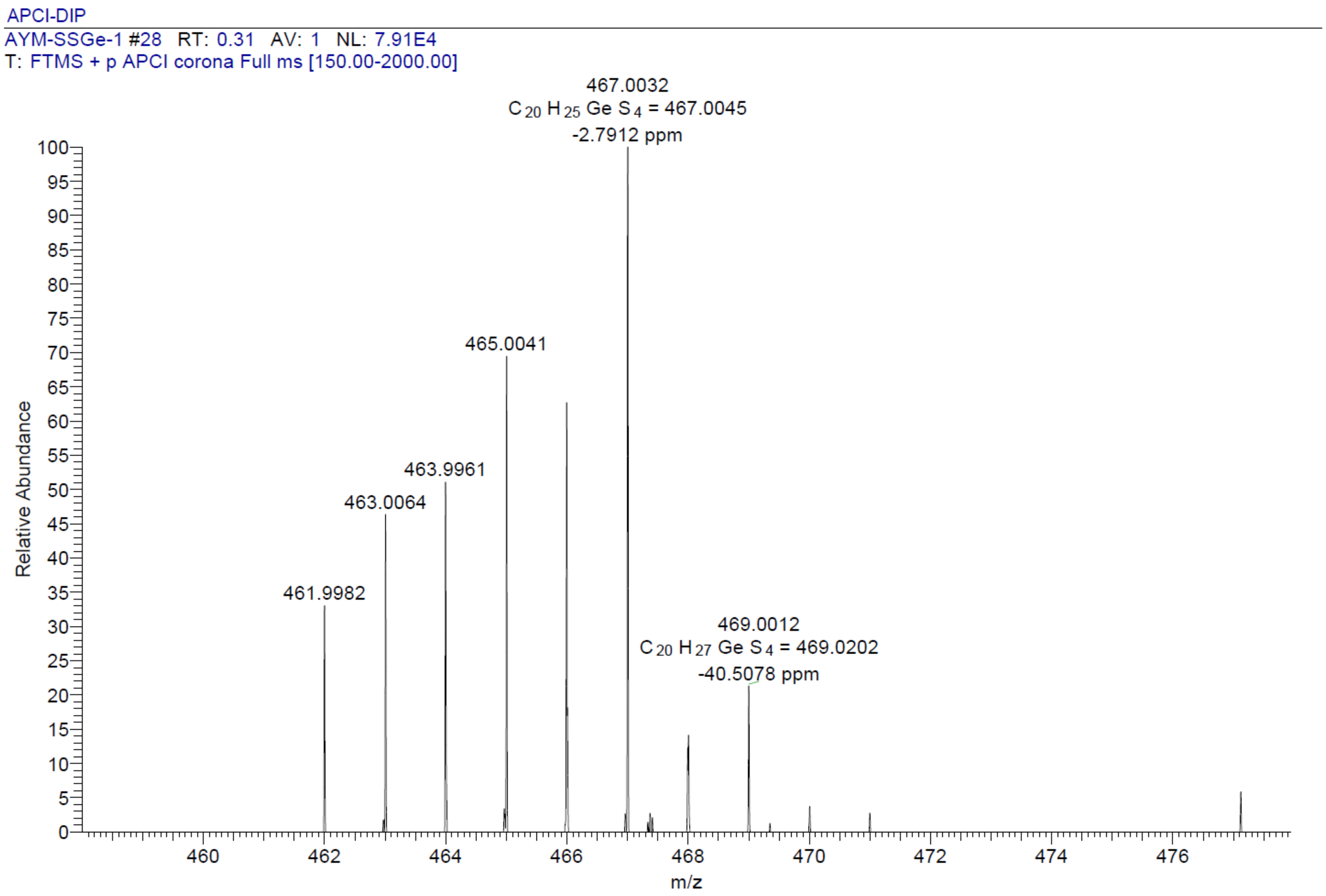

Figure S31. APCI-DIP-HRMS of compound 4b.

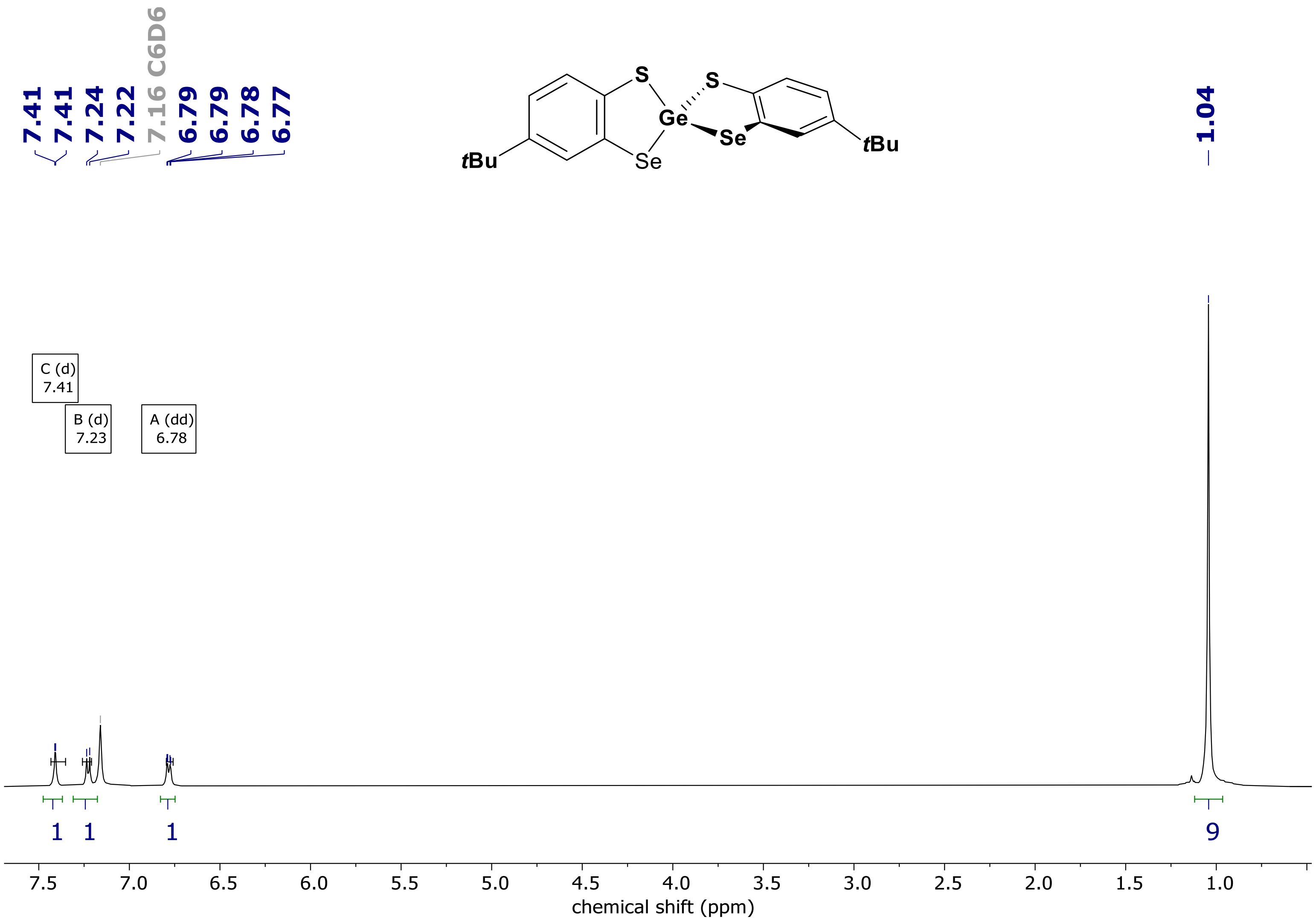

Figure S32. **^{1}H NMR spectrum (in C_6D_6) of compound 5b.**

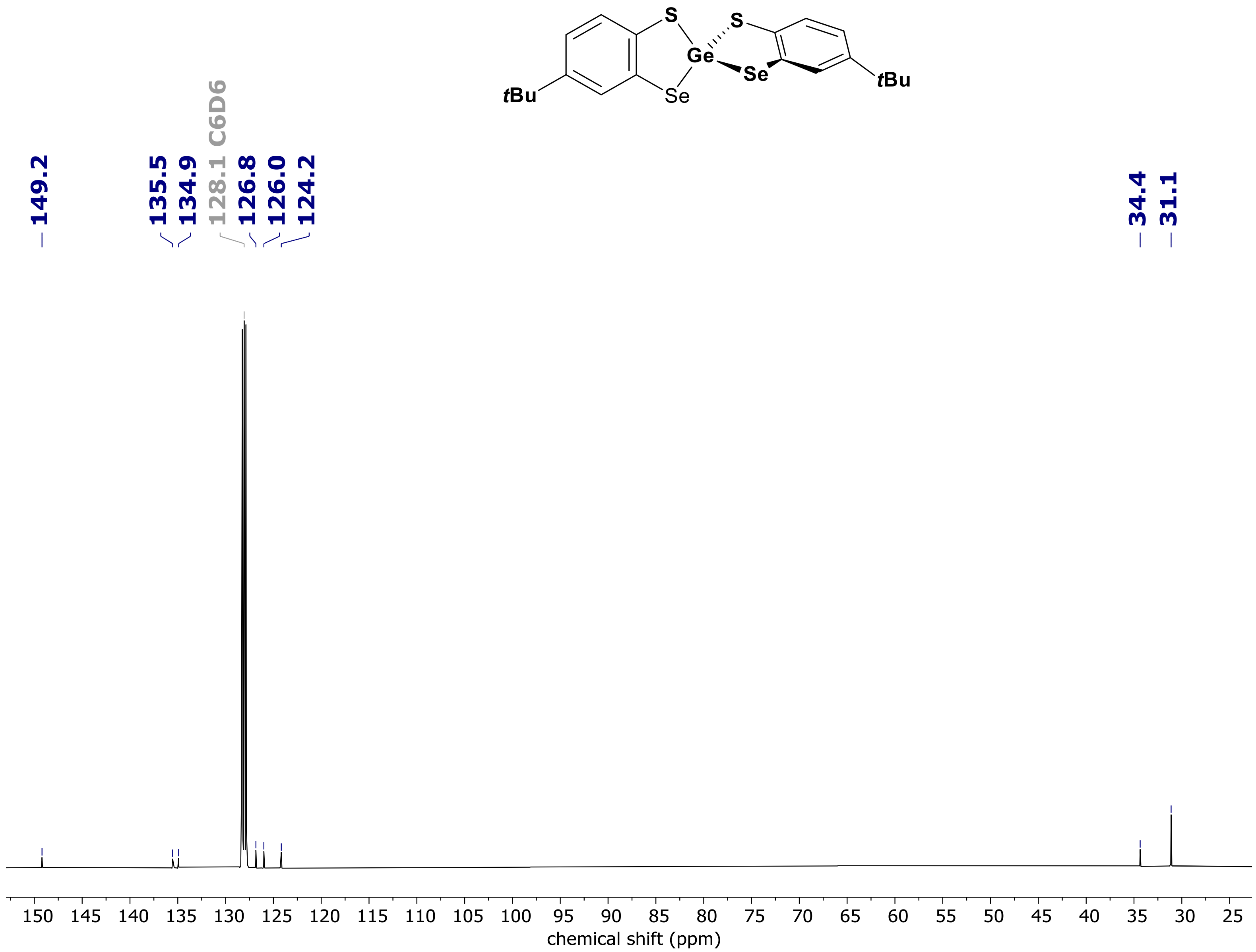

Figure S33. $^{13}C\{^{1}H\}$ **NMR spectrum (in C_6D_6) of compound 5b.**

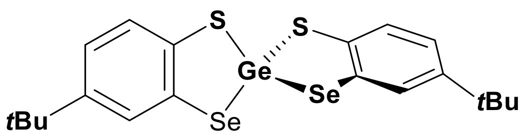

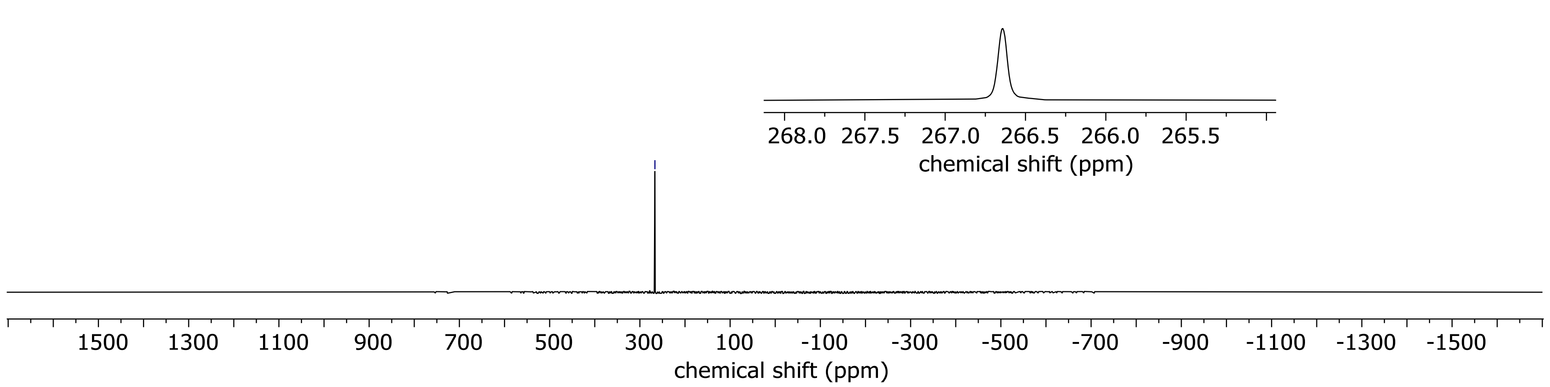

Figure S34. $^{77}Se\{^{1}H\}$ **NMR spectrum (in** C_6D_6**) of compound 5b.**

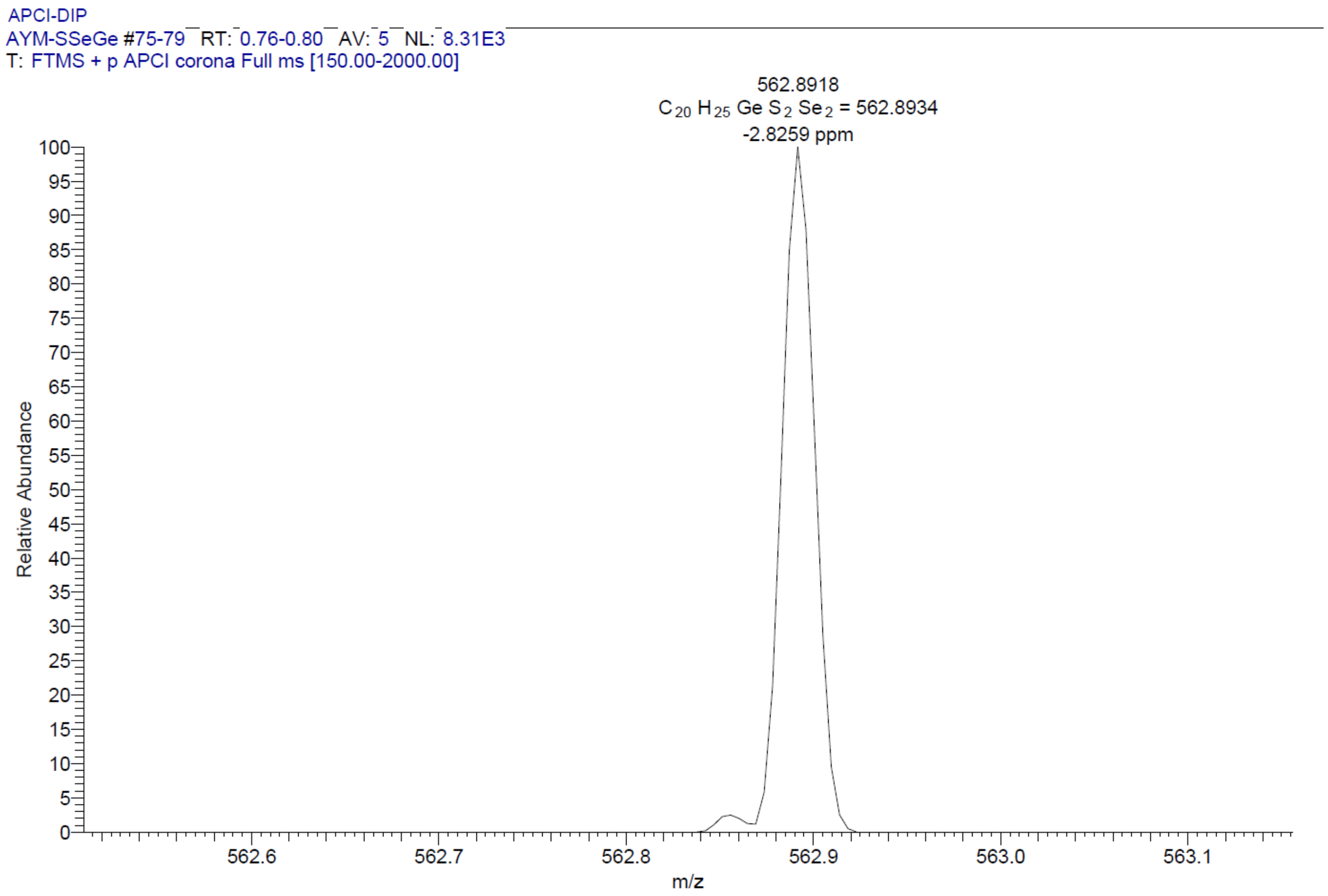

Figure S35. APCI-DIP-HRMS of compound 5b.

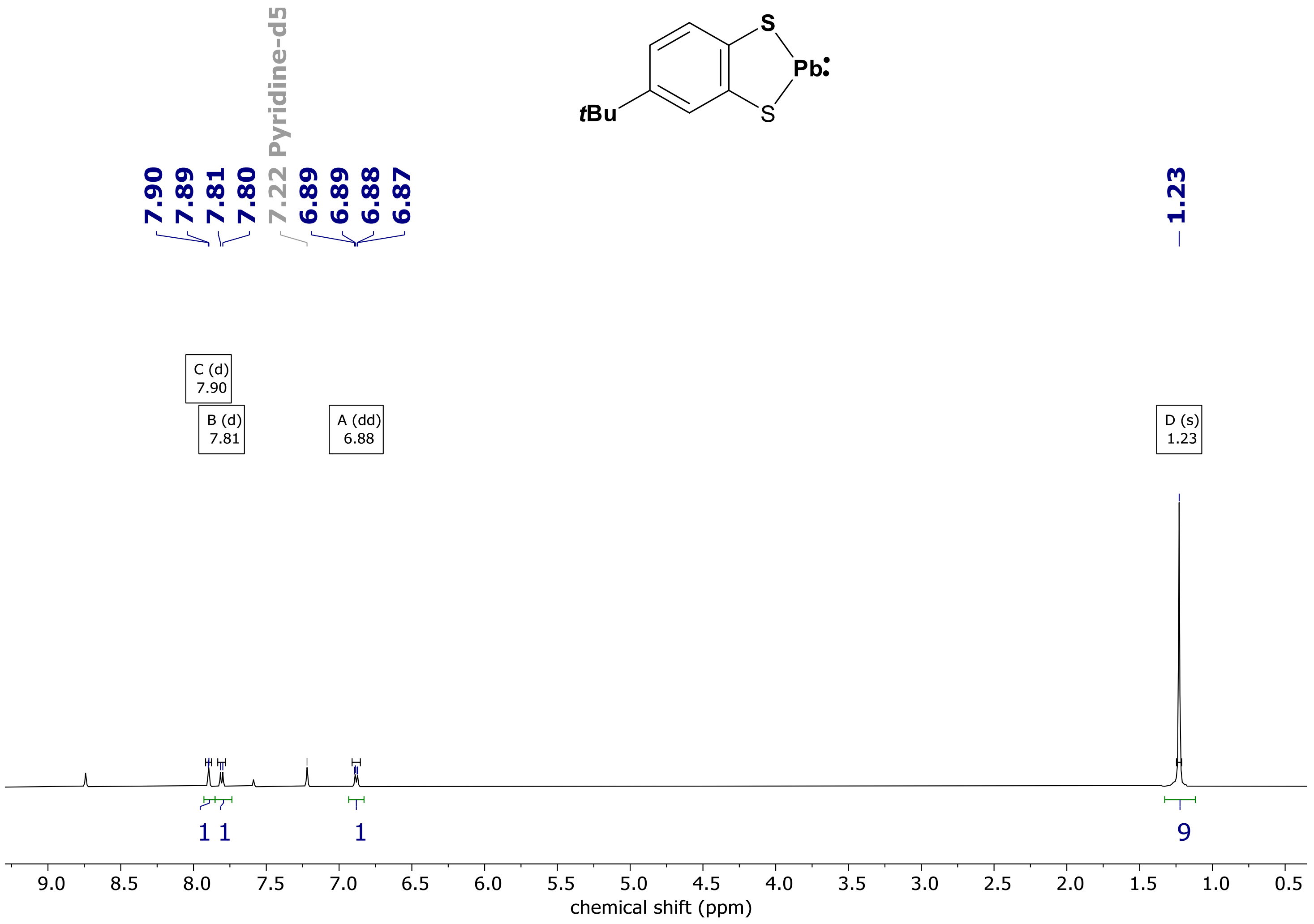

Figure S36. ^{1}H NMR spectrum (in C_5D_5N) of compound 6a.

6a

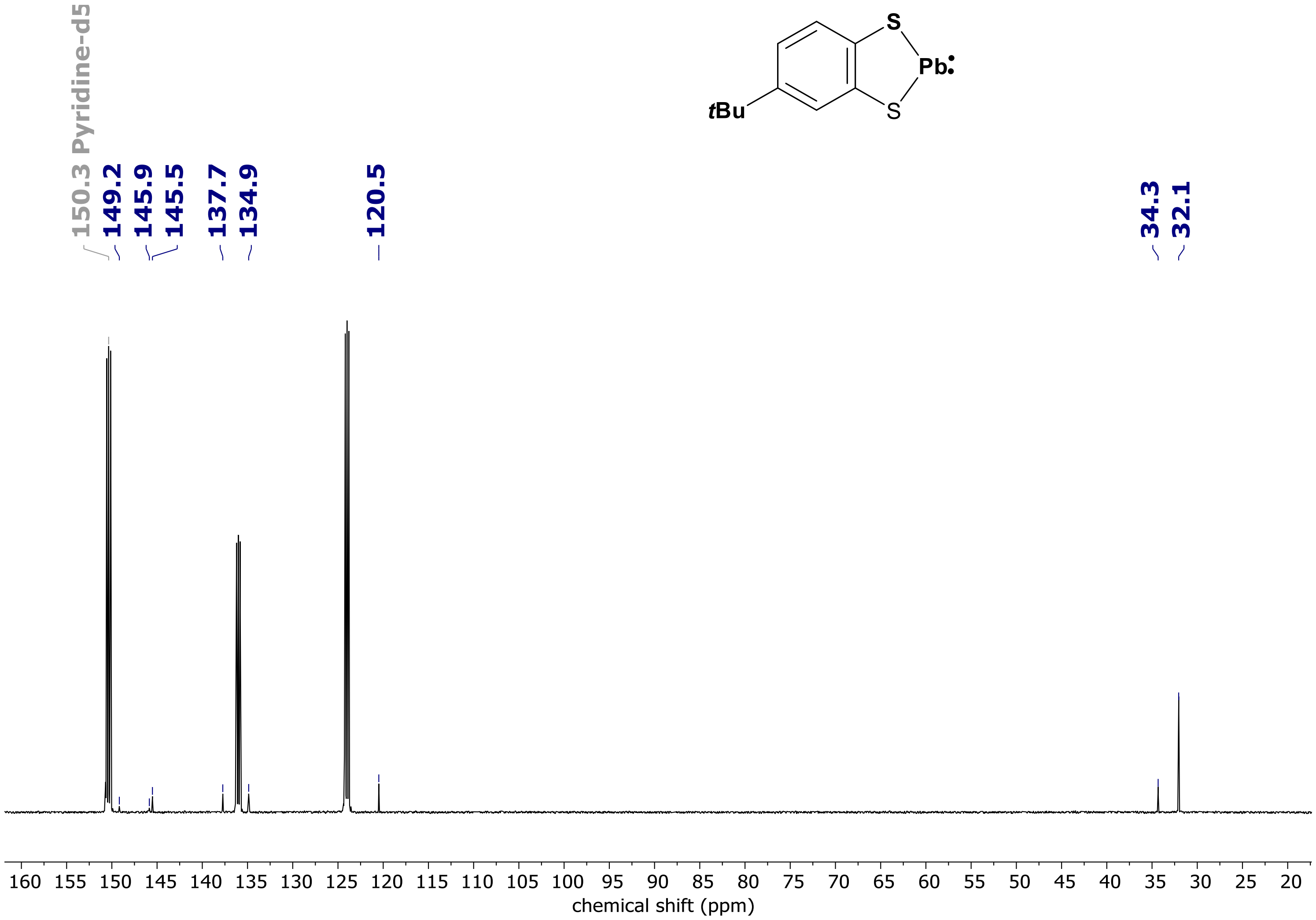

Figure S37. $^{13}C\{^{1}H\}$ **NMR spectrum (in C_5D_5N) of compound 6a.**

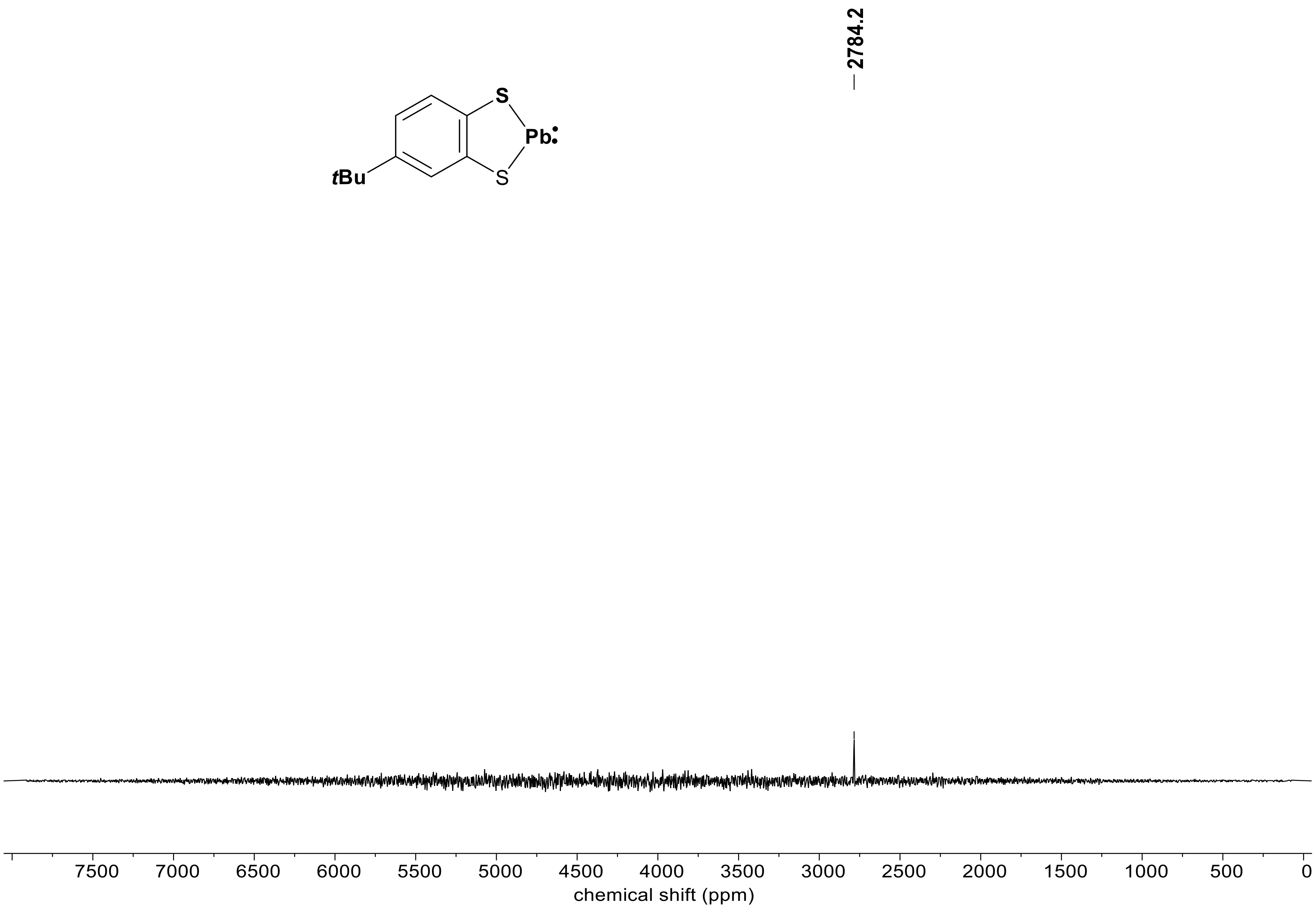

Figure S38. ^{207}**Pb NMR spectrum (in** C_5D_5N**) of compound 6a.**

Table S1. ^{1}H DOSY-ECC-MW estimation of 6a in DMSO-d_6 at 25 °C.

Tetrametylsilane was used as internal reference with logDref, fix (TMS)[11]= – 9.2068. The accuracy of the ECC^{DMSO-d_6} (DSE) is in the range of $MW_{dif} \leq \pm 4\%$ and of ECC^{DMSO-d_6} (Merge) in the range of $MW_{dif} \leq \pm 3\%$.

**^{1}H DOSY**				**25 °C**
**Dx [m^2/s]**	2.14*e-6	**Compound**	**MW_{calc} [g/mol]**	**MW_{dif} [%]**
**$logD_x$**	-5.669			- 15 (CS)
**$logD_{x, norm}$**	- 9.6197	$C_{10}H_{12}S_2Pb$ (**6a**)	404	- 4 (DSE)
**D_{ref} (Tetramethylsilane) [m^2/s]**	5.54*e-6			- 3 (Merge)
**$logD_{ref}$ (Tetramethylsilane)**	-5.256			10 (ED)
**MW_{det} [g/mol] (CS)**	472			
**MW_{det} [g/mol] (DSE)**	420			
**MW_{det} [g/mol] (Merge)**	393			
**MW_{det} [g/mol] ED)**	367			

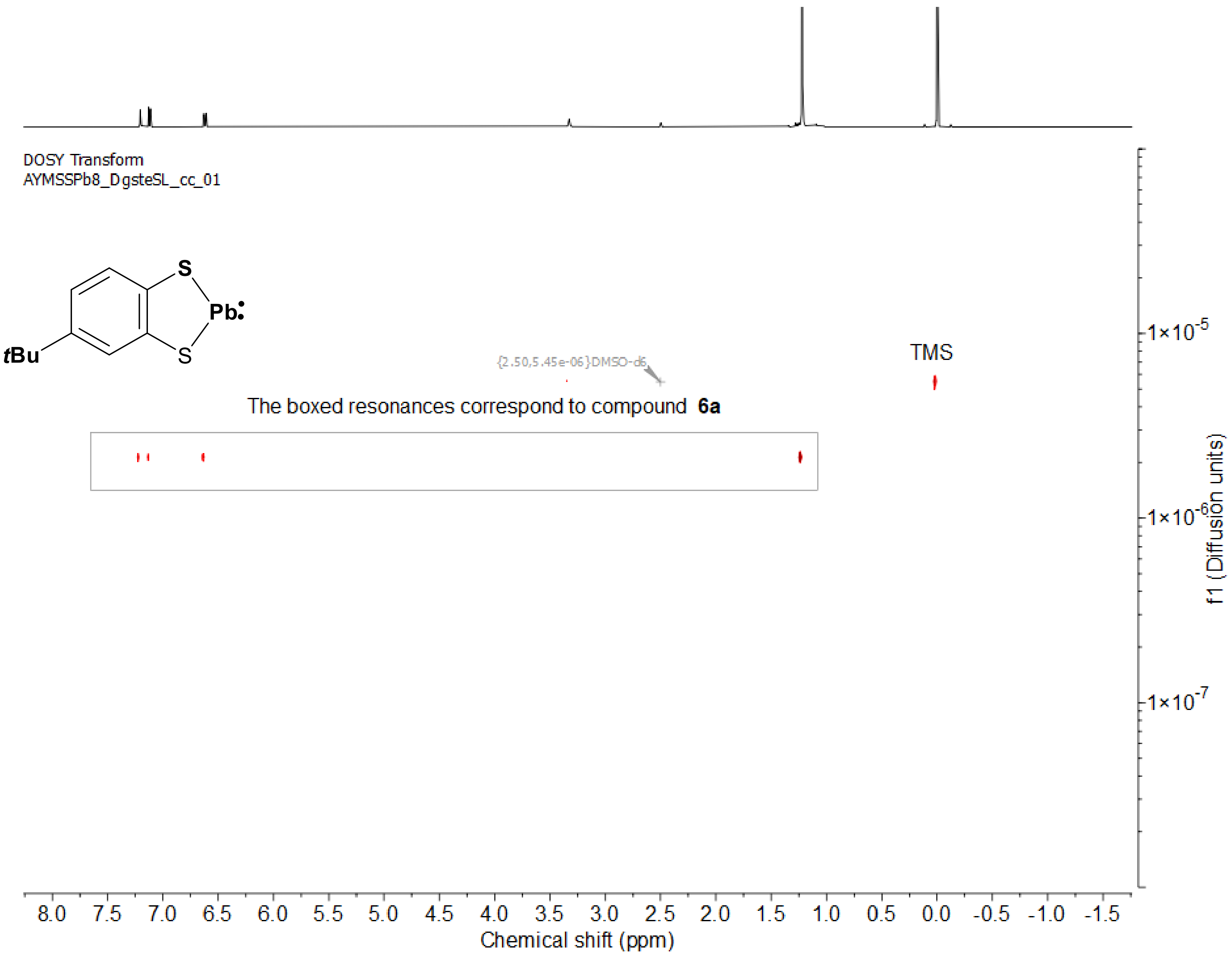

Figure S39: ^{1}H DOSY spectrum of 6a in DMSO-d$_6$.
(Internal reference: Tetramethylsilane)

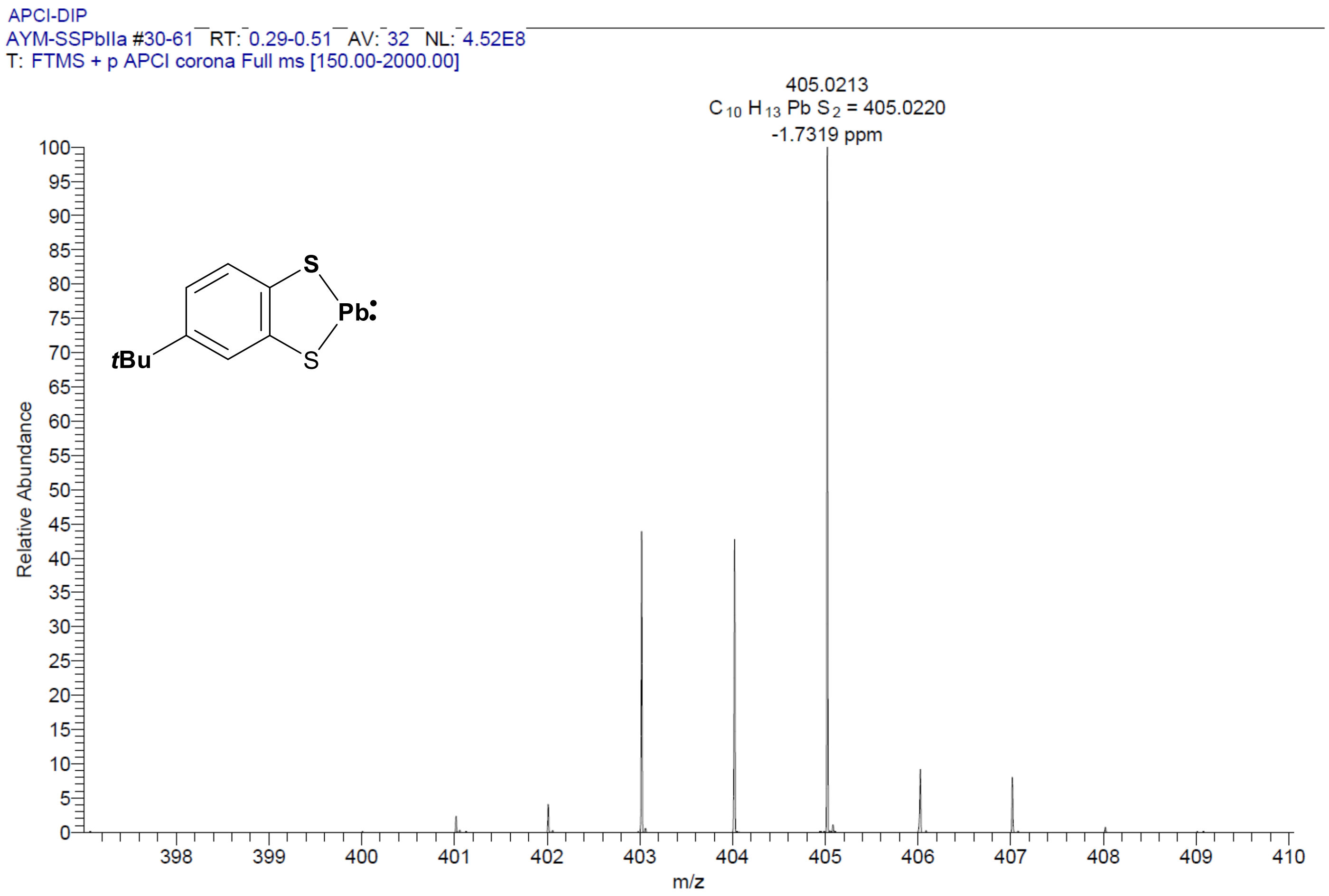

Figure S40. APCI-DIP-HRMS of compound 6a [M+H]$^+$.

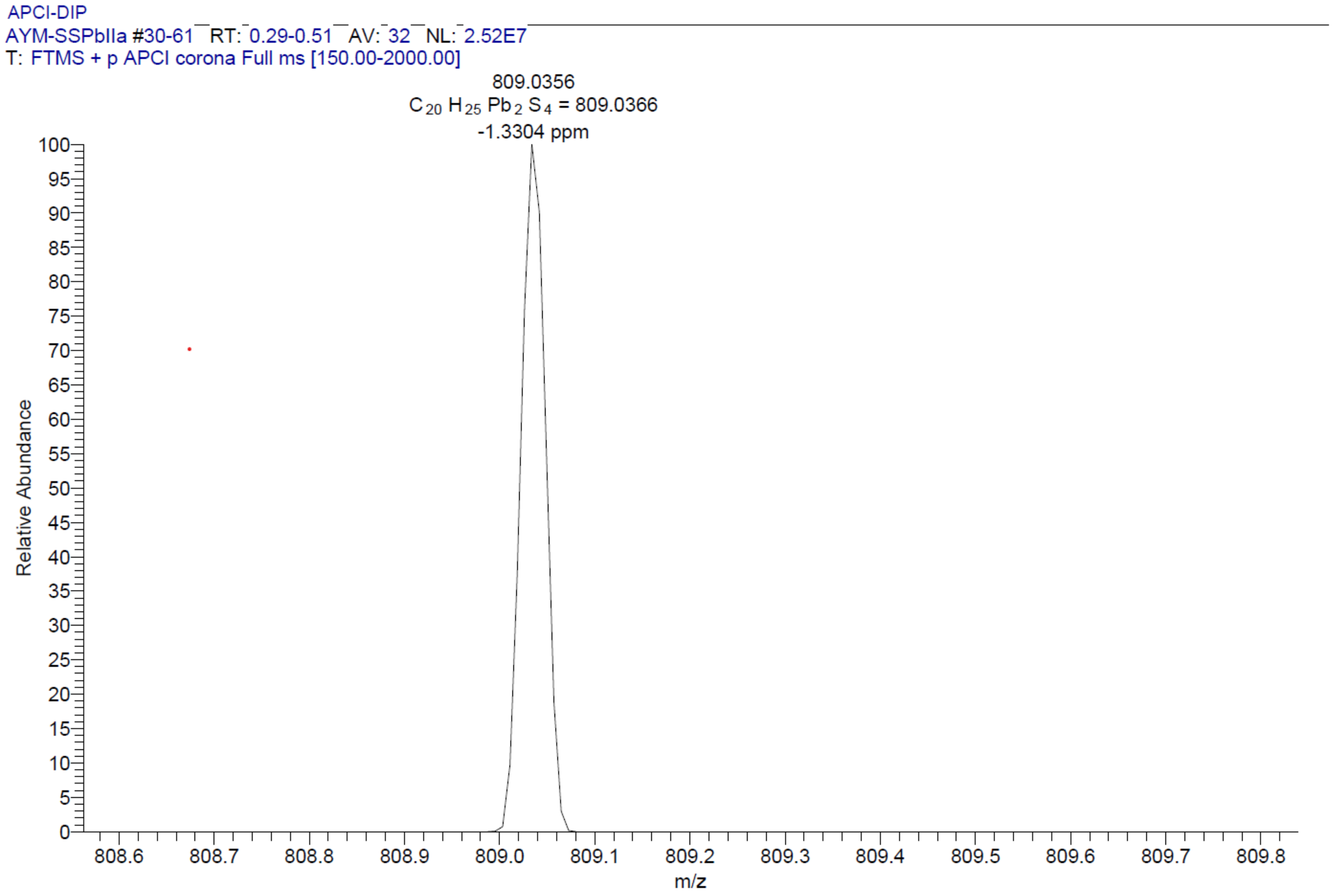

Figure S41. APCI-DIP-HRMS of compound 6a [2M+H]$^+$.

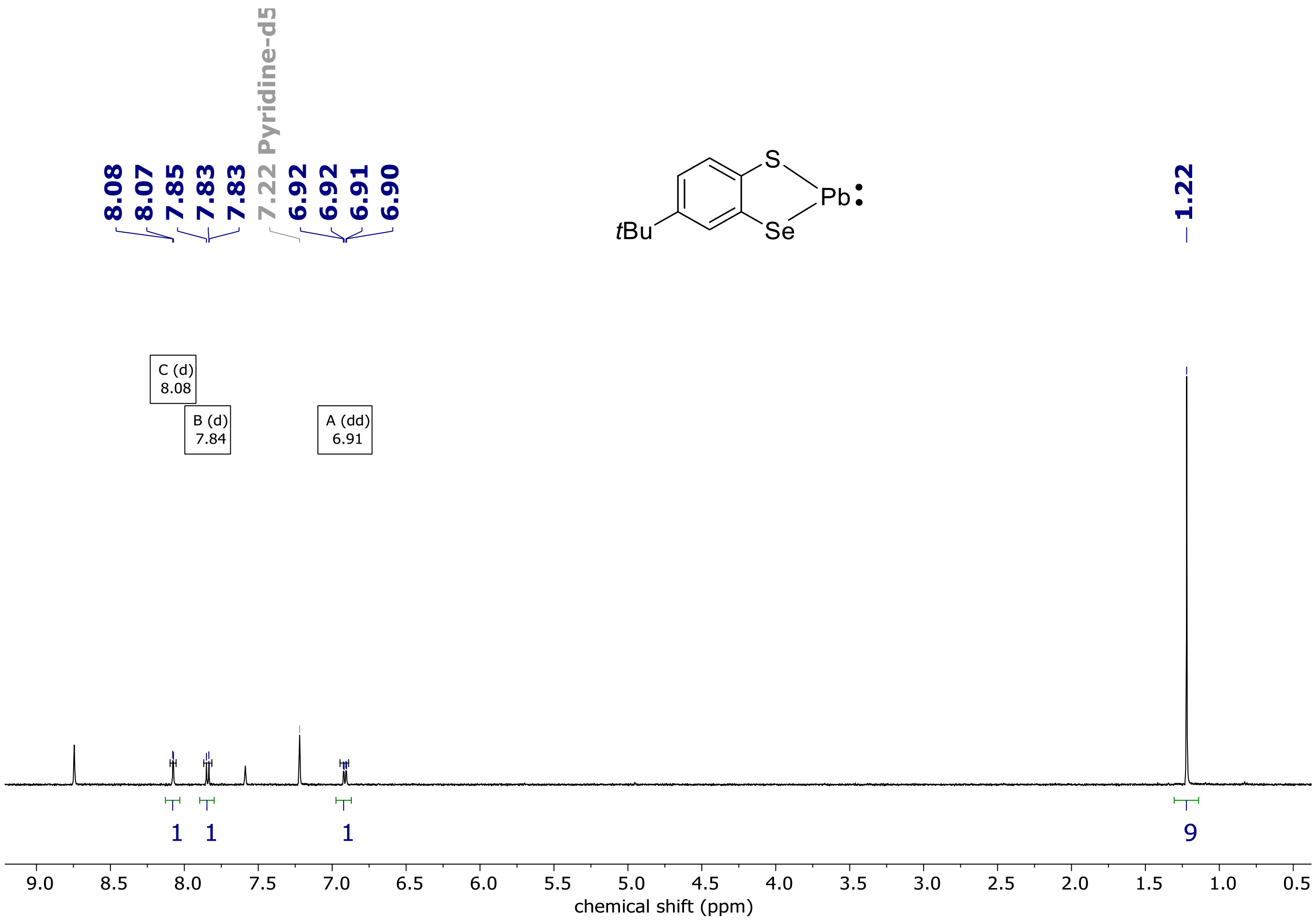

Figure S42. ^{1}H **NMR spectrum (in** C_5D_5N**) of compound 7a.**

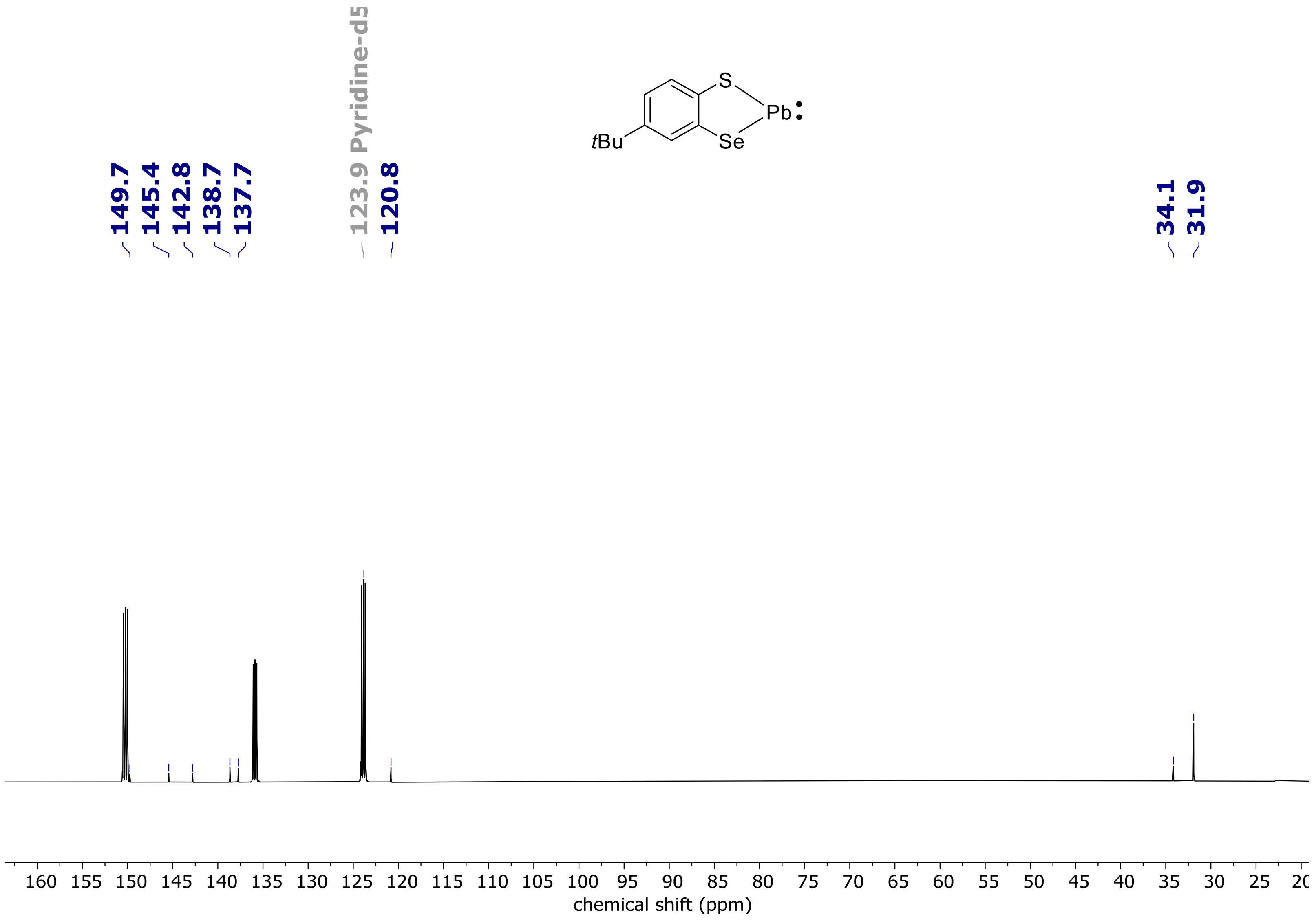

Figure S43. $^{13}C\{^1H\}$ **NMR spectrum (in** C_5D_5N**) of compound 7a.**

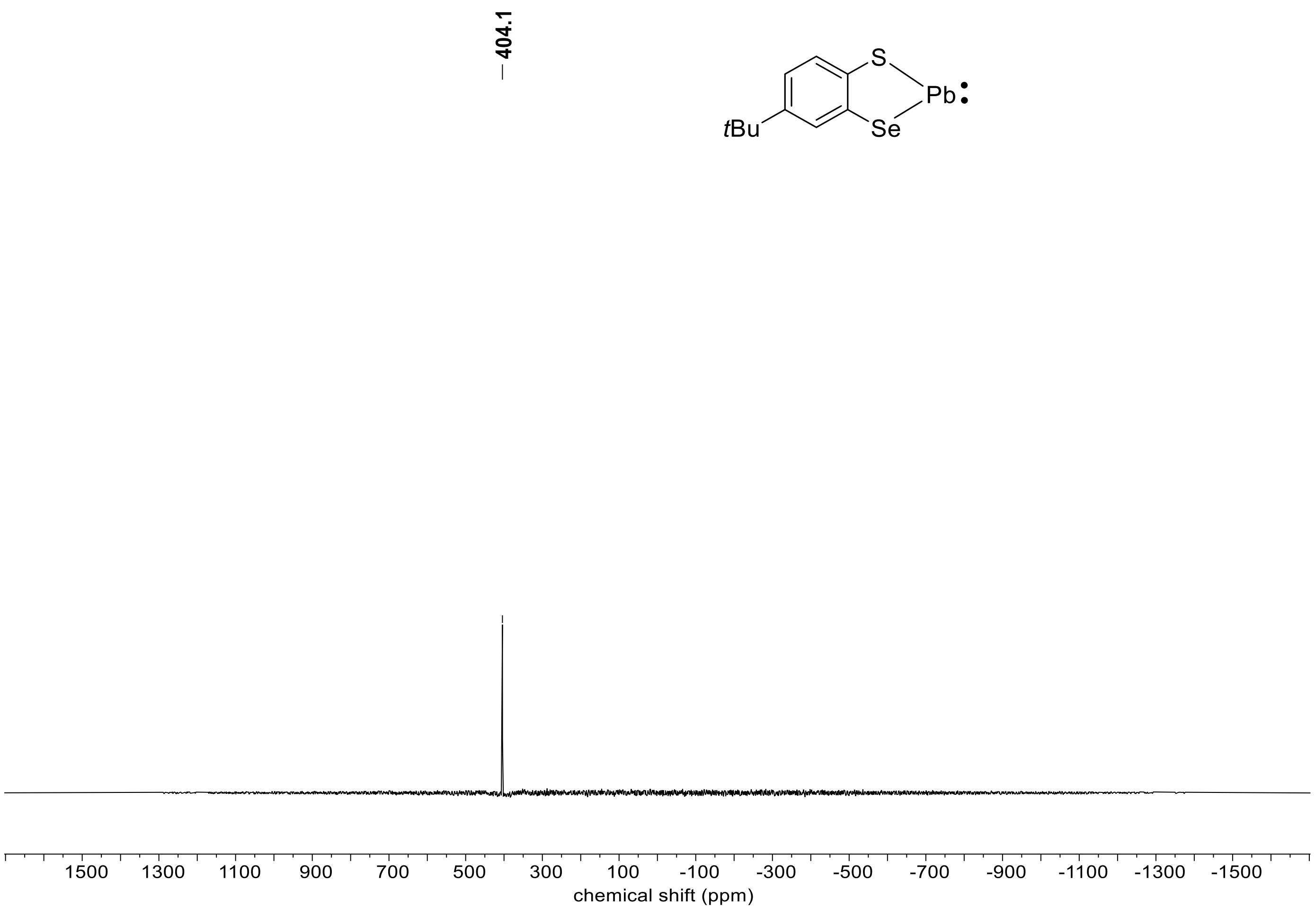

Figure S44. ^{77}Se NMR spectrum (in C_5D_5N) of compound 7a.

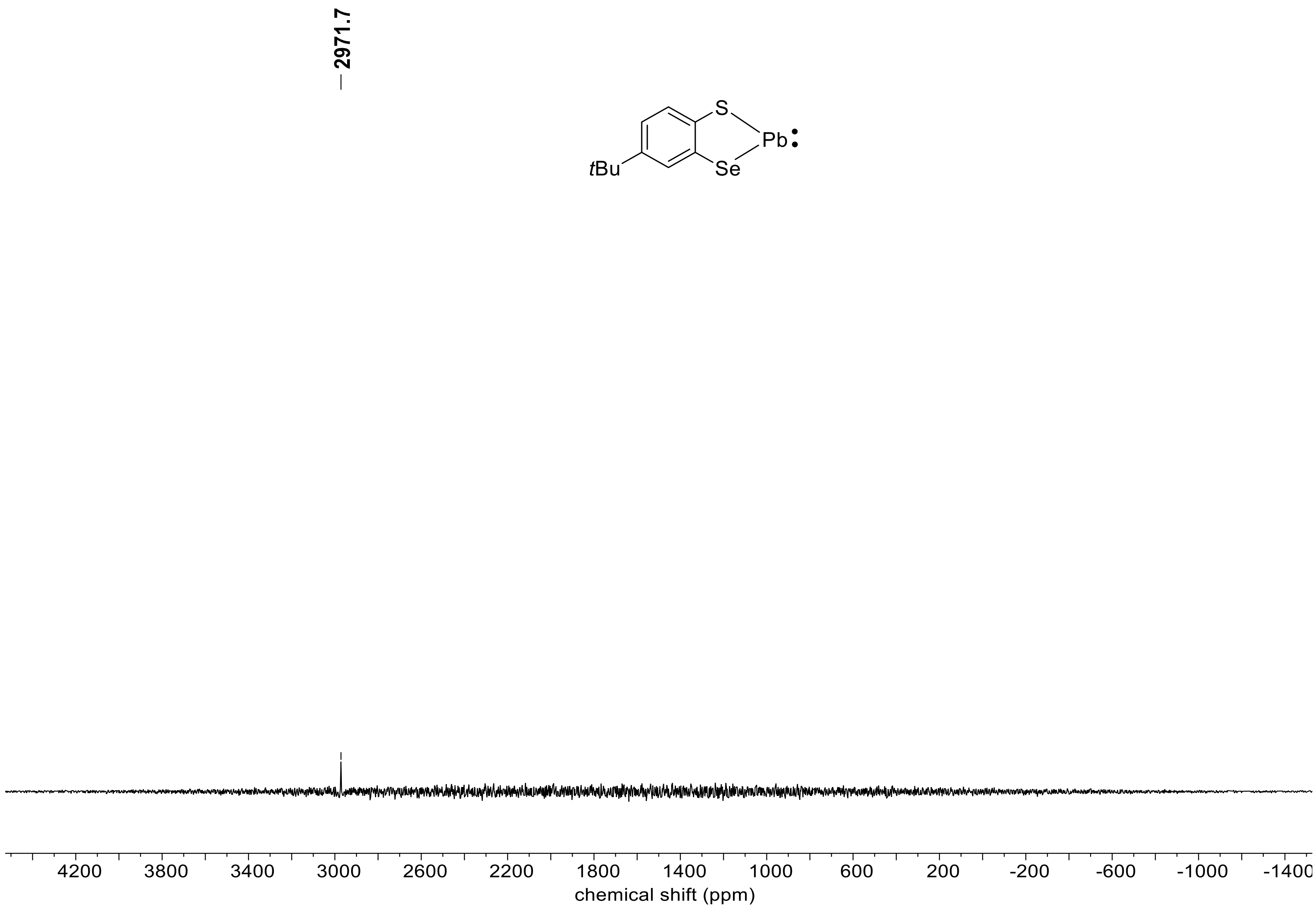

Figure S45. ^{207}**Pb NMR spectrum (in** C_5D_5N**) of compound 7a.**

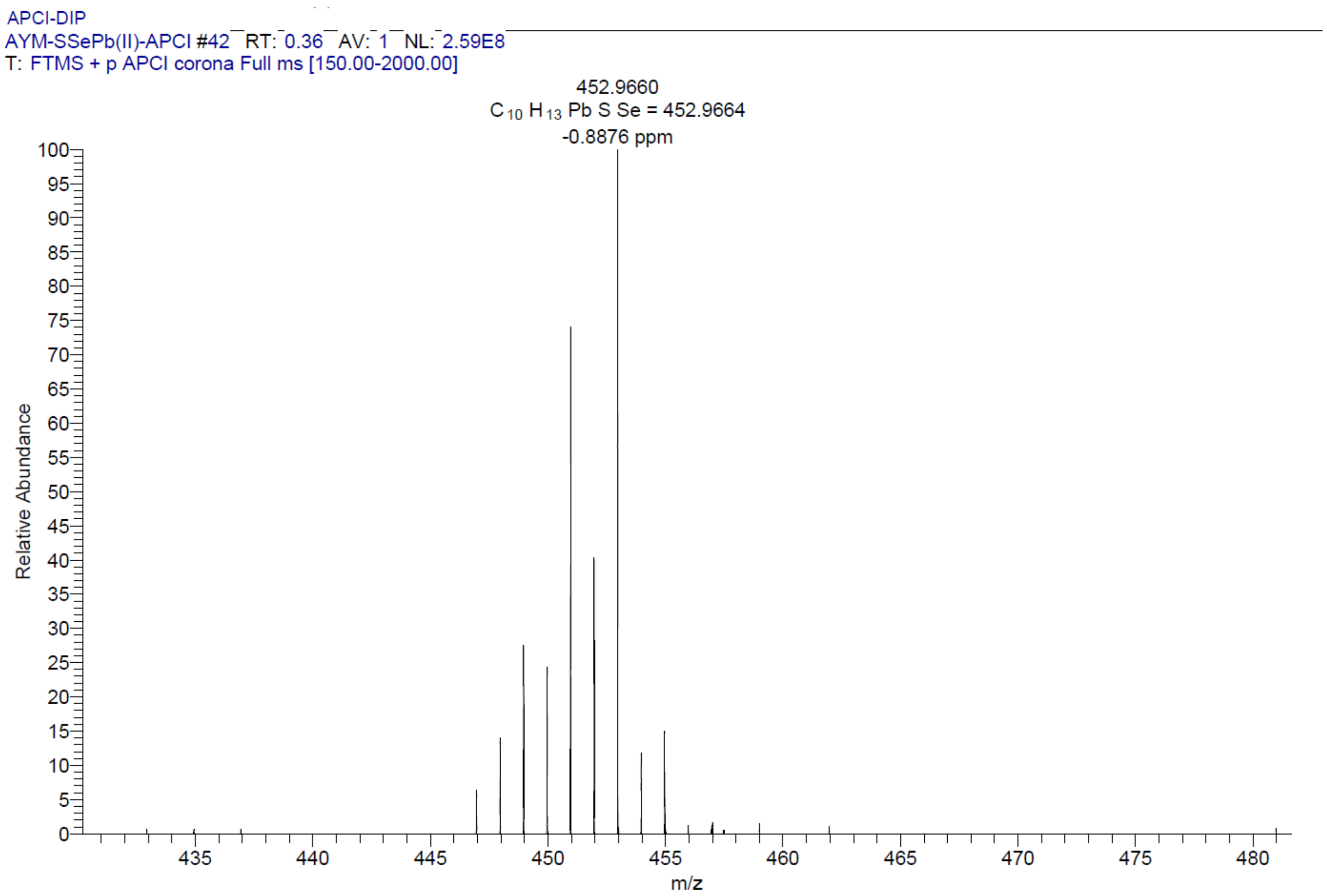

Figure S46. APCI-DIP-HRMS of compound 7a [M+H]$^{+}$.

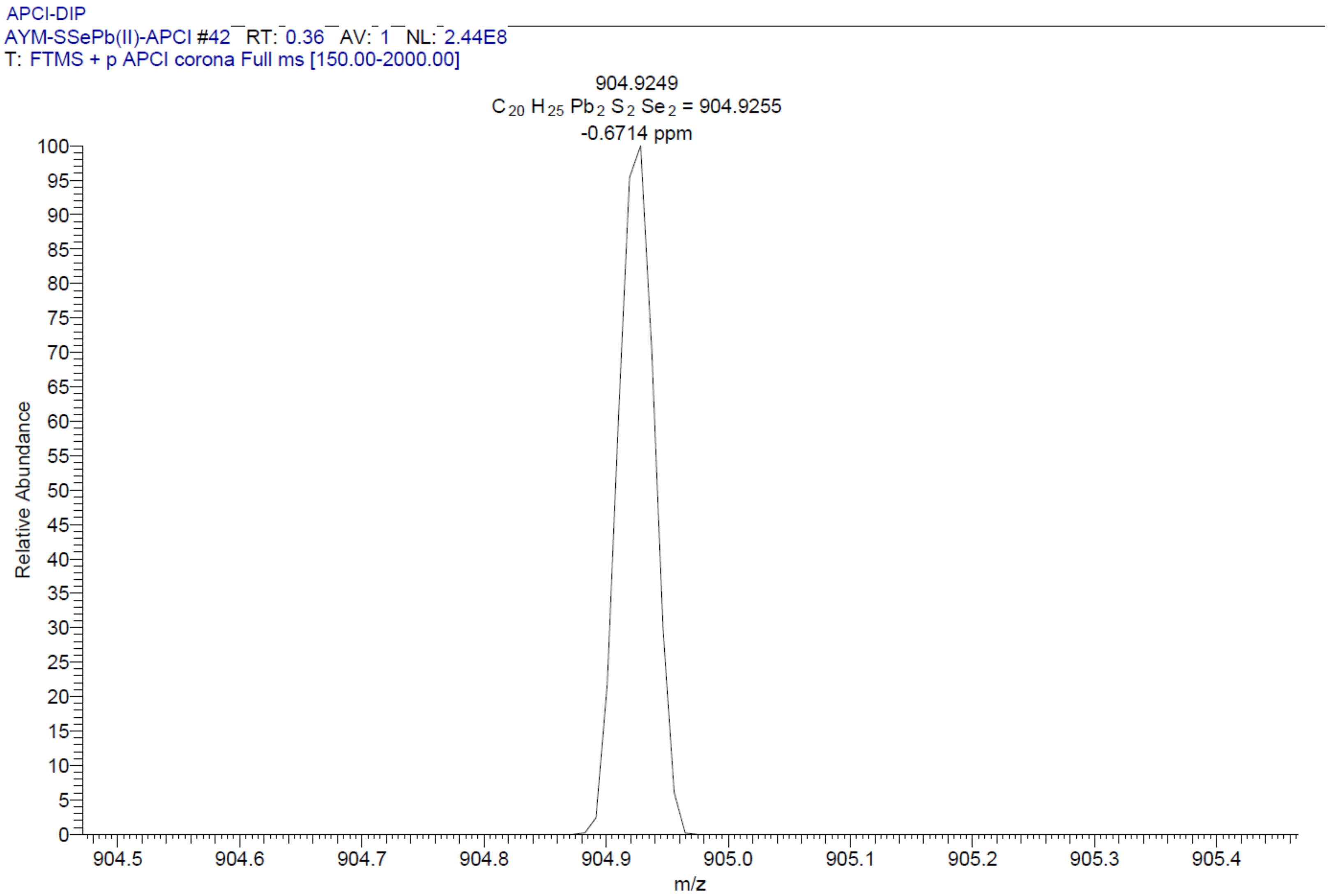

Figure S47. APCI-DIP-HRMS of compound 7a [2M+H]$^+$.

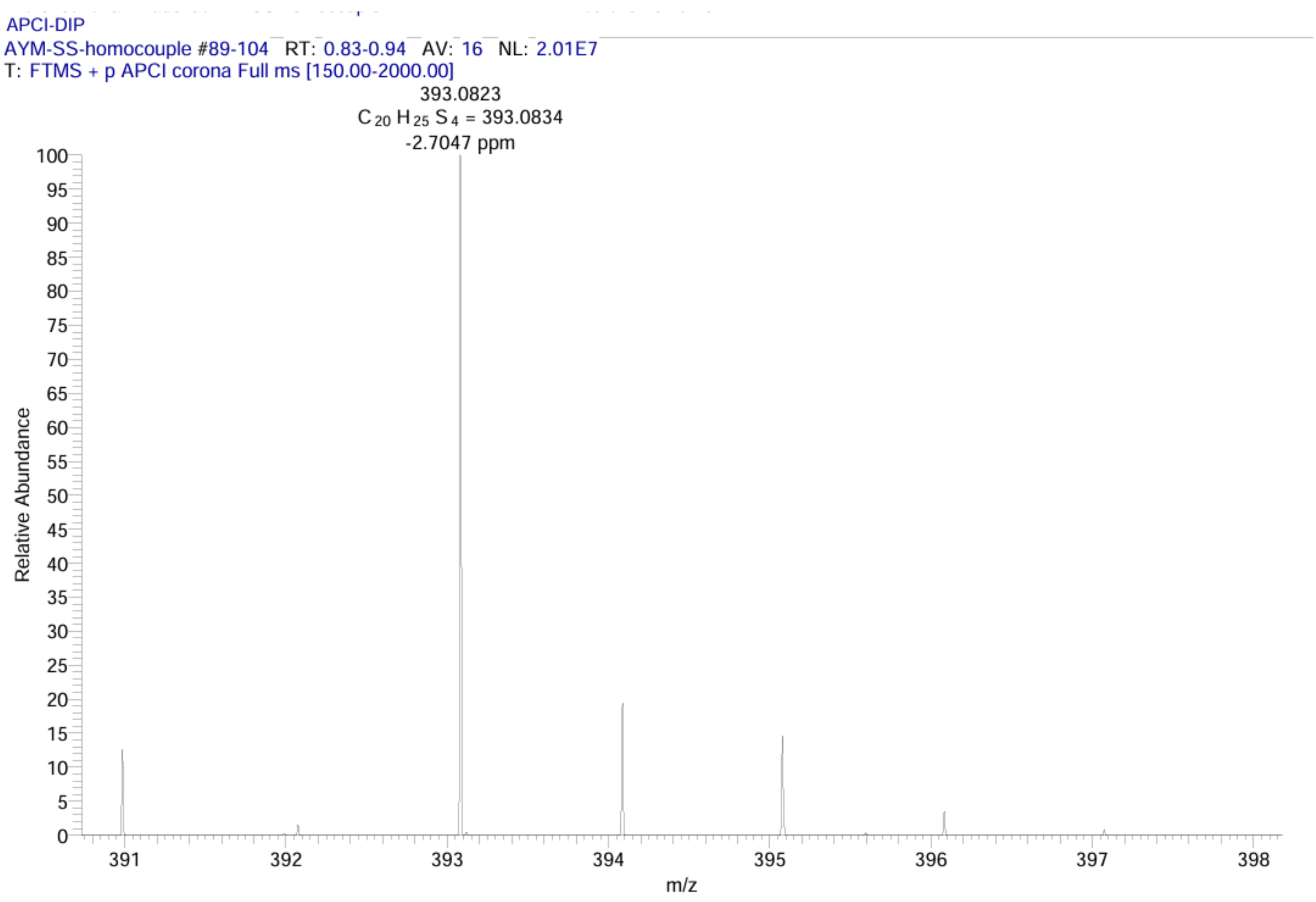

Figure S48. APCI-DIP-HRMS of compound 8 [M+H]$^+$.

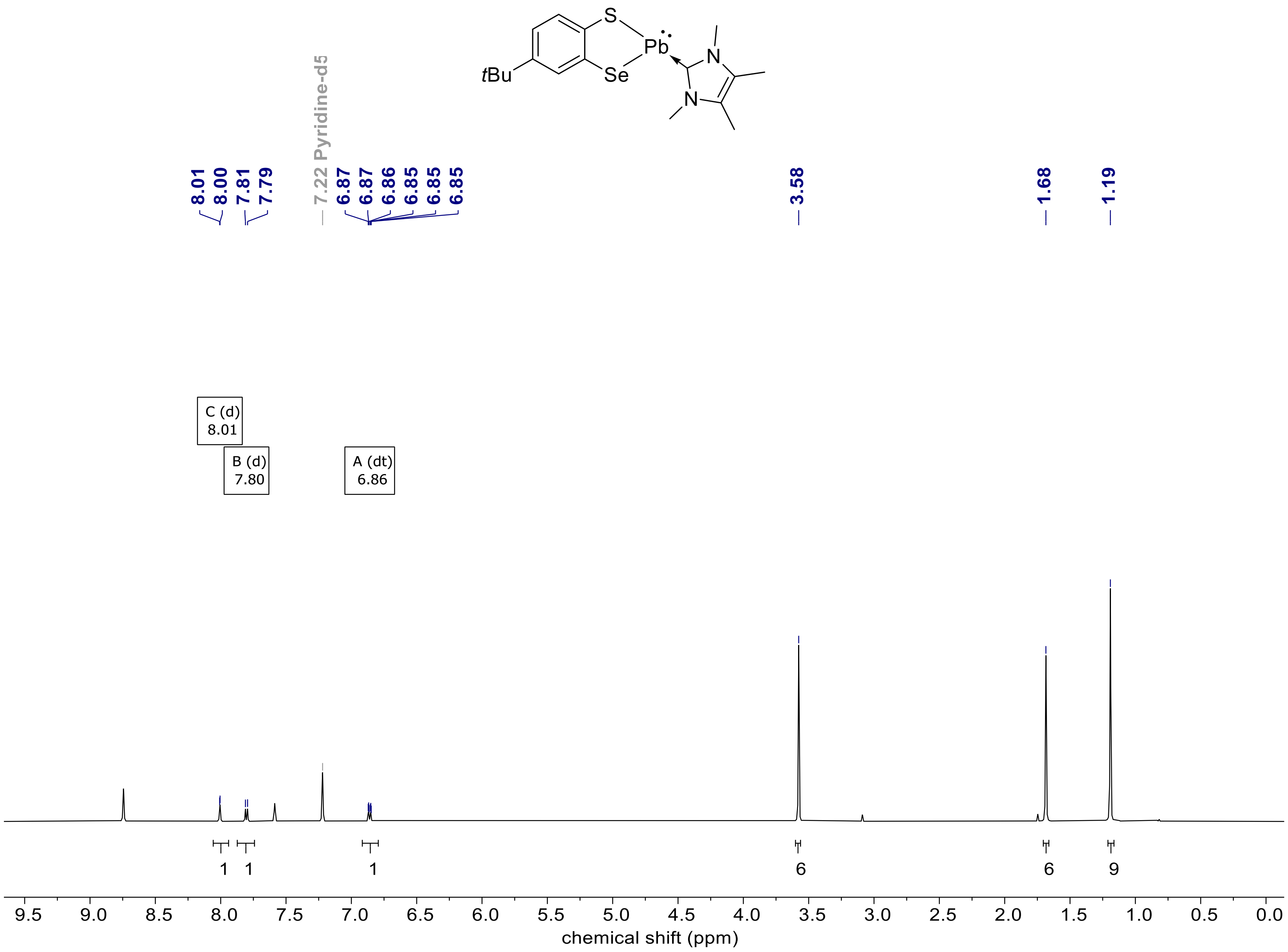

Figure S49. **^{1}H NMR spectrum (in C_5D_5N) of compound 7b.**

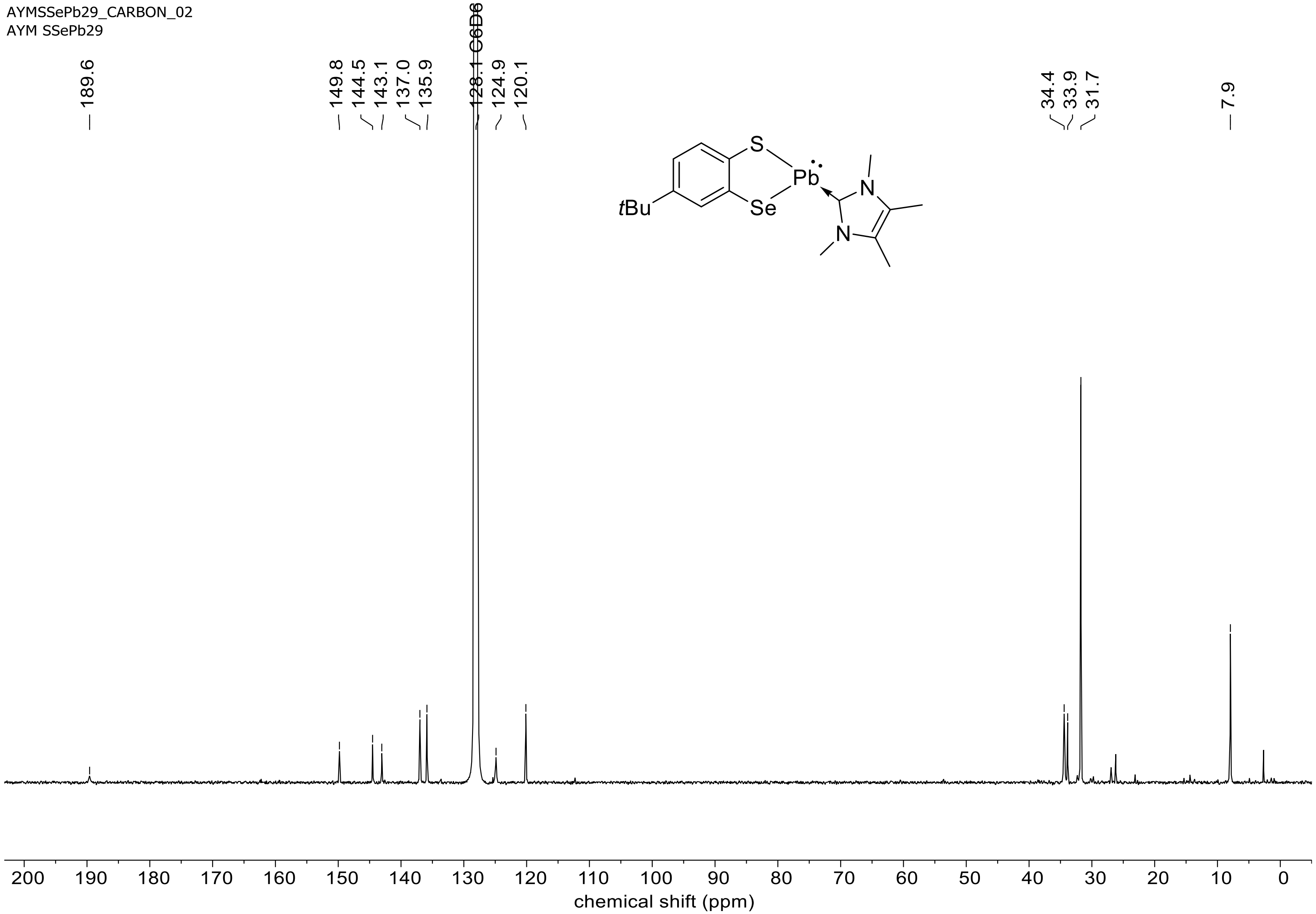

Figure S50. $^{13}C\{^{1}H\}$ **NMR spectrum (in C_6D_6) of compound 7b.**

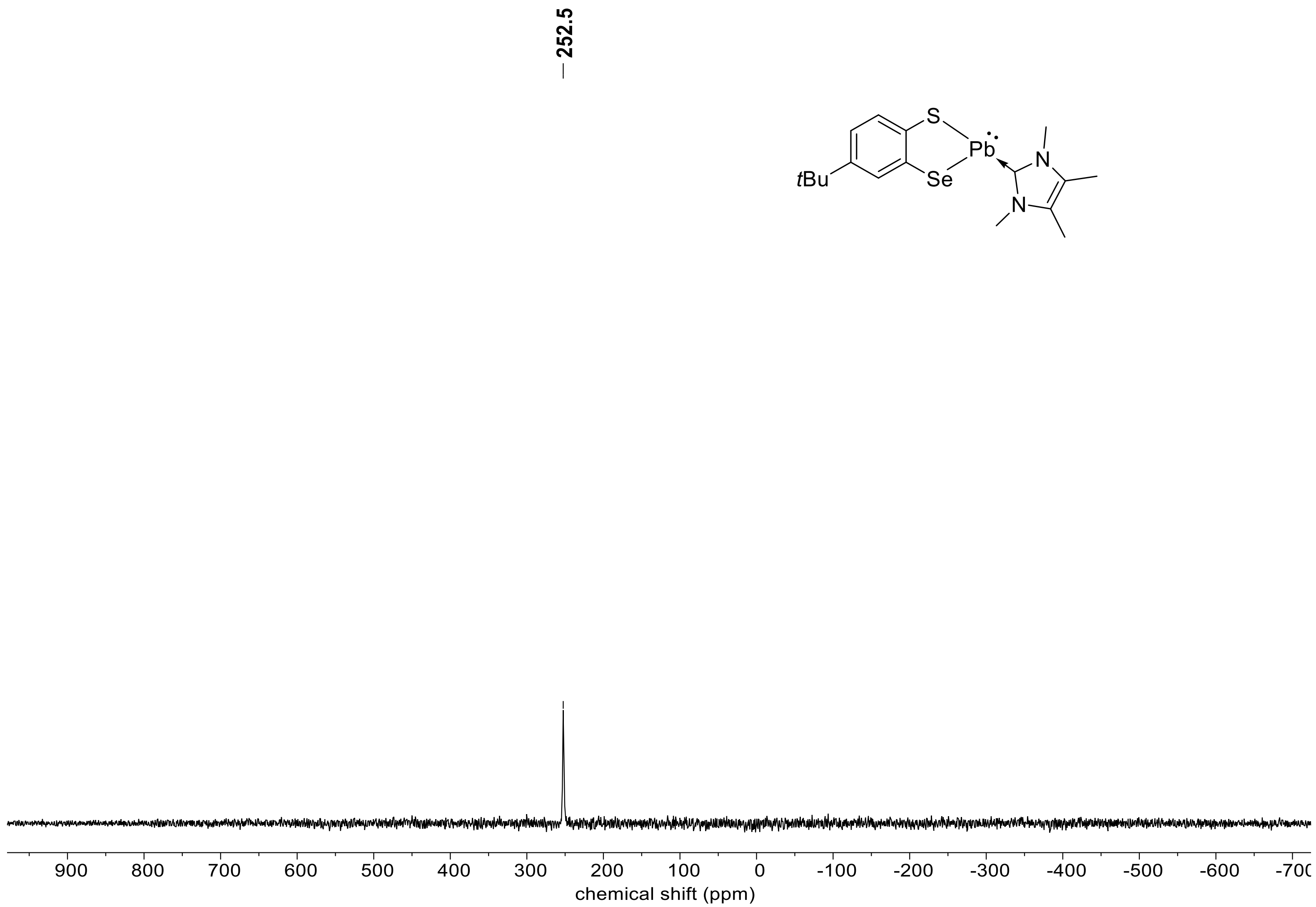

Figure S51. ^{77}Se NMR spectrum (in C_5D_5N) of compound 7b.

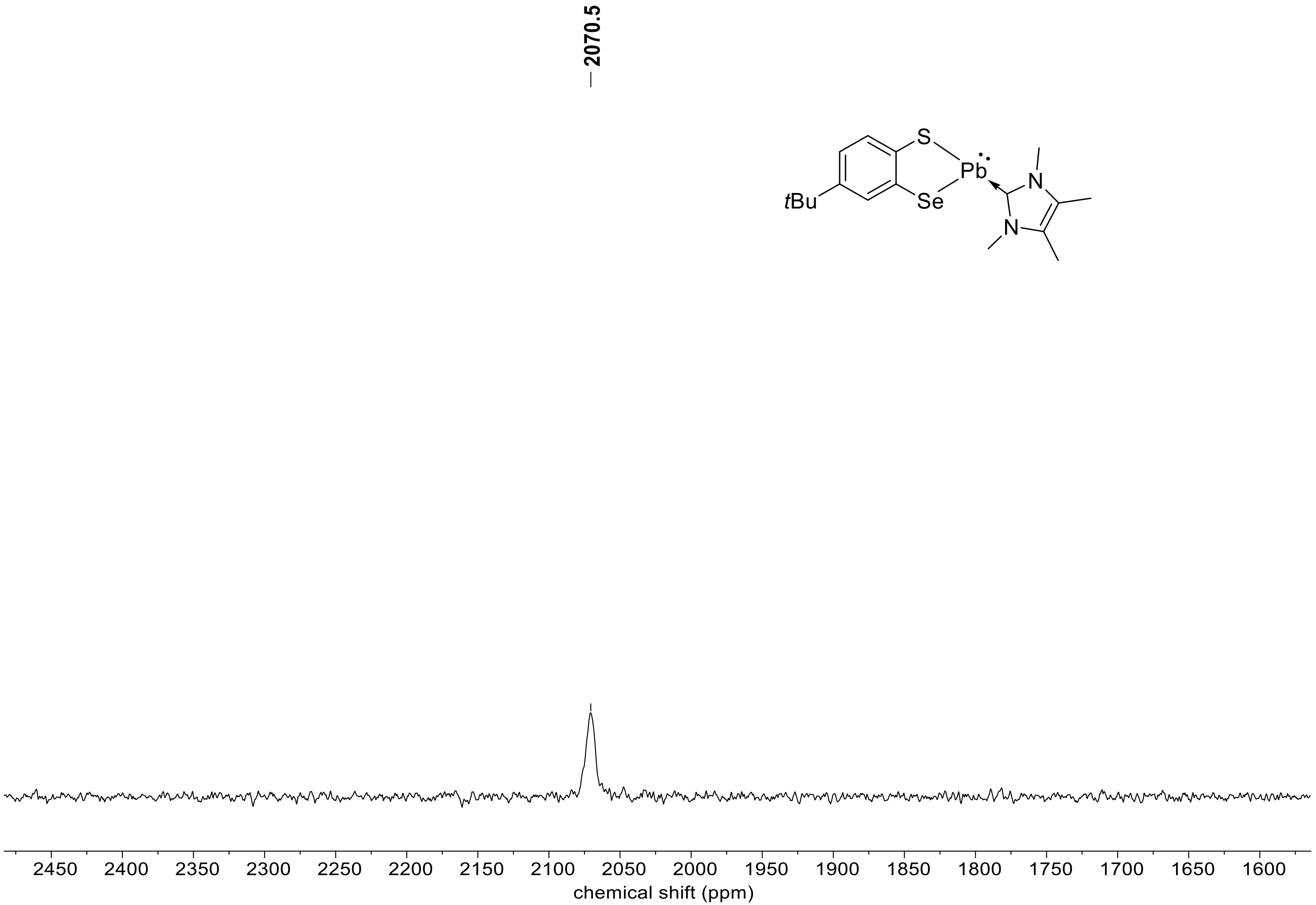

Figure S52. ^{207}Pb NMR spectrum (in Tol-d$_8$) of compound 7b.

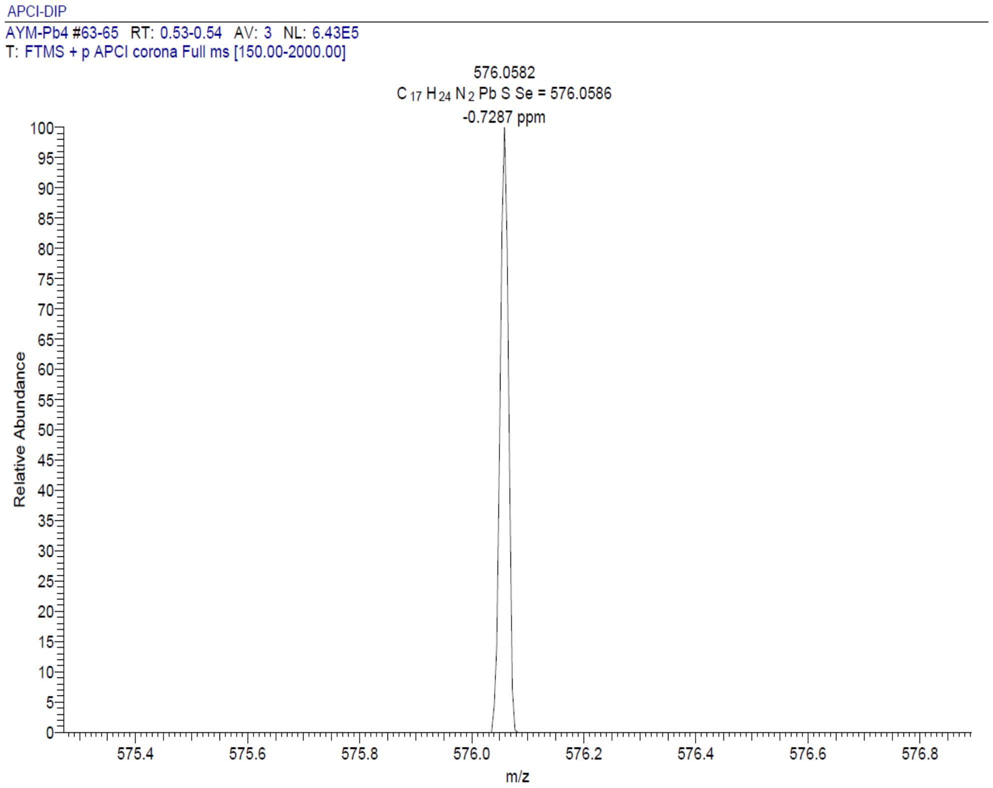

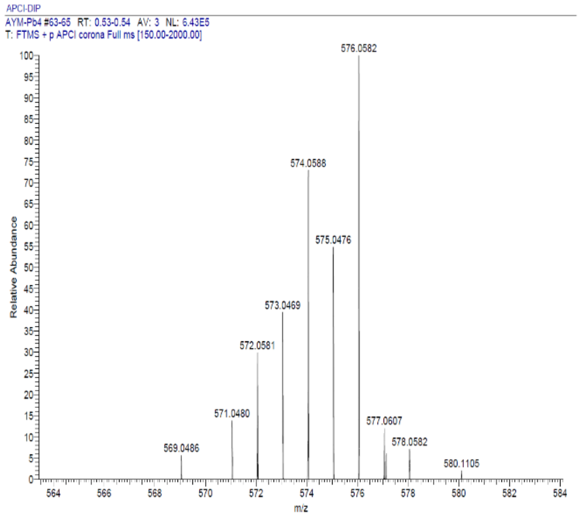

Figure S53. APCI-DIP-HRMS of compound 7b $[M]^{+}$.

2. The molecular structure of compound 7e

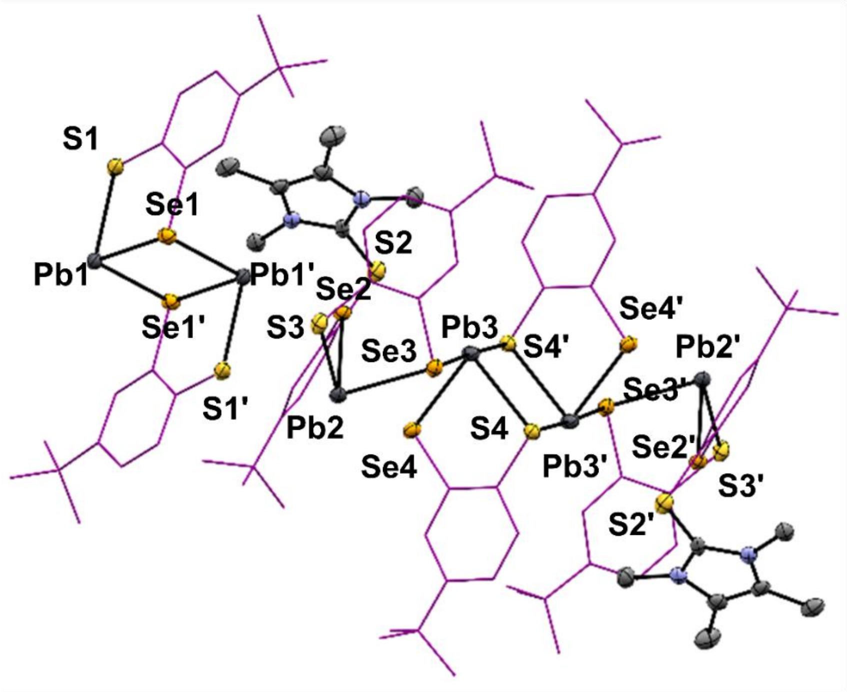

Figure S54. The molecular structure of compound 7e.

The molecular structure of compound **7e** represents polymeric structure. Hydrogen bonds and two pyridine unit are omitted for clarity. The symmetry -related portion was generated by symmetry operator. Ellipsoids are shown at 30% probability. Selected bond lengths [Å] and angles [°] in **7e**: C1-Se1 1.931(6), C2-S1 1.756(7), C28-Se2 1.923(6), C19-S2 1.778(7), S4-C39 1.777(6), Se4-C38 1.919(6), S3-C29 1.781(6), S3-C11 1.726(6), Se3-C18 1.905(6), Se1-Pb1 2.8864(7), S1-Pb1 2.6152(15), Se2-Pb1 3.2537(6), S2-Pb1 3.0987(15), Pb2-Se2 2.7965(6), Pb2-S2 2.7342(16), Pb2-Se3 2.8310(7), Pb3-Se3 3.0074(6), Pb3-Se4 2.7205(7), Pb3-S4 2.6906(15), S3-C11 1.726(6); S1-Pb1-Se1 78.54(4), Se1-Pb1-S2 143.11(3), S2-Pb2-Se2 84.85(3), Se2-Pb2-Se3 89.506(18), S2-Pb2-Se3 75.00(3), Se4-Pb3-Se3 92.366(19), S4-Pb3-Se3 84.69(3), S4-Pb3-Se4 80.89(4).

3. UV-vis Data

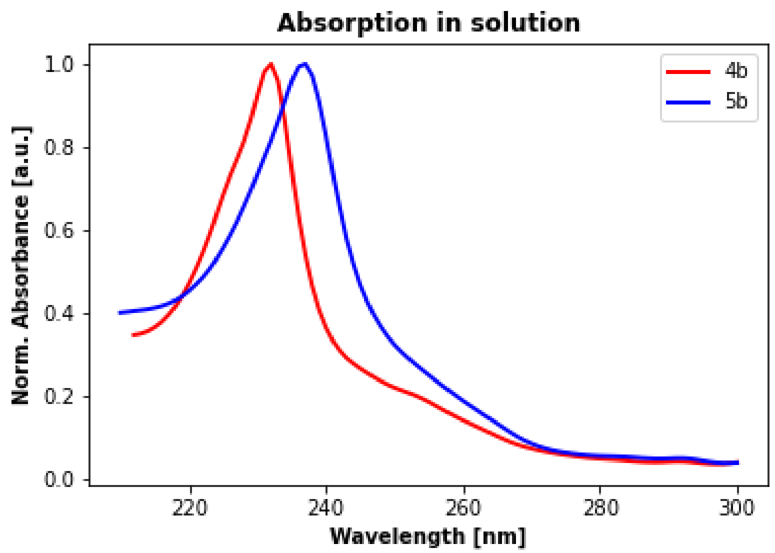

Figure S55. Normalized UV-vis absorption spectra of 4b and 5b.

Measured in n-hexane (98): THF (2) solution (c = 10 μM).

4. HPLC Data

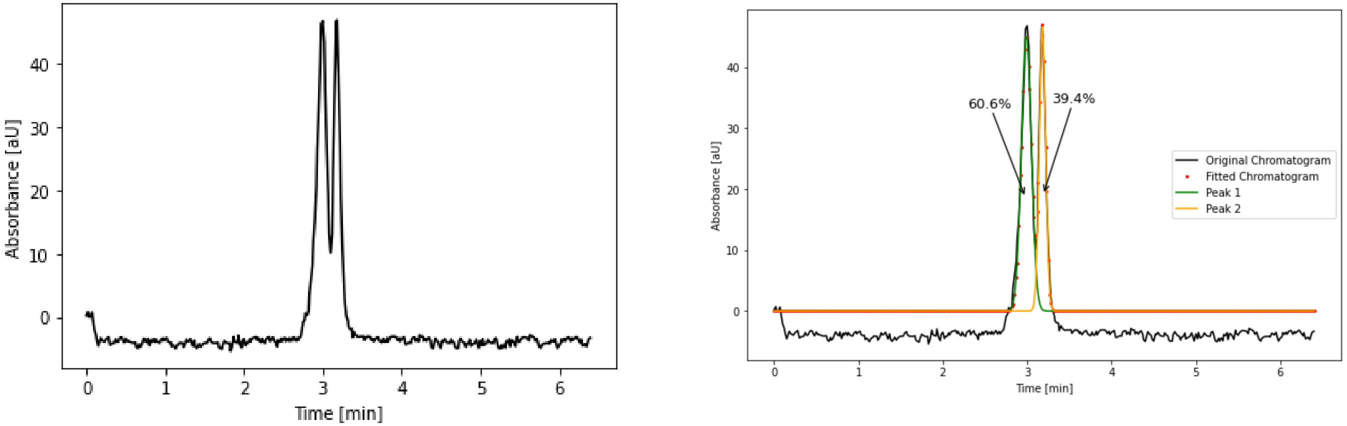

Figure S56. The analytical HPLC results of compound 5b.

The left curve from original chromatogram, right one deconvolution of the chromatogram via Python. A DAICEL's chiral Pak IJ cellulose tris-(4-methylbenzoate) (250 mm x 4,6 mm Particle: 5µm) were selected as stationary phase. Mobile phase: toluene:n-hexane (1:1). Flow rate: 1 ml/min; Detection channels (237 254 280 290 nm).

5. IR Spectra

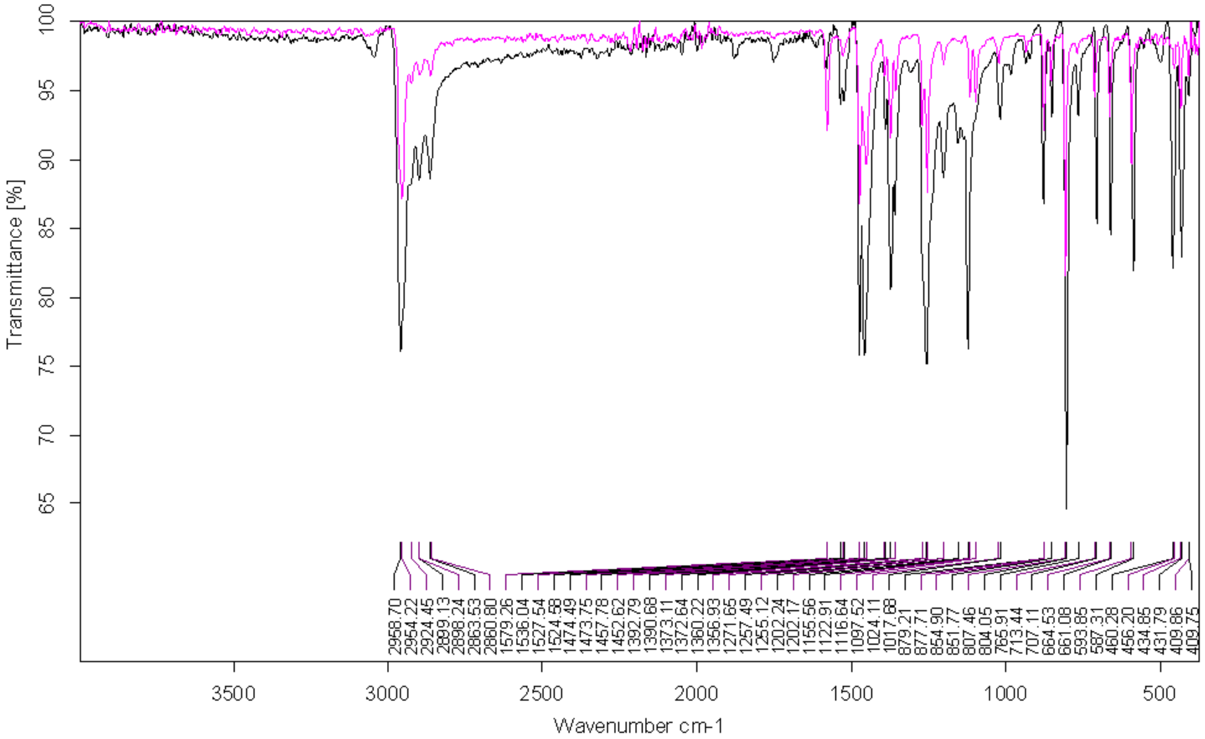

Figure S57. IR spectra of compound 5a.

The black bands correspond to the yellow solid, whereas the violet bands correspond to the violet solid.

6. Crystal data and structure refinement details.

7. Table S2. Crystal data and structure refinement for compounds 2b and 3b.

Compounds	2b	3b
**CCDC code**	2526565	2526566
**Identification code**	i3527	i3628
**Empirical formula**	$C_{22}H_{44}Li_2N_4S_2$	$C_{22}H_{44}Li_2N_4SSe$
**Formula weight [g/mol]**	441.60	489.51
**Temperature [K]**	100	100
**Crystal system**	monoclinic	monoclinic
**Space group**	$P2_1/c$	$P2_1/n$
***a* [Å]**	13.5683(14)	13.490(3)
***b* [Å]**	12.9089(11)	11.515(2)
**c [Å]**	15.9970(14)	20.731(6)
***α* [°]**	90	90
***β* [°]**	95.360(8)	94.70(2)
***γ* [°]**	90	90
**Volume [Å^3]**	2789.7(4)	3209.4(13)
**Z**	4	4
**Calculated density [g/cm^3]**	1.051	1.013
**Absorption coefficient μ [mm^{-1}]**	0.205	1.247
**F(000)**	964.0	1040.0
**Crystal size [mm^3]**	0.19 × 0.1 × 0.05	0.4 × 0.307 × 0.18
**Radiation**	Mo Kα (λ = 0.71073)	Mo Kα (λ = 0.71073)
**2Θ range for data collection [°]**	3.014 to 53.992	3.476 to 54.816
**Index ranges**	$-17 \le h \le 17$, $-16 \le k \le 16$, $-16 \le l \le 20$	$-15 \le h \le 17$, $-14 \le k \le 14$, $-25 \le l \le 26$
**Reflections collected**	13983	20909
**Independent reflections**	5986 [R_{int} = 0.0329, R_{sigma} = 0.0406]	7165 [R_{int} = 0.0374, R_{sigma} = 0.0338]
**Data/restraints/parameters**	5986/39/318	7165/0/309
**Goodness-of-fit on F^2**	1.041	0.990
**Final R indexes [I>=2σ (I)]**	R_1 = 0.0478, wR_2 = 0.1170	R_1 = 0.0539, wR_2 = 0.1492
**Final R indexes [all data]**	R_1 = 0.0740, wR_2 = 0.1308	R_1 = 0.0670, wR_2 = 0.1615
**Largest diff. peak/hole [e Å^{-3}]**	0.43/-0.39	1.08/-0.98

Table S3. Crystal data and structure refinement for compounds 4b and 5b.

Compounds	4b	5b
**CCDC code**	2526567	2526568
**Identification code**	i3692	sv1757
**Empirical formula**	$C_{20}H_{24}GeS_4$	$C_{20}H_{24}GeS_2Se_2$
**Formula weight [g/mol]**	465.22	559.02
**Temperature [K]**	100	100
**Crystal system**	orthorhombic	monoclinic
**Space group**	Pbcn	C2/c
**a [Å]**	26.3386(9)	18.0758(6)
**b [Å]**	7.8595(3)	8.6337(2)
**c [Å]**	20.8433(10)	29.7405(12)
**α [°]**	90	90
**β [°]**	90	113.967(3)
**γ [°]**	90	90
**Volume [Å^3]**	4314.7(3)	4241.2(3)
**Z**	8	8
**Calculated density [g/cm^3]**	1.432	1.751
**Absorption coefficient μ [mm^{-1}]**	1.808	7.723
**F(000)**	1920.0	2208.0
**Crystal size [mm^3]**	0.28 × 0.157 × 0.04	0.09 × 0.07 × 0.06
**Radiation**	Mo Kα (λ = 0.71073)	Cu Kα (λ = 1.54186)
**2Θ range for data collection [°]**	3.092 to 53.948	6.504 to 143.762
**Index ranges**	$-33 \leq h \leq 30$, $-9 \leq k \leq 8$, $-26 \leq l \leq 22$	$-13 \leq h \leq 22$, $-9 \leq k \leq 10$, $-36 \leq l \leq 26$
**Reflections collected**	11928	22877
**Independent reflections**	4610 [R_{int} = 0.0501, R_{sigma} = 0.0435]	4126 [R_{int} = 0.0149, R_{sigma} = 0.0085]
**Data/restraints/parameters**	4610/0/232	4126/0/232
**Goodness-of-fit on F^2**	1.069	1.231
**Final R indexes [I>=2σ (I)]**	R_1 = 0.0672, wR_2 = 0.1728	R_1 = 0.0591, wR_2 = 0.1206
**Final R indexes [all data]**	R_1 = 0.0929, wR_2 = 0.1955	R_1 = 0.0591, wR_2 = 0.1206
**Largest diff. peak/hole [e Å^{-3}]**	1.27/-1.57	3.29/-1.31

Table S4. Crystal data and structure refinement for compounds 6b and 7b.

Compounds	6b	7b
CCDC code	252659	2526570
Identification code	sv1785	sv1882
Empirical formula	$C_{30}H_{34}N_2Pb_2S_4$	$C_{17}H_{24}N_2PbSSe$
Formula weight [g/mol]	965.21	574.59
Temperature [K]	100	100
Crystal system	monoclinic	monoclinic
Space group	$P2_1$	$P2_1/c$
a [Å]	14.4212(10)	12.5638(13)
b [Å]	7.2348(3)	9.2572(8)
c [Å]	15.7489(12)	16.946(2)
α [°]	90	90
β [°]	105.165(6)	100.648(9)
γ [°]	90	90
Volume [Å^3]	1585.93(18)	1936.9(4)
Z	2	4
Calculated density [g/cm^3]	2.021	1.970
Absorption coefficient μ [mm^{-1}]	10.887	20.065
F(000)	912.0	1088.0
Crystal size [mm^3]	0.26 × 0.11 × 0.02	0.06 × 0.04 × 0.01
Radiation	Mo Kα (λ = 0.71073)	Cu Kα (λ = 1.54186)
2Θ range for data collection [°]	4.454 to 63.124	10.624 to 133.998
Index ranges	$-20 \le h \le 21$, $-10 \le k \le 8$, $-22 \le l \le 22$	$-15 \le h \le 14$, $-10 \le k \le 5$, $-20 \le l \le 20$
Reflections collected	36963	10859
Independent reflections	8710 [R_{int} = 0.0280, R_{sigma} = 0.0335]	3421 [R_{int} = 0.0878, R_{sigma} = 0.0779]
Data/restraints/parameters	8710/1/358	3421/185/288
Goodness-of-fit on F^2	1.084	1.041
Final R indexes [I>=2σ (I)]	R_1 = 0.0281, wR_2 = 0.0578	R_1 = 0.0944, wR_2 = 0.2512
Final R indexes [all data]	R_1 = 0.0373, wR_2 = 0.0602	R_1 = 0.1366, wR_2 = 0.2873
Largest diff. peak/hole [e Å^{-3}]	1.39/-1.50	3.78/-2.31

Table S5. Crystal data and structure refinement for compounds 7c and 7d.

Compounds	7c	7d
CCDC code	2526571	2526572
Identification code	sv1805	sv1818
Empirical formula	$C_{39}H_{53}N_5PbS_2Se_2$	$C_{54}H_{72}N_4Pb_2S_4Se_4$
Formula weight [g/mol]	1021.09	1635.61
Temperature [K]	100.15	100
Crystal system	monoclinic	monoclinic
Space group	$P2_1/c$	C2/c
a [Å]	16.0422(11)	23.1533(16)
b [Å]	13.6741(7)	28.5905(15)
c [Å]	19.1864(12)	9.4621(5)
α [°]	90	90
β [°]	103.629(5)	92.043(5)
γ [°]	90	90
Volume [Å^3]	4090.3(4)	6259.6(6)
Z	4	4
Calculated density [g/cm^3]	1.658	1.736
Absorption coefficient μ [mm^{-1}]	11.260	7.867
F(000)	2016.0	3152.0
Crystal size [mm^3]	0.25 × 0.1 × 0.01	0.17 × 0.07 × 0.01
Radiation	CuKα (λ = 1.54186)	Mo Kα (λ = 0.71073)
2Θ range for data collection [°]	8.018 to 138.998	4.53 to 53.998
Index ranges	$-18 \le h \le 19$, $-16 \le k \le 6$, $-20 \le l \le 23$	$-29 \le h \le 29$, $-36 \le k \le 35$, $-10 \le l \le 12$
Reflections collected	19088	20493
Independent reflections	7602 [R_{int} = 0.0574, R_{sigma} = 0.0564]	6772 [R_{int} = 0.0629, R_{sigma} = 0.0971]
Data/restraints/parameters	7602/36/502	6772/262/317
Goodness-of-fit on F^2	1.045	1.225
Final R indexes [I>=2σ (I)]	R_1 = 0.0814, wR_2 = 0.2227	R_1 = 0.0837, wR_2 = 0.1580
Final R indexes [all data]	R_1 = 0.1011, wR_2 = 0.2488	R_1 = 0.1788, wR_2 = 0.2402
Largest diff. peak/hole [e Å^{-3}]	2.67/-3.46	3.06/-3.41

Table S6. Crystal data and structure refinement for compounds 7e and 9a.

Compounds	7e	9a
**CCDC code**	2526573	2526574
**Identification code**	sv1817	i3830
**Empirical formula**	$C_{57}H_{71}N_4Pb_3S_4Se_4$	$C_{27}H_{36}N_2S_2Se_2$
**Formula weight [g/mol]**	1877.82	610.62
**Temperature [K]**	100	100
**Crystal system**	orthorhombic	triclinic
**Space group**	Pbca	P-1
**a [Å]**	18.6143(4)	7.5928(8)
**b [Å]**	33.2772(6)	11.8805(17)
**c [Å]**	20.4424(3)	16.452(2)
**α [°]**	90	73.045(10)
**β [°]**	90	78.109(10)
**γ [°]**	90	86.315(10)
**Volume [$Å^3$]**	12662.7(4)	1389.1(3)
**Z**	8	2
**Calculated density [g/cm^3]**	1.970	1.460
**Absorption coefficient μ [mm^{-1}]**	10.427	2.830
**F(000)**	7096.0	624.0
**Crystal size [mm^3]**	0.24 × 0.107 × 0.03	0.1 × 0.08 × 0.07
**Radiation**	Mo Kα (λ = 0.71073)	Mo Kα (λ = 0.71073)
**2Θ range for data collection [°]**	4.376 to 61.292	2.64 to 49.996
**Index ranges**	-21 ≤ h ≤ 26, -47 ≤ k ≤ 47, -29 ≤ l ≤ 18	-9 ≤ h ≤ 8, -12 ≤ k ≤ 14, -19 ≤ l ≤ 19
**Reflections collected**	69836	8774
**Independent reflections**	18206 [R_{int} = 0.0452, R_{sigma} = 0.0629]	4816 [R_{int} = 0.1130, R_{sigma} = 0.1352]
**Data/restraints/parameters**	18206/72/665	4816/0/308
**Goodness-of-fit on F^2**	1.013	1.028
**Final R indexes [I>=2σ (I)]**	R_1 = 0.0414, wR_2 = 0.0747	R_1 = 0.0860, wR_2 = 0.2048
**Final R indexes [all data]**	R_1 = 0.0986, wR_2 = 0.0873	R_1 = 0.1772, wR_2 = 0.2729
**Largest diff. peak/hole [e $Å^{-3}$]**	2.23/-1.07	0.94/-0.82

8. References

(1) Bachmann, S.; Neufeld, R.; Dzemski, M.; Stalke, D. New External Calibration Curves (ECCs) for the Estimation of Molecular Weights in Various Common NMR Solvents. *Chem. - Eur. J.* **2016**, *22* (25), 8462-8465. DOI: https://doi.org/10.1002/chem.201601145.
